# Supplementary material for: Performance of the Safer Nursing Care Tool to measure nurse staffing requirements in acute hospitals: a multicentre observational study
Source: BMJ Open. 2020 May 15;10(5):e035828. doi: 10.1136/bmjopen-2019-035828 (PMC7232629; doi:10.1136/bmjopen-2019-035828)
Supplement: Supplementary data [file bmjopen-2019-035828supp001.pdf]

Supplementary material to accompany article: Griffiths P, Saville C, Ball J, Culliford D, Pattison N and Monks T. Performance of the Safer Nursing Care Tool to measure nurse staffing requirements in acute hospitals: a multi-centre observational study. BMJ Open 2020. https://doi.org/bmjopen-2019-035828

Supplementary tables

Table 1 Association between staffing shortfall and nurse perceptions of staffing adequacy: test for non-linear staffing effects in multivariable models.

**Variable**	**Enough staff for quality**			**Nursing care left undone**			**Staff breaks missed**		
	**Adjusted OR**	**95% confidence interval**	**p-value**	**Adjusted OR**	**95% confidence interval**	**p-value**	**Adjusted OR**	**95% confidence interval**	**p-value**
Registered nurse shortfall (HPPD)	0.89	[0.86, 0.92]	**<0.001**	1.15	[1.09, 1.21]	**<0.001**	1.13	[1.08, 1.19]	**<0.001**
Nursing support worker shortfall (HPPD)	0.85	[0.83, 0.88]	**<0.001**	1.15	[1.08, 1.22]	**<0.001**	1.12	[1.06, 1.19]	**<0.001**
Registered nurse shortfall squared	1.00	[0.99, 1.00]	0.469	1.01	[1.00, 1.02]	**0.021**	1.00	[0.99, 1.02]	0.533
Nursing support worker shortfall squared	1.01	[1.00, 1.02]	0.102	0.99	[0.97, 1.01]	0.194	0.99	[0.97, 1.01]	0.165
Day of week- Monday (reference)									
Tuesday							0.71	[0.58, 0.88]	**0.002**
Wednesday							0.61	[0.49, 0.75]	**<0.001**
Thursday							0.82	[0.66, 1.00]	0.053
Friday							0.80	[0.65, 0.99]	**0.042**
Saturday							0.49	[0.39, 0.62]	**<0.001**
Sunday							0.80	[0.65, 0.98]	**0.031**
Ward type- other (reference)									
Surgical	0.57	[0.33, 0.96]	**0.036**	1.98	[1.10, 3.54]	**0.022**	2.07	[1.18, 3.63]	**0.011**
Proportion single rooms									
Variance partition coefficient for units	0.22			0.21			0.23		
Variance partition coefficient for hospitals	0.15			0.20			0.13		
Akaike information criterion	20715			8378			8097		
Bayesian information criterion	20779			8442			8209		

Supplementary material to accompany article: Griffiths P, Saville C, Ball J, Culliford D, Pattison N and Monks T. Performance of the Safer Nursing Care Tool to measure nurse staffing requirements in acute hospitals: a multi-centre observational study. BMJ Open 2020. https://doi.org/bmjopen-2019-035828

Table 2 Association between staffing shortfall and nurse perceptions of staffing adequacy: test for interactions between significant variables in multivariable models

Variable	Enough staff for quality			Nursing care left undone			Staff breaks Missed		
	**Adjusted OR**	**95% confidence interval**	**p-value**	**Adjusted OR**	**95% confidence interval**	**p-value**	**Adjusted OR**	**95% confidence interval**	**p-value**
Registered nurse shortfall (HPPD)	0.92	[0.89, 0.95]	**<0.001**	1.14	[1.07, 1.21]	**<0.001**	1.13	[1.02, 1.25]	**0.019**
Nursing support worker shortfall (HPPD)	0.87	[0.84, 0.90]	**<0.001**	1.14	[1.06, 1.22]	**<0.001**	1.13	[1.00, 1.28]	**0.049**
Registered nurse shortfall squared (HPPD)				1.01	[1.00, 1.02]	0.044			
Day of week- Monday (reference)									
Tuesday							0.71	[0.57, 0.90]	**0.005**
Wednesday							0.60	[0.47, 0.76]	**<0.001**
Thursday							0.84	[0.67, 1.05]	0.135
Friday							0.87	[0.69, 1.09]	0.225
Saturday							0.50	[0.38, 0.65]	**<0.001**
Sunday							0.81	[0.64, 1.01]	0.064
Unit type- Other (reference)									
Surgical	0.63	[0.37, 1.08]	0.096	2.03	[1.11, 3.69]	**0.021**	2.04	[1.15, 3.63]	**0.015**
Proportion single rooms									
Turnover (per nursing hour)									
Registered nurse shortfall : Nursing support worker shortfall	0.99	[0.98, 1.01]	0.315	1.00	[0.98, 1.02]	0.972	1.01	[0.99, 1.03]	0.202
Registered nurse shortfall : Tuesday							0.98	[0.86, 1.11]	0.708
Registered nurse shortfall : Wednesday							0.99	[0.86, 1.13]	0.840
Registered nurse shortfall : Thursday							0.96	[0.84, 1.09]	0.540
Registered nurse shortfall : Friday							0.94	[0.82, 1.07]	0.332
Registered nurse shortfall : Saturday							1.05	[0.89, 1.24]	0.558
Registered nurse shortfall : Sunday							1.04	[0.91, 1.20]	0.566
Registered nurse shortfall : Surgical unit	0.88	[0.82, 0.95]	**0.001**	1.00	[0.89, 1.13]	0.952	1.00	[0.89, 1.12]	0.961

Supplementary material to accompany article: Griffiths P, Saville C, Ball J, Culliford D, Pattison N and Monks T. Performance of the Safer Nursing Care Tool to measure nurse staffing requirements in acute hospitals: a multi-centre observational study. BMJ Open 2020. https://doi.org/bmjopen-2019-035828

Registered nurse shortfall: Proportion single rooms									
Nursing support worker shortfall: Tuesday							1.01	[0.85, 1.20]	0.905
Nursing support worker shortfall: Wed							1.04	[0.87, 1.24]	0.694
Nursing support worker shortfall: Thursday							0.95	[0.81, 1.11]	0.536
Nursing support worker shortfall: Friday							0.87	[0.74, 1.02]	0.097
Nursing support worker shortfall: Saturday							0.92	[0.76, 1.11]	0.390
Nursing support worker shortfall: Sunday							0.95	[0.80, 1.12]	0.522
Nursing support worker shortfall: Surgical unit	0.95	[0.88, 1.04]	0.263	0.98	[0.86, 1.11]	0.766	1.07	[0.94, 1.21]	0.325
Nursing support worker shortfall: Proportion single rooms									
Variance partition coefficient for units	0.22			0.21			0.23		
Variance partition coefficient for hospitals	0.14			0.20			0.13		
Akaike information criterion	20708			8384			8116		
Bayesian information criterion	20780			8464			8341		

Griffiths P, *et al. BMJ Open* 2020; 10:e035828. doi: 10.1136/bmjopen-2019-035828

Supplementary material to accompany article: Griffiths P, Saville C, Ball J, Culliford D, Pattison N and Monks T. Performance of the Safer Nursing Care Tool to measure nurse staffing requirements in acute hospitals: a multi-centre observational study. BMJ Open 2020. https://doi.org/bmjopen-2019-035828

Table 3: Association between overall (registered nurse and assistant) shortfall and nurse perceptions of staffing adequacy

**Variable**	**Enough staff for quality**			**Nursing care left undone**			**Staff breaks missed**		
	**Adjusted OR**	**95% confidence interval**	**p-value**	**Adjusted OR**	**95% confidence interval**	**p-value**	**Adjusted OR**	**95% confidence interval**	**p-value**
Overall shortfall (HPPD)	0.88	[0.86, 0.90]	**<0.001**	1.14	[1.09, 1.19]	**<0.001**	1.11	[1.07, 1.16]	**<0.001**
Skill mix (registered nurse HPPD/ total HPPD)	0.81	[0.62, 1.06]	0.117	0.74	[0.47, 1.17]	0.195	0.88	[0.57, 1.38]	0.586
Patient turnover (per care hour)	0.91	[0.30, 2.77]	0.869	3.51	[0.63, 19.65]	0.153	4.99	[0.96, 26.02]	0.057
Unit type- other (reference)									
Surgical	0.54	[0.31, 0.93]	**0.025**	2.14	[1.20, 3.83]	**0.010**	2.16	[1.23, 3.80]	**0.007**
Proportion single rooms	0.53	[0.17, 1.64]	0.271	3.01	[0.93, 9.69]	0.065	2.06	[0.65, 6.57]	0.222
Day of week- Monday (reference)									
Tuesday	1.12	[0.99, 1.26]	0.078	0.86	[0.69, 1.06]	0.159	0.71	[0.58, 0.88]	**0.001**
Wednesday	1.28	[1.13, 1.45]	**<0.001**	0.96	[0.77, 1.18]	0.676	0.61	[0.49, 0.76]	**<0.001**
Thursday	1.09	[0.96, 1.23]	0.195	0.91	[0.73, 1.13]	0.379	0.81	[0.66, 1.00]	**0.045**
Friday	1.03	[0.91, 1.17]	0.604	0.92	[0.74, 1.15]	0.481	0.79	[0.64, 0.97]	**0.028**
Saturday	1.30	[1.14, 1.48]	**<0.001**	0.75	[0.60, 0.95]	0.016	0.50	[0.40, 0.64]	**<0.001**
Sunday	1.02	[0.90, 1.15]	0.795	1.11	[0.90, 1.37]	0.316	0.82	[0.66, 1.01]	0.058
Variance partition coefficient for units	0.22			0.22			0.23		
Variance partition coefficient for hospitals	0.12			0.17			0.11		
Akaike information criterion	20700			8376			8094		
Bayesian information criterion	20812			8488			8206		

Supplementary material to accompany article: Griffiths P, Saville C, Ball J, Culliford D, Pattison N and Monks T. Performance of the Safer Nursing Care Tool to measure nurse staffing requirements in acute hospitals: a multi-centre observational study. BMJ Open 2020. https://doi.org/bmjopen-2019-035828

Table 4: Association between staffing shortfall and nurse perceptions of "enough staff for quality": comparison of coefficients from models with full sample versus model excluding the hospital Trust where recoding occurred

Variable	Model with full sample			Model excluding 'hospital D'		
	**Adjusted OR**	**95% confidence interval**	**P-value**	**Adjusted OR**	**95% confidence interval**	**P-value**
Registered nurse shortfall (HPPD)	0.89	[0.87, 0.92]	**<0.001**	0.89	[0.86, 0.91]	**<0.001**
Nursing support worker shortfall (HPPD)	0.86	[0.83, 0.89]	**<0.001**	0.84	[0.81, 0.87]	**<0.001**
Turnover (per care hour)	0.91	[0.30, 2.75]	0.863	0.83	[0.26, 2.63]	0.747
Ward type – other (reference)						
Surgical	0.54	[0.31, 0.92]	**0.023**	0.45	[0.27, 0.76]	**0.003**
Proportion single rooms	0.54	[0.18, 1.66]	0.283	0.61	[0.24, 1.57]	0.304
Day of week- Monday (reference)						
Tuesday	1.12	[0.99, 1.26]	0.079	1.20	[1.05, 1.37]	**0.008**
Wednesday	1.28	[1.13, 1.45]	**<0.001**	1.31	[1.15, 1.50]	**<0.001**
Thursday	1.08	[0.96, 1.23]	0.2000	1.13	[0.99, 1.29]	**0.071**
Friday	1.03	[0.91, 1.17]	0.610	1.08	[0.94, 1.24]	0.278
Saturday	1.29	[1.14, 1.47]	**<0.001**	1.40	[1.21, 1.61]	**<0.001**
Sunday	1.02	[0.90, 1.15]	0.811	1.03	[0.90, 1.17]	0.697

Griffiths P, *et al. BMJ Open* 2020; 10:e035828. doi: 10.1136/bmjopen-2019-035828
