# Supplementary material for: Performance of the Safer Nursing Care Tool to measure nurse staffing requirements in acute hospitals: a multicentre observational study
Source: BMJ Open. 2020 May 15;10(5):e035828. doi: 10.1136/bmjopen-2019-035828 (PMC7232629; doi:10.1136/bmjopen-2019-035828)
Supplement: Author's manuscript [file bmjopen-2019-035828.draft_revisions.pdf]

BMJ Open is committed to open peer review. As part of this commitment we make the peer review history of every article we publish publicly available.

When an article is published we post the peer reviewers' comments and the authors' responses online. We also post the versions of the paper that were used during peer review. These are the versions that the peer review comments apply to.

The versions of the paper that follow are the versions that were submitted during the peer review process. They are not the versions of record or the final published versions. They should not be cited or distributed as the published version of this manuscript.

BMJ Open is an open access journal and the full, final, typeset and author-corrected version of record of the manuscript is available on our site with no access controls, subscription charges or pay-per-view fees (http://bmjopen.bmj.com).

If you have any questions on BMJ Open's open peer review process please email
info.bmjopen@bmj.com

BMJ Open

Performance of the Safer Nursing Care Tool to measure nurse staffing requirements in acute hospitals: a multi-centre observational study

Journal:	*BMJ Open*
Manuscript ID	bmjopen-2019-035828
Article Type:	Original research
Date Submitted by the Author:	18-Nov-2019

Complete List of Authors:	Griffiths, Peter; University of Southampton, School of Health Sciences; University of Southampton, National Institute for Health Research Collaboration for Leadership in Applied Health Research and Care Wessex<br>Saville, Christina; University of Southampton, School of Health Sciences<br>Ball, Jane; University of Southampton, School of Health Sciences<br>Culliford, David; University of Southampton, School of Health Sciences; University of Southampton, National Institute for Health Research Collaboration for Leadership in Applied Health Research and Care Wessex<br>Pattison, Natalie; Royal Marsden NHS Foundation Trust, Clinical Services; University of Hertfordshire, School of Health and Social Work<br>Monks, Thomas; University of Southampton, School of Health Sciences; University of Exeter, University of Exeter Medical School

Keywords:	Quality in health care < HEALTH SERVICES ADMINISTRATION & MANAGEMENT, Human resource management < HEALTH SERVICES ADMINISTRATION & MANAGEMENT, HEALTH SERVICES ADMINISTRATION & MANAGEMENT

SCHOLARONE™
Manuscripts

I, the Submitting Author has the right to grant and does grant on behalf of all authors of the Work (as defined in the below author licence), an exclusive licence and/or a non-exclusive licence for contributions from authors who are: i) UK Crown employees; ii) where BMJ has agreed a CC-BY licence shall apply, and/or iii) in accordance with the terms applicable for US Federal Government officers or employees acting as part of their official duties; on a worldwide, perpetual, irrevocable, royalty-free basis to BMJ Publishing Group Ltd ("BMJ") its licensees and where the relevant Journal is co-owned by BMJ to the co-owners of the Journal, to publish the Work in this journal and any other BMJ products and to exploit all rights, as set out in our licence.

The Submitting Author accepts and understands that any supply made under these terms is made by BMJ to the Submitting Author unless you are acting as an employee on behalf of your employer or a postgraduate student of an affiliated institution which is paying any applicable article publishing charge ("APC") for Open Access articles. Where the Submitting Author wishes to make the Work available on an Open Access basis (and intends to pay the relevant APC), the terms of reuse of such Open Access shall be governed by a Creative Commons licence – details of these licences and which Creative Commons licence will apply to this Work are set out in our licence referred to above.

Other than as permitted in any relevant BMJ Author's Self Archiving Policies, I confirm this Work has not been accepted for publication elsewhere, is not being considered for publication elsewhere and does not duplicate material already published. I confirm all authors consent to publication of this Work and authorise the granting of this licence.

For peer review only

Performance of the Safer Nursing Care Tool to measure nurse staffing requirements in acute hospitals: a multi-centre observational study

[Short title: Performance of the Safer Nursing Care Tool]

Peter Griffiths[1,2,3], Christina Saville[1]*, Jane E Ball[1], David Culliford [1,2], Natalie Pattison[4,5,6], Thomas Monks[1,7]

[1] School of Health Sciences, University of Southampton, Southampton, Hampshire, UK
[2]National Institute for Health Research Collaboration for Leadership in Applied Health Research and Care Wessex, Southampton, Hampshire, UK
[3] Portsmouth Hospital NHS Trust, Portsmouth, Hampshire, UK
[4]Clinical Services, The Royal Marsden NHS Foundation Trust, London, UK
[5] University of Hertfordshire, School of Health and Social Work, Hatfield, Hertfordshire UK
[7] University of Exeter Medical School, University of Exeter, Exeter, Devon, UK

*Corresponding author: C.E.Saville@soton.ac.uk 02380 595282

For peer review only - http://bmjopen.bmj.com/site/about/guidelines.xhtml

Performance of the Safer Nursing Care Tool to measure nurse staffing requirements in acute hospitals: a multi-centre observational study

Abstract

Objectives

The best way to determine nurse staffing requirements on hospital wards is unclear. This study explores the precision of nurse staffing requirements estimated using the Safer Nursing Care Tool (SNCT) patient classification system, and investigates whether recommended staff levels correspond with professional judgements of adequate staffing.

Design

Observational study linking datasets.

Setting

81 medical/surgical units in 4 acute care hospitals

Participants

22,364 unit days where staffing levels and SNCT ratings were linked to nurse reports of staffing adequacy

Primary outcome measures

SNCT-estimated staffing requirements and nurses' assessments of staffing adequacy.

Results

The recommended minimum sample of 20 days allowed the required number to employ (the establishment) to be estimated with a mean precision of 4.1%. For most units, much larger samples were required to estimate establishments within +/- 1 whole-time-equivalent staff member. Every registered nurse hour per patient day

For peer review only - http://bmjopen.bmj.com/site/about/guidelines.xhtml

shortfall in staffing was associated with an 11% decrease in the odds of nurses reporting that there were enough staff to provide quality care and a 14% increase in the odds of reporting that necessary nursing care was left undone. No threshold indicating an optimal staffing level was observed. Surgical specialty, patient turnover and more single rooms were associated with lower odds of staffing adequacy.

Conclusions

The SNCT can provide reliable estimates of the number of nurses to employ on a unit, but larger samples than the recommended minimum are usually required. The SNCT provides a measure of nursing workload that correlates with professional judgements, but the recommended staffing levels may not be optimal. Some important sources of systematic variations in staffing requirements for some units are not accounted for. SNCT measurements are a potentially useful adjunct to professional judgement, but cannot replace it.

Trial registration

ISRCTN12307968.

[words 287]

Article summary

Strengths and Limitations of this study

- In contrast to most studies of nurse staffing tools, this was a large-scale study conducted in 81 units in 4 hospitals over a year.
- This is the first study to provide an independent evaluation of this tool, which is used in most hospitals in England.
- The observational study found associations between staffing shortfalls measured using the SNCT and subjective professional assessments of staffing adequacy but did not explore the impact on objective care outcomes.

For peer review only - http://bmjopen.bmj.com/site/about/guidelines.xhtml

Introduction

In acute care hospitals, the ability to determine the 'right' number of nursing staff to employ and to deploy on any given shift is an imperative, as nurse staffing levels influence both efficiency and quality of care delivery. On the one hand, professional nurses and nursing support staff form the largest group of staff and the largest variable costs faced by hospitals. Nursing budgets are thus frequently targeted in the drive for cost savings.[1] On the other hand, inadequate nurse staffing is linked to deficits in the quality and safety of care.[2] However, despite the existence of many tools to determine staffing requirements and an extensive literature, evidence about the ability of any tool to reliably and accurately estimate staffing requirements is extremely limited.[3, 4] In this paper, we consider the "Safer Nursing Care Tool" (SNCT), which is used in the majority of acute hospitals in the United Kingdom's National Health Service(NHS) [5] and endorsed by the National Institute for Health and Care Excellence (NICE),[6] the body that produces evidence based guidelines for the NHS. We explore the reliability and precision of the estimates of required staffing establishments (that is the number of nurses to employ for a hospital unit) made using the tool and the extent to which estimated staffing requirements correspond with professional judgement of sufficient staffing. Despite the tool's widespread use and the importance of these considerations, these factors have not been previously studied.

Associations between higher registered nurse staffing levels in hospitals and improved care quality have been demonstrated in many studies. [2, 7-9] Outcomes include lower risks of in-hospital mortality [10], shorter lengths of stay [11] and fewer omissions of necessary care.[12] Such findings have underpinned policies to make minimum nurse staffing levels mandatory in some jurisdictions, most notably

For peer review only - http://bmjopen.bmj.com/site/about/guidelines.xhtml

California in the USA. Yet studies showing associations between nurse staffing levels and outcomes rarely provide a clear indication of how many staff are needed for different patients, despite evidence that needs can vary considerably, nor do most studies demonstrate tipping points in relationships, which could be one indication of an optimal staffing level.[2]

Consequently, tools and systems to guide decisions about the number of nursing staff to employ, or to deploy on any given shift are still widely used either in conjunction with or as an alternative to mandatory minimums. At the heart of most tools is some form of patient level assessment, which is translated into a measure of required nursing time.[3, 4] Many such tools exist, although they are largely unsupported by robust evidence. Studies used to support the validity of tools to determine staffing requirements simply tend to show that staff demand estimated using a given tool correlates with some other measure of demand. In the absence of a gold standard, and without addressing whether the staffing according to the predicted level is sufficient to deliver the required care, such evidence is significantly limited. Different tools, while providing results that are highly correlated, can and do give dramatically different estimates of the staffing required by the same group of patients.[4] For example applying a new system to estimate the staffing required for low acuity wards resulted in an estimate that was double that derived from the existing system.[13]

Furthermore, although a key driver for choosing to use a tool is the assertion that variable patient need cannot be efficiently met by fixed staffing levels,[14] little consideration has been given to the impact of variation on the resulting estimates of average staffing requirements. Inter-rater reliability and agreement is often reported,

For peer review only

For peer review only - http://bmjopen.bmj.com/site/about/guidelines.xhtml

the precision with which the unit staffing requirement as a whole is estimated, either on a given day or over time, is not. [4]

The SNCT [15] is reported to be used in 80% of National Health Service acute hospitals in England.[5] The tool was originally designed to determine the required number of staff to employ (the establishment) for each unit unit to ensure that there are sufficient staff to fill daily rosters to meet average need, but it is increasingly used to monitor and determine daily demand for staff. It is an example of a patient classification system.[4] Patients are classified into one of five groups, based on their acuity and dependency on nursing care, with each group having an associated weighting (described as a 'multiplier') indicating the number of nursing staff required.[15] At the time of writing, the most recently published multipliers for general adult inpatient units were based on observations of 40,000 patient care episodes.[6] The multipliers represent the average of staff time to provide all direct patient care and ancillary work for patients in each group with allowances made for annual leave, study time and sickness absence when determining the number of nurses to employ. [15]

The SNCT has been shown to correlate strongly with an alternative classification system and high inter-rater agreement is reported. [16, 17] However, while the tool's handbook recommends a sample of at least 20 days to establish a reliable baseline for setting establishments, we could find no evidence of the precision of the resulting estimates. In our review of literature, we found no direct evidence that using this or any other tool improved the quality of care. [4]

This observational study aims to provide evidence about the reliability and validity of the SNCT by addressing the precision of the estimated establishment and the extent

For peer review only

to which staffing shortfalls relative to the level implied by the tool is associated with nurses' judgement that staffing levels are insufficient to sufficient to deliver all necessary care with acceptable quality. Because factors such as patient turnover, specialty and layout are not directly considered in patient classifications yet may influence staffing requirements [18-20], we also examine the extent to which staffing levels determined using the SNCT are sufficient to accommodate variation in demand associated with these factors by determining whether there is an independent association between these factors and judgements of staffing adequacy when considering the effect of shortfalls from the SNCT recommended staffing level.

Methods and materials

We used routinely collected data and nurse reports over one year (2017) from 81 acute medical/surgical units (2178 beds) in four NHS hospital trusts (hereafter referred to as hospitals for brevity) in England. For each unit and for each day we identified staffing measurements: the staffing level deployed (from the electronic roster), the staffing level required (based on patient classifications using the SNCT), and nurses' professional judgement of the completeness of care and adequacy of staffing to deliver quality (through a micro survey embedded in the daily assessments). SNCT and staffing adequacy assessments were provided by the nurse in charge of the shift, hereafter referred to as 'shift leader' for brevity.

Setting & inclusion

The study sites were one university teaching hospital, two general hospitals and a specialist cancer hospital (two sites) based in London, South East and South West England. The hospitals serve diverse populations including rural areas, deprived inner city populations and specialist national referrals. All hospitals undertook reviews of nurse staffing establishments at least twice a year. Two had been using

For peer review only - http://bmjopen.bmj.com/site/about/guidelines.xhtml

the SNCT as part of this process for some time while two adopted it shortly before the study commenced.

We included general medical and surgical units that provided 24 hour inpatient care. Services out of scope of the SNCT (e.g. paediatrics, intensive care, maternity, neonatal and palliative care), and any others with highly atypical staffing requirements (e.g. bone marrow transplant and isolation units), as determined by a local co-investigator, were excluded. Our unit sample represented 74% of all beds across the four hospitals.

Data sources and measures

Over the course of one year at least twice per day, shift leaders recorded the number of patients in each SNCT category and made judgements about staffing adequacy (see below) in electronic systems. Local leads trained potential shift leaders on participating units in the use of the SNCT and completion of the staffing adequacy questions. Supporting information and brief guidance was provided on laminated sheets kept near the unit computers where data were entered. Other data for the study were routinely collected for administrative purposes (roster, patient admissions or discharges). Each hospital supplied a profile for each unit with main speciality and layout including the number of beds / single rooms.

Study variables

We used the most up-to-date SNCT multipliers available at the start of the study.[15] We took the reported counts of patients in each category and calculated the weighted average multiplier per unit and day. We multiplied this by the patient count derived from the patient administration system, in case any patients were omitted from shift leader reports. This figure provides an estimate of the required unit

For peer review only - http://bmjopen.bmj.com/site/about/guidelines.xhtml

establishment (number of staff to employ). We used morning assessments (substituting later assessments from the same day if missing) and patient count at 7am for our main analysis. To determine the implied daily hours, we used a 37.5 hour working week for 1 whole time equivalent, removing the 22% 'uplift', which is added to the SNCT establishment to account holidays, study and sick leave.

For each unit we used the average observed skill mix on that unit as a proxy for the planned skill mix of registered nurses and nursing assistants. The SNCT does not directly account for patients identified as requiring 1 to 1 supervision, often referred to as 'specialing' [21] even though the implied staffing requirement is very high. Therefore, where we wanted to identify the staffing requirements for any particular day, we identified the number of such patients from records and added the required hours to our estimated staffing requirement. However, because such enhanced care would form part of the care observed to determine the SNCT multipliers and thus be included in the average, we made no additional allowance when estimating establishments to be employed.

From the electronic roster we identified hours worked by registered nurses and nursing assistants each day (from 7am to 7am) and divided these by the number of patient days (patient hours / 24) to calculate hours per patient day (HPPD) for each unit for each day. We calculated a measure of staff shortfall, by subtracting the required hours (according to the SNCT plus specialing requirements) from the hours actually deployed on that day. If more staff than the estimated requirement were deployed, the shortfall was negative. We also calculated daily patient turnover per staff member (the numbers of patients entering and leaving units divided by the total staff hours).

For peer review only

For peer review only - http://bmjopen.bmj.com/site/about/guidelines.xhtml

The outcomes measured were a number of variables reflecting the adequacy of nurse staffing, as reported by the nurse in charge of the shift ('shift leader'). The shift leader responded to three brief items every time they provided SNCT ratings, directly inputting responses into the same system as used for SNCT (see Table 1). Two items, based on the widely used RN4CAST / International Hospital Outcomes surveys of nurse staffing and quality, asked whether there were enough staff for quality and whether any necessary care was left undone. [12, 22] We also asked about staff missing breaks, as nurses may miss breaks to complete care activities, creating additional staff time that avoids adverse effects of staffing shortfalls.[23] These questions constituted the micro-survey.

Table 1 Staffing adequacy questions

**Questions**
Were there enough nursing staff to provide quality care on the last shift?
Was necessary nursing care left undone (missed) on the last shift because there were too few nursing staff?
Were staff breaks missed on the last shift because there were too few nursing staff?

Data cleaning and Analysis

Data cleaning, processing and statistical analyses were carried out in R statistical software V3.5.0.[24] We identified and removed extreme values of staffing shortfall, where values lay outside the mean plus or minus three standard deviations (approximately 1.5% of cases). This removed atypical periods if the unit was not functioning as normal, e.g. over the Christmas period, or where there is an extreme error in the recorded SNCT ratings. Where there were major changes such as unit moves, changes to the patient population or bed numbers, data for that unit were split and treated as separate units. We found some evidence of consistent reverse coding of data inputs (0/1 for yes/no) for some staffing adequacy questions in several units of one hospital. This appeared to result from erroneous staff training. Because it was discovered partway through the study we developed logical rules to

For peer review only - http://bmjopen.bmj.com/site/about/guidelines.xhtml

identify units where this occurred and recode data, considering the implications of this through sensitivity analyses where we excluded the hospital entirely.

To understand the accuracy of the estimated establishment we first considered the minimum recommended sample size of the SNCT data collection, which is 20 days twice a year. We used 1000 bootstrap samples of 20 days' data to estimate a mean establishment with a 95% confidence interval for the establishment on each unit. We repeated this with bootstrap samples of increasing numbers of days to assess the accuracy of larger samples. For each unit, we calculated both the precision of the estimate, that is half the width of the confidence interval expressed as a % of the mean, and the absolute value of the confidence interval in whole time equivalent (WTE) staff members. We determined the number of units where the confidence interval of the establishment was 2 WTE or less (that is no more than +/-1 WTE difference from the mean) or 1 WTE or less (mean +/- .5).

We modelled the relationship between staffing deficits (in HPPD) and nurse-reported measures of staffing adequacy. We fitted multilevel logistic regression models for binary outcomes using the glmer (generalised linear mixed effects regression) function from the lme4 package [25] in R. Staffing was nested in unit which was nested in hospital. All models included control for day of the week, proportion of single rooms, turnover and unit specialty (surgical vs medical or mixed). We considered the association of staffing adequacy outcomes with deviation of both registered nurse staffing and nursing assistant staffing from their estimated requirements. We also fitted models using the deviation in total hours and skill mix (registered nurse proportion).

For peer review only - http://bmjopen.bmj.com/site/about/guidelines.xhtml

After modelling the linear and main effects, we introduced quadratic terms for the staffing level variables to assess non-linear relationships and we investigated whether staffing variables interacted with other variables. We compared the fit of models using the Akaike and Bayesian information criteria (AIC, BIC), preferring models with lower values, indicating better fit / more parsimonious models.[26]

Patient and public involvement

When developing our research proposal, we discussed appropriate patient/public involvement with Claire Ballinger (Patient and Public Involvement lead for the NIHR CLAHRC Wessex) and Anya de Iongh (Patient and Public Involvement champion for the 'fundamental care in hospital' theme of the CLAHRC). Following their guidance we sought no further direct patient / public involvement in prioritising or shaping the questions as these arose from the brief and need for technical assessment of the tool, instead we focussed on considering how the public could be involved in the proposed research, its governance and dissemination.

Based on this advice, we sought a lay member of our steering group with specific interest and expertise. To this end, we worked with Stephen Habgood, a lay member of the NICE safe staffing advisory committee for the development of guidelines in mental health, who agreed to participate in the project steering group. Stephen also has experience of staffing methodologies used in other sectors from his past work as a prison governor and is currently chair of a mental health charity. Additionally, and guided by the advice from our patient and public involvement experts, we also considered ward-based staff nurses as the potential end users of our research in a way that is analogous to a patient receiving a treatment that might be recommended by an expert. Based on this we have used multiple channels to connect with ward-

For peer review only

based staff including extensive use of social media and discussions with individual staff nurses at consultation events.

Ethical approval and registration

Ethical approval was granted by the University of Southampton Ethics committee (reference 18809). The study was prospectively registered (ISRCTN 12307968).[27]

Results

We had useable SNCT ratings on 96% of occasions and responses to staffing adequacy questions on 85% or more of possible occasions. After data cleaning and linkage, we had 22,271, 22,294 and 22,364 unit days where we could assess the association between staffing shortfalls and reports of staff breaks missed, nursing care left undone and enough staff for quality respectively.

Average unit staffing levels and skill mix varied considerably between hospitals and between units within hospitals (Table 2). At a hospital level, average estimated staffing requirements of units corresponded closely with the observed staffing levels in 3 of 4 hospitals although all were somewhat understaffed relative to the estimated requirement (8% or less). Larger differences between actual staffing and SNCT estimates occurred on smaller, generally specialist, units with more single rooms (where apparent overstaffing occurred), and some larger medical units (where extremes of apparent understaffing occurred). In hospital C, a specialist hospital with many small units, average unit staffing was 50% higher than the SNCT estimated requirement.

For peer review only - http://bmjopen.bmj.com/site/about/guidelines.xhtml

Table 2 Mean and range of unit's average daily staffing levels, skill mix, SNCT estimated staffing requirements.

Hospital	Total hours per patient day			Skill mix (% registered nurses)			Estimated staffing requirement		
	Mean	[Min	Max]	Mean	[Min	Max]	Mean	[Min	Max]
A	7	[5.4	10.4]	51%	[42%	70%]	7.4	5.9	10.2
B	6.8	[5.0	8.9]	56%	[39%	79%]	7.3	6.0	9.4
C	10.5	[7.5	14.2]	75%	[70%	78%]	7	6.3	7.4
D	6.5	[5.2	8.4]	49%	[40%	63%]	7	6.5	7.6
All	7.3	[5.0	8.4]	56%	[39%	79%]	7.2	5.9	10.2

Across all units, using the recommended minimum of 20 days' data, the average precision was 4.1% but varied by unit (range 0.6% - 13.5%). In absolute terms, the average width of the 95% confidence intervals for the establishment was 2.9 whole time equivalent staff (that is approximately mean +/- 1.5 WTE). The confidence interval width was ≤2 WTE in 27/86 units and ≤1 WTE in only 3/86.

As the number of days sampled increases from 20 there was a marked increase in precision (Figure 1), with most units (56/86) yielding a confidence interval width of ≤2 WTE from a sample of 40 days. The benefits of increased sample sizes diminishes with larger samples however, and even with samples of 180 days only 53/77 units gave a confidence interval width that was ≤1 WTE wide (Table 3).

Fig 1 Mean precision and confidence interval width of staffing establishment estimates with different sample sizes

For peer review only

For peer review only - http://bmjopen.bmj.com/site/about/guidelines.xhtml

325 Table 3 Average widths of 95% confidence intervals for the mean using different sample sizes to
326 estimate establishment

Sample size taken for the estimate	Average confidence interval width (WTE)	Average precision	Number units with confidence interval width 1 WTE or less	Number units with confidence interval width 2 WTE or less	Number of units[1]
**20**	2.9	4.2%	3	27	86
**40**	2.1	3.0%	7	56	86
**60**	1.7	2.5%	10	64	86
**80**	1.5	2.1%	20	72	86
**100**	1.3	1.9%	31	74	82
**120**	1.2	1.8%	39	74	81
**140**	1.1	1.6%	44	74	81
**160**	1.0	1.5%	50	75	80
**180**	1.0	1.4%	53	73	77

1. Because units with establishment and / or specialty changes were treated as separate units for analysis, the total exceed the number of units participating in the study. As the available data for some units was less than the sample required for the estimate, the number of units for larger samples is reduced.

327

328 Across all units, a mean of 78% of shifts were assessed by the nurse in charge as
329 having enough staff to deliver quality care (unit mean range 24% to 96%). Necessary
330 nursing care was reported left undone because of too few staff on 5% of shifts
331 (range 0-25%) and breaks were reported missed on 5% of shifts (range 0-29%).

332 Shortfalls in staffing levels relative to the requirement for that day, estimated using
333 the SNCT, were associated with nurses' perceptions of staffing adequacy (Table 4).
334 In the multivariable models, for each registered nurse hour shortfall, the adjusted
335 odds of the shift leader reporting that there were enough staff for quality were 11%
336 lower, the odds of reporting nursing care left undone were increased by 14%, and
337 the odds of staff missing breaks were increased by 12%. Findings are similar for
338 shortfalls of nursing assistants.

339 Factors other than shortfalls relative to the SNCT estimated requirement were also
340 associated with perceptions of staffing adequacy. Nurses on surgical units were less
341 likely to perceive adequate staffing compared to nurses on other (medical or mixed)
342 units with lower odds of reporting enough staff for quality and higher odds or

For peer review only - http://bmjopen.bmj.com/site/about/guidelines.xhtml

reporting care left undone or missed breaks. For example, the odds of nurses reporting that there were enough staff for quality were 46% lower on surgical units. Although relationships were not significant and confidence intervals were wide, the odds of reporting enough staff for quality were substantially lower on units with a higher proportion of single rooms. Similarly, odds of reporting care left undone and missed breaks were substantially increased on units with a higher proportion of single rooms and units with higher turnover, although again confidence intervals were wide and relationships were not statistically significant. Nurses were more likely to report that there were enough staff for quality and less likely to report missed care and missed breaks on Saturday compared to Monday, although there was no consistent pattern that suggested weekends differed from weekdays overall.

Table 4 Association between staffing shortfall and nurse perceptions of staffing adequacy: univariable and multivariable models

	Enough staff for quality				Nursing care left undone				Staff breaks missed			
Variable	odds ratio*	adj odds ratio	95% confidence interval	p-value	odds ratio*	adj odds ratio	95% confidence interval	p-value	odds ratio*	adj odds ratio	95% confidence interval	p-value
Registered nurse shortfall (HPPD)	0.94	0.89	[0.87, 0.92]	0.000	1.09	1.14	[1.08, 1.20]	0.000	1.08	1.12	[1.06, 1.18]	0.000
Nursing assistant shortfall (HPPD)	0.90	0.86	[0.83, 0.89]	0.000	1.08	1.14	[1.07, 1.20]	0.000	1.06	1.11	[1.05, 1.17]	0.000
Turnover (per nursing hour)	0.35	0.91	[0.30, 2.75]	0.863	7.16	3.36	[0.60, 18.72]	0.167	3.85	4.94	[0.95, 25.78]	0.058
Unit type medical or mixed (ref)	1.00	1.00			1.00	1.00			1.00	1.00		
Surgical	0.57	0.54	[0.31, 0.92]	0.023	2.00	2.13	[1.19, 3.82]	0.011	2.13	2.15	[1.23, 3.78]	0.008
Proportion single rooms	0.89	0.54	[0.18, 1.66]	0.283	1.58	3.01	[0.93, 9.71]	0.065	1.05	2.06	[0.65, 6.56]	0.221
Day of week- Monday (ref)	1.00	1.00			1.00	1.00			1.00	1.00		
Tuesday	1.11	1.12	[0.99, 1.26]	0.079	0.86	0.86	[0.69, 1.06]	0.160	0.94	0.71	[0.58, 0.88]	0.001
Wednesday	1.28	1.28	[1.13, 1.45]	0.000	0.95	0.96	[0.78, 1.18]	0.684	1.01	0.61	[0.49, 0.76]	0.000
Thursday	1.09	1.08	[0.96, 1.23]	0.200	0.90	0.91	[0.73, 1.13]	0.383	0.96	0.81	[0.66, 1.00]	0.045
Friday	1.03	1.03	[0.91, 1.17]	0.610	0.93	0.93	[0.74, 1.15]	0.488	1.03	0.79	[0.64, 0.97]	0.028
Saturday	1.28	1.29	[1.14, 1.47]	0.000	0.74	0.75	[0.60, 0.95]	0.016	0.86	0.50	[0.40, 0.64]	0.000
Sunday	1.02	1.02	[0.90, 1.15]	0.811	1.08	1.11	[0.90, 1.37]	0.310	0.84	0.82	[0.66, 1.01]	0.058
Variance partition coefficient for units**		0.22				0.22				0.23		
Variance partition coefficient for hospitals**		0.12				0.17				0.11		
Akaike information criterion		20697				8377				8094		
Bayesian information criterion		20809				8490				8206		

Ref – reference category for categorical variables. *Odds ratio derived from entering this variable only into the multilevel model. Calculated as between-group residual variance divided by total variance using the latent variable approach [28]

For peer review only - http://bmjopen.bmj.com/site/about/guidelines.xhtml

We tested for non-linear relationships with registered nurse and nursing assistant shortfalls, as would be expected if the SNCT provides a threshold for adequate staffing. We estimated models using effects that were significant in the main effects models, adding a variable for staffing shortfall squared for each staff group. For nursing care left undone, the non-linear term for registered nurse staffing was significant but there was no clear indication that the model was preferable (AIC Δ-2, BIC Δ +14) and the overall relationship was not changed substantially. No indication of a threshold where benefit / harm starts could be observed (see Fig 2). For other outcomes, non-linear terms were not significant and associated with increased AIC & BIC (S1 Table 5).

FIG 2 Change in odds of reporting care left undone with change in staffing shortfalls estimated from model with non-linear staffing effects

We estimated models that included all statistically significant variables and interactions between staffing shortfall and these variables. There were no significant interaction effects between registered nurse shortfall and nursing assistant shortfall and both AIC and BIC increased for these models (S1 Table 6), so the simpler models were preferred.

Models using overall care hour shortfall (registered nurse and assistants) per patient day gave similar coefficients, with each care hour per patient day of shortfall associated with a 12% reduction in the odds of reporting enough staff for quality and no significant associations with skill mix (S1 Table 7). Because of the coding errors noted from units in one of the hospitals, we repeated the main models omitting data from this hospital. Results were largely unchanged with no effect on substantive conclusions (see S1 Table 8 for an example).

For peer review only - http://bmjopen.bmj.com/site/about/guidelines.xhtml

For peer review only

Discussion

This study reports the first independent assessment of the SNCT, which is widely used to determine staffing levels in English hospitals. Using the recommended minimum 20 day sample, estimates for the number of nurses that should be employed on a ward had an average precision of 4.2%, but wide confidence intervals for the absolute staff numbers needed. A sample of 40 days gave an estimate within +/- 1 staff members for the majority of wards but much larger samples (100 days or more) are required to estimate the staff required with a confidence interval width no more than 1 staff member wide in the majority of wards. When staffing shortfalls were high, relative to the required level estimated using the SNCT for that day, staff were less likely to report that they had enough staff for quality and more likely to report that necessary nursing care was omitted and staff breaks missed. These relationships appeared to be linear, with no threshold when staffing reached the SNCT recommended level. Other factors, not included in the patient classifications used by the SNCT, including unit speciality and day of the week are also associated with whether a given staffing level is deemed to be sufficient by nurses working on the unit.

The original purpose of the SNCT was to ensure that units employed sufficient nurses to be able to provide the care hours required by patients. Existing reports attest to the inter-rater reliability of the tool [16, 17] but the recommendation that this staffing establishment is estimated using at least 20 days of SNCT data recognises that daily demand is variable and estimates based on small samples may be imprecise. The average level of precision achieved from 20 days of observations in our study appears, superficially, to be acceptable. However, this masks considerable

For peer review only - http://bmjopen.bmj.com/site/about/guidelines.xhtml

variation in precision between units and large absolute differences in terms of numbers of staff. Using a conventional (if technically slightly inaccurate) interpretation of the confidence interval, this means that for many units, estimates could vary from the true staffing requirement by more than 2 whole staff members. The absolute importance of such differences may vary by unit, but the potential significance of such inaccuracy is great.

Small increases in the number of days used to estimate establishments yield substantial improvements in precision, although there are rapidly diminishing returns from samples of more than 40 days. As more and more hospitals are gathering SNCT ratings on a daily basis it may be that these data could be drawn on to review establishments, with the resources currently used to provide periodic review invested instead into quality control for the unit reports. Moving averages could be substituted for intermittent review, with statistical process control methods used to determine when changes in demand sufficient to revise the establishment have occurred [29].

For some units, variation is such that the estimated establishment may always be imprecisely estimated and our results also highlight that unmeasured influences on demand arising from factors such as unit layout and speciality could have a substantial influence on the staffing requirements. In these cases, the use of professional judgement, already emphasised within the guidance for the SNCT, is paramount. In the face of an apparently objective measure, it is easy to prioritise the measured quantity despite the substantial uncertainty associated with it. [30] It is clear from both these results and the wider literature that professional judgement remains an essential element in determining the required level of nurse staffing. [3, 4]

For peer review only - http://bmjopen.bmj.com/site/about/guidelines.xhtml

Although our results about other influences on workload were imprecise, both turnover and single rooms have been identified in other research as factors that increase nursing workload,[18-20, 31, 32] because of the specific work associated with admissions and discharge, increases in indirect care requirements and, for single rooms, the need for increased surveillance of potentially vulnerable patients. The increased workload associated with turnover is acknowledged in the SNCT, where revised multipliers are provided specific to acute admissions units, to reflect the high patient turnover.[15] Our findings could arise because variation in turnover within and between general units is not being sufficiently accommodated within the average demand across all patients. Our findings are consistent with surgical units having higher workload for a given level of acuity / dependency, which may also result from indirect care associated with surgery, such as arranging transports and providing escorts.[33] This is a novel finding, and where recommendations or mandates for minimum staffing levels exist, medical units are not generally differentiated from surgical units.[34]

While there must be a balance between parsimony and accuracy in any tool these findings could arise because the current SNCT staffing recommendations fit some units better than others. This lack of fit might be remedied by tailoring the tool and creating versions specific to particular circumstances. Further revisions to the SNCT recommended staffing levels, represented by different "multipliers" for different unit types beyond the current admissions unit specific multipliers, require further investigation. The wide confidence intervals associated with single rooms and turnover do not directly lend themselves to a formal revision of the multipliers, but the

For peer review only - http://bmjopen.bmj.com/site/about/guidelines.xhtml

importance of exercising professional judgments about other factors affecting workload is clear.

Although the SNCT multipliers were originally derived using professional expert estimates of time required, subsequent developments have used empirical observations to revise the multipliers.[17] Our study is the first to show that staffing shortfalls, relative to requirement estimated using the tool are associated with professional judgements that staffing is insufficient to maintain quality care and other indicators that staffing may be inadequate. However, if the SNCT were indicating a level of staffing that was generally judged sufficient to meet all care needs with quality, the relationship between shortfall and staffing adequacy would be expected to diminish as staffing levels increase above the recommended level. Instead the relationships we observed were essentially linear, with no evidence of a threshold above which additional staffing had little effect on the likelihood that nurses would report that there were enough staff. A recent study using the RAFAELA system, widely used in Northern Europe, gave a similar finding. Staffing above the level defined as 'optimal' by the system was associated with decreases in mortality[35, 36]. A recent study found that staffing below establishment as determined using the SNCT was associated with an increased risk of death in hospital [11, 20] but for registered nurses staffing the relationship was linear, with no threshold. So while our findings are consistent with the SNCT providing a measure of demand, there is no evidence to support the assertion that the recommended staffing levels are optimal in any meaningful sense.

The effects of registered nurse and nurse assistant shortfalls on perceptions of staffing adequacy were similar but independent. Although such a finding might be

For peer review only - http://bmjopen.bmj.com/site/about/guidelines.xhtml

interpreted as indicating that there is substitution between registered nurses and assistants, the contribution of the two groups to quality and safety is not equivalent they are not interchangeable. A large body of research points to the specific importance of maintaining a rich registered nurse skill mix for patient safety. [37] More recent studies have shown the important contribution of both registered nurses and assistants in maintaining both patient safety and the quality of interpersonal care. [11, 38, 39] Simple substitutions are not feasible because the contributions of each group are distinct. Effective deployment of assistants is contingent on having sufficient RNs to supervise and support them. [11, 38]

The SNCT, while widely used in England is by no means the only staffing tool available. Given the vast numbers of reports and different tools it is hard to say definitively that there are no data that would allow comparison of the precision of the SNCT with other tools for estimating staffing establishments, but our reviews found no recent studies giving similar data about other tools. [3, 4]

Limitations

Training was provided to unit nurses in using the SNCT but the extensive nature of the study is such that the reliability of the ratings we used is likely to be less than that achieved by expert raters in a dedicated review of establishments. However, the wide variation in precision of estimates of unit establishments is unlikely to be explained by this factor alone. Furthermore, the circumstances of our study resemble routine use of the system when, as is now becoming common, assessments are completed daily by shift leaders. We did find evidence of systematic coding errors for the assessment of staffing adequacy on some units, although our substantive conclusions were unaffected. However, this may indicate that less systematic errors

For peer review only - http://bmjopen.bmj.com/site/about/guidelines.xhtml

were also occurring. The effect of such errors would be to attenuate our ability to estimate relationships and so we may be underestimating the relationship between staffing shortfalls and perceptions of staffing adequacy. Our measures of staffing adequacy relied on subjective reports by nurses, but these subjective assessments have been shown to be associated with important patient outcomes [40-42]. Our sample was large but arose from only four hospitals so we cannot be sure that these results would generalise across all hospitals.

Conclusions

In this study we have asked (and answered) a number of questions about the SNCT. These questions: the precision with which establishments are estimated, the extent to which the averages provided from a patient classification can accommodate variation from factors that are unrelated to individual patients and whether the identified staffing level is in any sense “optimal”, all need to be asked of other tools. Our recent reviews suggest such questions are rarely asked of other tools and are even more rarely answered.

The SNCT can provide a reliable estimate of a unit staffing establishment but larger samples than the currently recommended minimum are required for most units to provide estimates that are within 1 whole time equivalent staff member of the mean. For some units, such precision is hard to obtain and there may be systematic variations in staffing requirements associated with some unit types that are not accounted for by the SNCT. While we recommend further exploration of the factors affecting the reliability and validity of the SNCT estimates, and suggest that moving averages instead of periodic reassessments could be used to identify when changes in establishments are needed, our findings also firmly underpin the conclusion that

For peer review only - http://bmjopen.bmj.com/site/about/guidelines.xhtml

measurement is an adjunct to professional judgements, not a replacement for it. The SNCT does appear to provide a measure of nursing workload but the recommended staffing levels derived from it are not necessarily optimal.

[words 5254]

For peer review only

For peer review only - http://bmjopen.bmj.com/site/about/guidelines.xhtml

Acknowledgements

The authors of this paper wish to acknowledge the contributions of Rosemary Chable, Andrew Dimech, Jeremy Jones, Yvonne Jeffrey, Antonello Maruotti, Alejandra Recio Saucedo, and Nicola Sinden who contributed to the acquisition of funding and the design of the project; and Clare Aspden, Tracey Cassar and Shirley Hunter who contributed to data collection. We also wish to acknowledge all the nurses who answered staffing adequacy questions and completed SNCT ratings. This paper draws on research and data to be reported in more detail in the NIHR Journals Library Health Services and Delivery Journal.

Author statement

Peter Griffiths (Professor, Health Services Research) Principal Investigator, original conceptualisation of the study and study design, secured funding, oversaw acquisition of the data, data analysis, interpretation of results and drafted article.
Christina Saville (Research Fellow, Operational Research) undertook descriptive and regression analyses and contributed to critical revision of the article.
Jane Ball (Professor, Health Services Research) contributed to study design, acquisition of funding, interpretation of results and critical revision of the article.
David Culliford (Senior Medical Statistician) provided statistical advice, contributed to interpretation of results and critical revision of the article.
Natalie Pattison (Clinical Professor, Nursing) contributed to study design, acquisition of funding, acquisition of the data, interpretation of results and critical revision of the article.
Thomas Monks (Principal Research Fellow, Operational Research and Data Science) contributed to study design, acquisition of funding, oversaw acquisition of the data, data analysis, interpretation of results and critical revision of the article.

Funding statement

This report presents independent research funded by the United Kingdom's National Institute for Health Research (NIHR) Health Services and Delivery Research Programme number 14/194/21. JEB, NP, PG and TM were award holders. The funders had no role in study design, data collection and analysis, decision to publish, or preparation of the manuscript. The views and opinions expressed by authors in

For peer review only - http://bmjopen.bmj.com/site/about/guidelines.xhtml

this publication are those of the authors and do not necessarily reflect those of the NHS, the NIHR, NETSCC, the PHR programme or the Department of Health and Social Care.

Competing interests

Peter Griffiths is a member of the National Health Service Improvement (NHSI) safe staffing faculty steering group. The safe staffing faculty programme is intended to ensure that knowledge of the Safer Nursing Care Tool (SNCT), its development and its operational application is consistently applied across the NHS.

Data sharing statement

All data supporting this article are openly available from the University of Southampton repository at https://doi.org/10.5258/SOTON/ D1134.

For peer review only

For peer review only - http://bmjopen.bmj.com/site/about/guidelines.xhtml

References

1. Kavanagh KT, Cimiotti JP, Abusalem S, et al. Moving healthcare quality forward with nursing-sensitive value-based purchasing. *J Nurs Scholarsh* 2012; 44: 385-395. 2012/10/17. DOI: 10.1111/j.1547-5069.2012.01469.x.
2. Griffiths P, Ball J, Drennan J, et al. Nurse staffing and patient outcomes: Strengths and limitations of the evidence to inform policy and practice. A review and discussion paper based on evidence reviewed for the National Institute for Health and Care Excellence Safe Staffing guideline development. *Int J Nurs Stud* 2016; 63: 213-225. 2016/05/01. DOI: 10.1016/j.ijnurstu.2016.03.012.
3. Saville CE, Griffiths P, Ball JE, et al. How many nurses do we need? A review and discussion of operational research techniques applied to nurse staffing. *Int J Nurs Stud* 2019; 97: 7-13. 2019/05/28. DOI: 10.1016/j.ijnurstu.2019.04.015.
4. Griffiths P, Saville C, Ball J, et al. Nursing workload, Nurse Staffing Methodologies & Tools: a systematic scoping review & discussion. *Int J Nurs Stud* 2019 (in press).
5. Ball J, Barker H, Griffiths P, et al. *Implementation, Impact and Costs of Policies for Safe Staffing In Acute Trusts: Report to Funders:*. 2019. Southampton: University of Southampton.
6. National Institute for Health and Clinical Excellence. Safer Nursing Care Tool (SNCT): statement of endorsement, https://www.nice.org.uk/guidance/sg1/resources/endorsed-resource-the-safer-nursing-care-tool-snct-11943902 (2014, accessed 29 October 2018).
7. Griffiths P, Recio-Saucedo A, Dall'Ora C, et al. The association between nurse staffing and omissions in nursing care: A systematic review. *J Adv Nurs* 2018; 74: 1474-1487. 2018/03/09. DOI: 10.1111/jan.13564.
8. Kane RL, Shamliyan TA, Mueller C, et al. The association of registered nurse staffing levels and patient outcomes: systematic review and meta-analysis. *Med Care* 2007; 45: 1195-1204. 2007/11/17. DOI: 10.1097/MLR.0b013e3181468ca3.
9. Shekelle PG. Nurse-patient ratios as a patient safety strategy: a systematic review. *Ann Intern Med* 2013; 158: 404-409. 2013/03/06. DOI: 10.7326/0003-4819-158-5-201303051-00007.
10. Aiken LH, Sloane DM, Bruyneel L, et al. Nurse staffing and education and hospital mortality in nine European countries: a retrospective observational study. *Lancet* 2014; 383: 1824-1830. 2014/03/04. DOI: 10.1016/S0140-6736(13)62631-8.
11. Griffiths P, Ball J, Bloor K, et al. Nurse staffing levels, missed vital signs and mortality in hospitals: retrospective longitudinal observational study. *Health Services and Delivery Research Journal* 2018; 6: 38. DOI: 10.3310/hsdr06380.
12. Ball JE, Murrells T, Rafferty AM, et al. 'Care left undone' during nursing shifts: associations with workload and perceived quality of care. *BMJ Qual Saf* 2014; 23: 116-125. 2013/07/31. DOI: 10.1136/bmjqs-2012-001767.
13. Hoi SY, Ismail N, Ong LC, et al. Determining nurse staffing needs: the workload intensity measurement system. *J Nurs Manag* 2010; 18: 44-53. 2010/05/15. DOI: 10.1111/j.1365-2834.2009.01045.x.

For peer review only - http://bmjopen.bmj.com/site/about/guidelines.xhtml

14. Buchan J. A certain ratio? The policy implications of minimum staffing ratios in nursing. *J Health Serv Res Policy* 2005; 10: 239-244. 2005/11/02. DOI: 10.1258/135581905774414204.
15. The Shelford Group. *Safer Nursing Care Tool Implementation Resource Pack*. 2014. The Shelford Group.
16. Hurst K, Smith A, Casey A, et al. Calculating staffing requirements. *Nurs Manag (Harrow)* 2008; 15: 26-34. 2008/08/02. DOI: 10.7748/nm2008.07.15.4.26.c6616.
17. Smith S, Casey A, Hurst K, et al. Developing, testing and applying instruments for measuring rising dependency-acuity's impact on ward staffing and quality. *Int J Health Care Qual Assur* 2009; 22: 30-39. 2009/03/17. DOI: 10.1108/09526860910927934.
18. Hurst K. UK ward design: patient dependency, nursing workload, staffing and quality-an observational study. *Int J Nurs Stud* 2008; 45: 370-381. doi: DOI: 10.1016/j.ijnurstu.2006.09.007 2006/11/14. DOI: 10.1016/j.ijnurstu.2006.09.007.
19. Maben J, Griffiths P, Penfold C, et al. One size fits all? Mixed methods evaluation of the impact of 100% single-room accommodation on staff and patient experience, safety and costs. *BMJ Qual Saf* 2016; 25: 241-256. 2015/09/27. DOI: 10.1136/bmjqs-2015-004265.
20. Griffiths P, Maruotti A, Recio Saucedo A, et al. Nurse staffing, nursing assistants and hospital mortality: retrospective longitudinal cohort study. *BMJ Qual Saf* 2019; 28: 609-617. 2018/12/06. DOI: 10.1136/bmjqs-2018-008043.
21. Wood VJ, Vindrola-Padros C, Swart N, et al. One to one specialling and sitters in acute care hospitals: A scoping review. *Int J Nurs Stud* 2018; 84: 61-77. 2018/05/18. DOI: 10.1016/j.ijnurstu.2018.04.018.
22. Sermeus W, Aiken LH, Van den Heede K, et al. Nurse forecasting in Europe (RN4CAST): Rationale, design and methodology. *BMC Nurs* 2011; 10: 6. 2011/04/20. DOI: 10.1186/1472-6955-10-6.
23. Patterson J. The effects of nurse to patient ratios. *Nurs Times* 2011; 107: 22-25. 2011/03/04.
24. R Core Team. *R: A Language and Environment for Statistical Computing*. v3.5.0 ed. Vienna, Austria: R Foundation for Statistical Computing, 2019.
25. Bates D, Mächler M, Bolker B, et al. Fitting Linear Mixed-Effects Models Usinglme4. *Journal of Statistical Software* 2015; 67: 1-48. sparse matrix methods; linear mixed models; penalized least squares; Cholesky decomposition 2015-10-07. DOI: 10.18637/jss.v067.i01.
26. Burnham KP and Anderson DR. Multimodel Inference. *Sociological Methods & Research* 2016; 33: 261-304. DOI: 10.1177/0049124104268644.
27. Griffiths P. Using the Safer Nursing Care Tool to identify nurse staffing requirements in hospitals. *ISRTCN* 2016; https://doi.org/10.1186/ISRCTN12307968.
28. Goldstein H, Browne W and Rasbash J. Partitioning Variation in Multilevel Models. *Understanding Statistics* 2002; 1: 223-231. DOI: 10.1207/S15328031US0104_02.
29. Wong H, Gan F and Chang T. Designs of moving average control chart. *Journal of Statistical Computation and Simulation* 2004; 74: 47-62.

For peer review only - http://bmjopen.bmj.com/site/about/guidelines.xhtml

30. Downie R and Macnaughton J. In Defence of Professional Judgement. *Adv Psychiatr Treat* 2013; 15: 322-327. 2013/10/01. DOI: 10.1192/apt.bp.108.005926.
31. Needleman J, Buerhaus P, Pankratz VS, et al. Nurse staffing and inpatient hospital mortality. *N Engl J Med* 2011; 364: 1037-1045. 2011/03/18. DOI: 10.1056/NEJMsa1001025.
32. Myny D, Van Hecke A, De Bacquer D, et al. Determining a set of measurable and relevant factors affecting nursing workload in the acute care hospital setting: a cross-sectional study. *Int J Nurs Stud* 2012; 49: 427-436. 2011/10/28. DOI: 10.1016/j.ijnurstu.2011.10.005.
33. Myny D, Van Goubergen D, Gobert M, et al. Non-direct patient care factors influencing nursing workload: a review of the literature. *J Adv Nurs* 2011; 67: 2109-2129. 2011/07/05. DOI: 10.1111/j.1365-2648.2011.05689.x.
34. Royal College of Nursing. *Policy Briefing: Mandatory Nurse Staffing levels*. 2012. London: RCN.
35. Fagerstrom L, Kinnunen M and Saarela J. Nursing workload, patient safety incidents and mortality: an observational study from Finland. *BMJ Open* 2018; 8: e016367. 2018/04/25. DOI: 10.1136/bmjopen-2017-016367.
36. Junttila JK, Koivu A, Fagerstrom L, et al. Hospital mortality and optimality of nursing workload: A study on the predictive validity of the RAFAELA Nursing Intensity and Staffing system. *Int J Nurs Stud* 2016; 60: 46-53. 2016/06/15. DOI: 10.1016/j.ijnurstu.2016.03.008.
37. Aiken LH, Sloane D, Griffiths P, et al. Nursing skill mix in European hospitals: cross-sectional study of the association with mortality, patient ratings, and quality of care. *BMJ Qual Saf* 2017; 26: 559-568. 2017/06/20. DOI: 10.1136/bmjqs-2016-005567.
38. Bridges J, Griffiths P, Oliver E, et al. Hospital nurse staffing and staff-patient interactions: an observational study. *BMJ Qual Saf* 2019; 28: 706-713. 2019/03/29. DOI: 10.1136/bmjqs-2018-008948.
39. Shang J, Needleman J, Liu J, et al. Nurse Staffing and Healthcare-Associated Infection, Unit-Level Analysis. *J Nurs Adm* 2019; 49: 260-265. 2019/04/23. DOI: 10.1097/NNA.0000000000000748.
40. Ball JE, Bruyneel L, Aiken LH, et al. Post-operative mortality, missed care and nurse staffing in nine countries: A cross-sectional study. *Int J Nurs Stud* 2018; 78: 10-15. 2017/08/29. DOI: 10.1016/j.ijnurstu.2017.08.004.
41. Recio-Saucedo A, Dall'Ora C, Maruotti A, et al. What impact does nursing care left undone have on patient outcomes? Review of the literature. *J Clin Nurs* 2018; 27: 2248-2259. 2017/09/01. DOI: 10.1111/jocn.14058.
42. Bruyneel L, Li B, Ausserhofer D, et al. Organization of Hospital Nursing, Provision of Nursing Care, and Patient Experiences With Care in Europe. *Med Care Res Rev* 2015; 72: 643-664. 2015/06/13. DOI: 10.1177/1077558715589188.

For peer review only - http://bmjopen.bmj.com/site/about/guidelines.xhtml

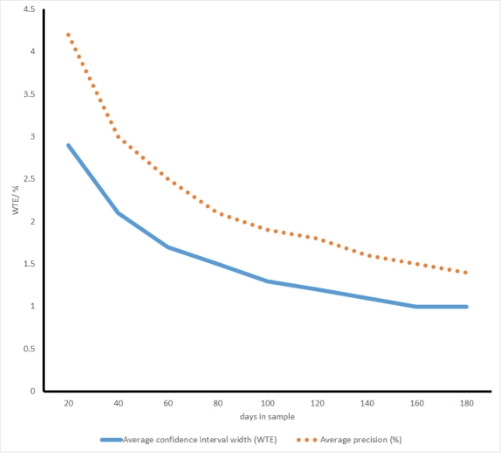

Mean precision and confidence interval width of staffing establishment estimates with different sample sizes

146x82mm (300 x 300 DPI)

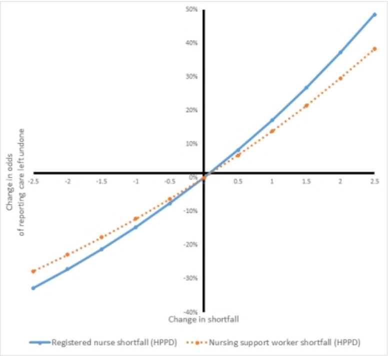

Change in odds of reporting care left undone with change in staffing shortfalls estimated from model with non-linear staffing effects

304x171mm (144 x 144 DPI)

Supplemental material

Table 1 Association between staffing shortfall and nurse perceptions of staffing adequacy: test for non-linear staffing effects in multivariable models.

	ENOUGH STAFF FOR QUALITY			NURSING CARE LEFT UNDONE			STAFF BREAKS MISSED		
**VARIABLE**	Odds ratio	95% confidence interval	p-value	Odds ratio	95% confidence interval	p-value	Odds ratio	95% confidence interval	p-value
**REGISTERED NURSE SHORTFALL (HPPD)**	0.89	[0.86, 0.92]	**<0.001**	1.15	[1.09, 1.21]	**<0.001**	1.13	[1.08, 1.19]	**<0.001**
**NURSING SUPPORT WORKER SHORTFALL (HPPD)**	0.85	[0.83, 0.88]	**<0.001**	1.15	[1.08, 1.22]	**<0.001**	1.12	[1.06, 1.19]	**<0.001**
**REGISTERED NURSE SHORTFALL SQUARED**	1.00	[0.99, 1.00]	0.469	1.01	[1.00, 1.02]	**0.021**	1.00	[0.99, 1.02]	0.533
**NURSING SUPPORT WORKER SHORTFALL SQUARED**	1.01	[1.00, 1.02]	0.102	0.99	[0.97, 1.01]	0.194	0.99	[0.97, 1.01]	0.165
**DAY OF WEEK- MONDAY (REFERENCE)**									
**TUESDAY**							0.71	[0.58, 0.88]	**0.002**
**WEDNESDAY**							0.61	[0.49, 0.75]	**<0.001**
**THURSDAY**							0.82	[0.66, 1.00]	0.053
**FRIDAY**							0.80	[0.65, 0.99]	**0.042**
**SATURDAY**							0.49	[0.39, 0.62]	**<0.001**
**SUNDAY**							0.80	[0.65, 0.98]	**0.031**
**WARD TYPE- OTHER (REFERENCE)**									
**SURGICAL**	0.57	[0.33, 0.96]	**0.036**	1.98	[1.10, 3.54]	**0.022**	2.07	[1.18, 3.63]	**0.011**
**PROPORTION SINGLE ROOMS**									
**VARIANCE PARTITION COEFFICIENT FOR UNITS**	0.22			0.21			0.23		
**VARIANCE PARTITION COEFFICIENT FOR HOSPITALS**	0.15			0.20			0.13		
**AKAIKE INFORMATION CRITERION**	20715			8378			8097		
**BAYESIAN INFORMATION CRITERION**	20779			8442			8209		

For peer review only

For peer review only - http://bmjopen.bmj.com/site/about/guidelines.xhtml

Table 2 Association between staffing shortfall and nurse perceptions of staffing adequacy: test for interactions between significant variables in multivariable models

	Enough staff for quality			Nursing care left undone			Staff breaks Missed		
**Variable**	**Odds ratio**	**95% confidence interval**	**p-value**	**Odds ratio**	**95% confidence interval**	**p-value**	**Odds ratio**	**95% confidence interval**	**p-value**
**Registered nurse shortfall (HPPD)**	0.92	[0.89, 0.95]	**<0.001**	1.14	[1.07, 1.21]	**<0.001**	1.13	[1.02, 1.25]	**0.019**
**Nursing support worker shortfall (HPPD)**	0.87	[0.84, 0.90]	**<0.001**	1.14	[1.06, 1.22]	**<0.001**	1.13	[1.00, 1.28]	**0.049**
**Registered nurse shortfall squared (HPPD)**				1.01	[1.00, 1.02]	0.044			
**Day of week- Monday (reference)**									
**Tuesday**							0.71	[0.57, 0.90]	**0.005**
**Wednesday**							0.60	[0.47, 0.76]	**<0.001**
**Thursday**							0.84	[0.67, 1.05]	0.135
**Friday**							0.87	[0.69, 1.09]	0.225
**Saturday**							0.50	[0.38, 0.65]	**<0.001**
**Sunday**							0.81	[0.64, 1.01]	0.064
**Unit type- Other (reference)**									
**Surgical**	0.63	[0.37, 1.08]	0.096	2.03	[1.11, 3.69]	**0.021**	2.04	[1.15, 3.63]	**0.015**
**Proportion single rooms**									
**Turnover (per nursing hour)**									
**Registered nurse shortfall : Nursing support worker shortfall**	0.99	[0.98, 1.01]	0.315	1.00	[0.98, 1.02]	0.972	1.01	[0.99, 1.03]	0.202
**Registered nurse shortfall : Tuesday**							0.98	[0.86, 1.11]	0.708
**Registered nurse shortfall : Wednesday**							0.99	[0.86, 1.13]	0.840
**Registered nurse shortfall : Thursday**							0.96	[0.84, 1.09]	0.540
**Registered nurse shortfall : Friday**							0.94	[0.82, 1.07]	0.332
**Registered nurse shortfall : Saturday**							1.05	[0.89, 1.24]	0.558
**Registered nurse shortfall : Sunday**							1.04	[0.91, 1.20]	0.566
**Registered nurse shortfall : Surgical unit**	0.88	[0.82, 0.95]	**0.001**	1.00	[0.89, 1.13]	0.952	1.00	[0.89, 1.12]	0.961
**Registered nurse shortfall: Proportion single rooms**									

For peer review only - http://bmjopen.bmj.com/site/about/guidelines.xhtml

	Enough staff for quality			Nursing care left undone			Staff breaks Missed		
**Variable**	**Odds ratio**	**95% confidence interval**	**p-value**	**Odds ratio**	**95% confidence interval**	**p-value**	**Odds ratio**	**95% confidence interval**	**p-value**
**Nursing support worker shortfall: Tuesday**							1.01	[0.85, 1.20]	0.905
**Nursing support worker shortfall: Wed**							1.04	[0.87, 1.24]	0.694
**Nursing support worker shortfall: Thursday**							0.95	[0.81, 1.11]	0.536
**Nursing support worker shortfall: Friday**							0.87	[0.74, 1.02]	0.097
**Nursing support worker shortfall: Saturday**							0.92	[0.76, 1.11]	0.390
**Nursing support worker shortfall: Sunday**							0.95	[0.80, 1.12]	0.522
**Nursing support worker shortfall: Surgical unit**	0.95	[0.88, 1.04]	0.263	0.98	[0.86, 1.11]	0.766	1.07	[0.94, 1.21]	0.325
**Nursing support worker shortfall: Proportion single rooms**									
Variance partition coefficient for units	0.22			0.21			0.23		
Variance partition coefficient for hospitals	0.14			0.20			0.13		
**Akaike information criterion**	20708			8384			8116		
**Bayesian information criterion**	20780			8464			8341		

For peer review only - http://bmjopen.bmj.com/site/about/guidelines.xhtml

Table 3: Association between overall (registered nurse and assistant) shortfall and nurse perceptions of staffing adequacy

	**Enough staff for quality**			**Nursing care left undone**			**Staff breaks missed**		
**Variable**	Odds ratio	95% confidence interval	p-value	Odds ratio	95% confidence interval	p-value	Odds ratio	95% confidence interval	p-value
**Overall shortfall (HPPD)**	0.88	[0.86, 0.90]	**<0.001**	1.14	[1.09, 1.19]	**<0.001**	1.11	[1.07, 1.16]	**<0.001**
**Skill mix (registered nurse HPPD/ total HPPD)**	0.81	[0.62, 1.06]	0.117	0.74	[0.47, 1.17]	0.195	0.88	[0.57, 1.38]	0.586
**Patient turnover (per care hour)**	0.91	[0.30, 2.77]	0.869	3.51	[0.63, 19.65]	0.153	4.99	[0.96, 26.02]	0.057
**Unit type- other (reference)**									
**Surgical**	0.54	[0.31, 0.93]	**0.025**	2.14	[1.20, 3.83]	**0.010**	2.16	[1.23, 3.80]	**0.007**
**Proportion single rooms**	0.53	[0.17, 1.64]	0.271	3.01	[0.93, 9.69]	0.065	2.06	[0.65, 6.57]	0.222
**Day of week- Monday (reference)**									
**Tuesday**	1.12	[0.99, 1.26]	0.078	0.86	[0.69, 1.06]	0.159	0.71	[0.58, 0.88]	**0.001**
**Wednesday**	1.28	[1.13, 1.45]	**<0.001**	0.96	[0.77, 1.18]	0.676	0.61	[0.49, 0.76]	**<0.001**
**Thursday**	1.09	[0.96, 1.23]	0.195	0.91	[0.73, 1.13]	0.379	0.81	[0.66, 1.00]	**0.045**
**Friday**	1.03	[0.91, 1.17]	0.604	0.92	[0.74, 1.15]	0.481	0.79	[0.64, 0.97]	**0.028**
**Saturday**	1.30	[1.14, 1.48]	**<0.001**	0.75	[0.60, 0.95]	0.016	0.50	[0.40, 0.64]	**<0.001**
**Sunday**	1.02	[0.90, 1.15]	0.795	1.11	[0.90, 1.37]	0.316	0.82	[0.66, 1.01]	0.058
Variance partition coefficient for units	0.22			0.22			0.23		
Variance partition coefficient for hospitals	0.12			0.17			0.11		
**Akaike information criterion**	20700			8376			8094		
**Bayesian information criterion**	20812			8488			8206		

For peer review only

Table 4: Association between staffing shortfall and nurse perceptions of "enough staff for quality": comparison of coefficients from models with full sample vs model excluding the Trust where recoding occurred.

	**Model with full sample**			**MODEL EXCLUDING 'HOSPITAL D'**		
**Variable**	ADJ ODDS RATIO	95% CONFIDENCE INTERVAL	P-VALUE	ADJ ODDS RATIO	95% CONFIDENCE INTERVAL	P-VALUE
**Registered nurse shortfall (HPPD)**	0.89	[0.87, 0.92]	**<0.001**	0.89	[0.86, 0.91]	**<0.001**
**Nursing support worker shortfall (HPPD)**	0.86	[0.83, 0.89]	**<0.001**	0.84	[0.81, 0.87]	**<0.001**
**Turnover (per care hour)**	0.91	[0.30, 2.75]	0.863	0.83	[0.26, 2.63]	0.747
**Ward type – other (reference)**						
**Surgical**	0.54	[0.31, 0.92]	**0.023**	0.45	[0.27, 0.76]	**0.003**
**Proportion single rooms**	0.54	[0.18, 1.66]	0.283	0.61	[0.24, 1.57]	0.304
**Day of week- Monday (reference)**						
**Tuesday**	1.12	[0.99, 1.26]	0.079	1.20	[1.05, 1.37]	**0.008**
**Wednesday**	1.28	[1.13, 1.45]	**<0.001**	1.31	[1.15, 1.50]	**<0.001**
**Thursday**	1.08	[0.96, 1.23]	0.2000	1.13	[0.99, 1.29]	**0.071**
**Friday**	1.03	[0.91, 1.17]	0.610	1.08	[0.94, 1.24]	0.278
**Saturday**	1.29	[1.14, 1.47]	**<0.001**	1.40	[1.21, 1.61]	**<0.001**
**Sunday**	1.02	[0.90, 1.15]	0.811	1.03	[0.90, 1.17]	0.697

For peer review only

For peer review only - http://bmjopen.bmj.com/site/about/guidelines.xhtml

Identifying nurse-staffing requirements using the Safer Nursing Care Tool. Modelling the costs and consequences of real world application to address variation in patient need on hospital ward

Protocol HS&DR 14/194/21

IRAS project ID is 190548

For peer review only

Identifying nurse-staffing requirements using the Safer Nursing Care Tool.

Version Number	Author	Effective Date	Reason for Change
1.0	PG	Jan 13 2016	Protocol as submitted for final funding approval with minor edits to correct typo, layout and removing content not required in a protocol
	Last opened	18/11/2019 05:58:10	
1.1	PG	1.11.2016	Changes to timings of Questions; additional Staffing Adequacy questions; Staffing Adequacy collection times
Original version and subsequent amendments produced by the study team © University of Southampton 2016			

For peer review only

Study team

University of Southampton

Peter Griffiths

Jane Ball

Tom Monks

Alejandro Recio Saucedo

NIHR CLAHRC Wessex, University of Southampton

Jeremy Jones

Antonello Maruotti

Poole Hospital NHS Foundation Trust

Yvonne Jeffrey

Portsmouth NHS Trust

Nicky Sinden

Royal Marsden HHS Foundation Trust

Andrew Dimech

Natalie Pattison

University Hospital Southampton NHS Trust

Rosemary Chable

Claire Aspden

For peer review only - http://bmjopen.bmj.com/site/about/guidelines.xhtml

Identifying nurse-staffing requirements using the Safer Nursing Care Tool.

For peer review only

For peer review only - http://bmjopen.bmj.com/site/about/guidelines.xhtml

Identifying nurse-staffing requirements using the Safer Nursing Care Tool.

Summary of Research

The quality of nursing care and the potential for inadequate nursing to harm patients has emerged as a factor in many reports on failings in NHS hospitals. [1, 2] Reports often cite inadequate nurse staffing as a causal factor in these failures. NICE has recently issued guidance on determining safe staffing for hospital wards[3] which endorsed using the Safer Nursing Care Tool (SNCT) to indicate the number of nurses required on a ward to meet patient need, based on a patient acuity/dependency measure. [4, 5]

Our review for NICE found little evidence about the costs or consequences of tools used to determine staffing levels based on assessed patient need, or the extent to which different staffing policies, based on using the tools are affordable, effective or feasible. [6] Our proposed study examines how patients' needs for nursing care, as measured by the SNCT, vary from day to day. This will allow us to determine how often staffing shortfalls occur, whether there may be excess staff on other wards who could make up a shortfall, and to model the costs and consequences of different strategies for using the tool and deploying nursing staff to meet varying need.

We will collect data on ward nurse staffing, validated nurse reported measures of staffing adequacy, and SNCT measures of patient acuity/dependency from all adult medical / surgical wards of three general and one specialist hospitals daily over a period of 1 year. We will compare daily nursing hours (RN, HCA, all nurses) available per patient to:

- the required nursing hours (derived from daily SNCT acuity/ dependency assessment)
- planned staffing for that day and
- professional judgment of staffing adequacy.

We will assess the association between periods of understaffing identified by the tool and nurse reported staffing adequacy and use computer modelling techniques that account for variability in patient acuity, length of stay, admissions and workforce availability, to compare the recommended strategy (staffing based on mean patient acuity/dependency) with:

- a maximum staffing strategy (staffing set to meet maximum patient acuity dependency)
- a flexible staffing strategy (regular staffing set to meet minimum patient acuity/dependency with shortfall met by redeployment from other wards or bank/agency staffing)
- other staffing policies, as determined by an expert reference group.

For each strategy, we will assess the proportion of shifts with a critical shortfall of nurses using criteria derived from NICE guidance. We will model the staffing costs and consequences of each strategy with varying approaches to filling critical shortfalls. Costs will include costs of any ward nursing staff and bank / agency staff deployed to meet shortfalls. Consequences will be modelled using regression coefficients from robust studies showing the association between nurse staffing and outcomes. Feasibility of flexible strategies will be assessed by examining data to identify when staffing shortfalls can be met by surplus on other wards and the required availability of bank/agency staff.

The study will provide evidence for the usefulness and accuracy of the SNCT, which is widely used in the NHS. The results of the study will give guidance on the feasibility and relative costs and consequences of a variety of staffing policies aimed at addressing fluctuations in demand, including flexible staffing. To our knowledge this will be the first substantial study conducted using the SNCT other than those by its developers and the only study to have modelled the costs and consequences of using it to guide flexible staffing policies.

For peer review only - http://bmjopen.bmj.com/site/about/guidelines.xhtml

Identifying nurse-staffing requirements using the Safer Nursing Care Tool.

Background and Rationale

What is the problem being addressed?

NICE guidance "safe staffing for nursing in adult inpatient wards in acute hospitals" (SG 1) recommends a systematic approach to determining the staffing requirements of hospital wards. [3] The recommended approach to setting a staffing establishment, that is the number of nurses employed in order to meet patient needs 24 hours per day, seven days per week, is based on the use of an endorsed toolkit to assess average patient needs on a particular ward. The only toolkit currently endorsed by NICE is the Safer Nursing Care Tool (SNCT). [4, 5] This tool is widely used within the NHS. [7] In our proposed research we aim to determine the feasibility, likely costs and consequences of using the SNCT to setting safe nurse staff levels.

Using the SNCT to establish staffing requirements, the acuity and dependency of every patient on the ward is assessed over a baseline period. The tool provides 'multipliers' to translate patient acuity and dependency (assigned to one of 5 categories) into a staffing requirement.[4, 5] The ward establishment is then set, based upon staff required to meet the average care requirements, with allowances for sick leave, holidays and study leave. However, we do not know whether this approach gives an efficient or effective solution to ward staffing, given fluctuations in need. It is unclear how often the average staffing levels match daily requirements or how often wards are over or under staffed when these averages are used to plan staffing. International studies indicate considerable daily variation in workload intensity for nurses. [8] Modelling studies suggest that staffing based on average requirements can lead to critical shortfalls in the face of variable need. [9] and empirical evidence suggests that substantial mismatches between workload and available staff are common [8] even where formal staffing methodologies are in use. [10] However, we have no equivalent data from the UK.

In 2010, as part of the RN4CAST study of nursing workforce, we undertook a survey of a stratified random sample of 31 English acute hospital Trusts. We found that a majority (59%) of these Trusts used a formal acuity/dependency system to guide nurse-staffing decisions. Most identified the tool used as the SNCT or its precursor. [7] Although the SNCT is designed to set nursing establishments based on periodic review, thirty-six percent of Trusts reported that they monitor acuity/dependency daily, making a responsive, flexible staffing policy potentially feasible. In this study we will assess a range of options for using the SNCT and model the costs and consequences of various ward staffing policies based on dependency/acuity assessments derived from it.

Why is this important?

The Francis inquiries and the Keogh review into care provided by 14 hospital trusts with persistently high mortality rates identified inadequate nurse staffing as a significant factor associated with poor patient outcomes, including death. [1, 2] These reports are consistent with a large body of evidence linking low nurse staffing level to higher hospital mortality rates. [6, 11] Recently, our RN4CAST study in 300 hospitals across nine European countries showed a 7% increase in the odds of death among surgical patients for every additional patient per registered nurse. [12] However, beyond emphasising the vital importance of nurse staffing levels, these studies do little to help determine the correct level of staffing, as we identified in the evidence we reviewed for the NICE safe nurse staffing committee. [3] More usefully, Needleman's recent study identified that risk of death for a hospital patient was

For peer review only - http://bmjopen.bmj.com/site/about/guidelines.xhtml

significantly increased by exposure to nursing shifts where staffing fell below what was planned (hazard ratio 1.02 per shift of 8 hours or more below target staffing). This identified the crucial importance of methods to plan staffing and meet fluctuation in demand.

NICE recommended a systematic approach at ward level to ensure that patients receive the nursing care they need, regardless of the ward to which they are allocated, the time of the day, or the day of the week. [3] In response to the NICE guidance, NHS England has mandated the publication of data regarding the proportion of daily planned nursing hours that are actually filled. The published data shows substantial variation between Trusts (http://www.nhs.uk/). However, despite outlining guiding principles of staff planning in the NQB/NHS England document '"How to ensure the right people, with the right skills, are in the right place at the right time"', [13] neither NHS England nor Needleman's study is explicit about how planned staffing should be arrived at, in terms of the recommended "evidence based" tools, or how they should be used.

If deviation between planned staffing and that deployed is to be a useful indicator of safety, more evidence is required about the systematic approaches to be taken to determine staffing requirements in the first place. Furthermore, evidence is needed to understand how best to plan staffing to meet variations in patient need between wards and over time. NICE is assessing and endorsing tools that are broadly compliant with its guidance. To date only one tool, the Safer Nursing Care Tool, [4] has been endorsed by NICE. This tool is the most widely used in the NHS.[7]

The NHS faces pressure to maintain the quality and safety of care in hospitals at the same or less cost than previously. However, there remains a dearth of evidence on the effectiveness of tools, including the SNCT, or how best to translate estimates of patients' average requirements for nursing care into daily staffing plans. [14, 15] While the published evidence on the SNCT shows variation in average patient need from one quarter to another, emphasising the need for periodic review of establishments, [5] we are aware of no published data indicating the extent of variation from day to day as measured by the tool. Such information is necessary in order to understand the implications of planning staffing based on average requirements and the feasibility of flexible responses to daily variation. [9] We have undertaken small scale pilot work in one Trust, that found that the required daily nursing hours measured by the SNCT varied by approximately +/- 30% (SD 15%) from the mean, whilst the number of staff deployed varied much less (RN staffing approx. +/-10%). There was little correlation (r<-0.01 to 0.16) between staffing deployed and the SNCT measure (unpublished doctoral work).

Although the SNCT guidance focusses on setting ward establishments (that is the number of nurses employed) 36% of Trusts that reported using it in in our RN4CAST survey monitor acuity and dependency daily, making a responsive / flexible staffing policy using the tool possible. But the feasibility, costs and consequences of different approaches to responding to varying patient needs are unknown.

For peer review only - http://bmjopen.bmj.com/site/about/guidelines.xhtml

Identifying nurse-staffing requirements using the Safer Nursing Care Tool.

Why is it needed now?

In our review of evidence to inform the NICE safe staffing guidance for nursing in adult inpatient wards we found no evidence for the effectiveness of any tools or systems designed to guide decisions about staffing levels on hospital wards. [6] There are many such systems. A review undertaken for the then DHSS in 1982 identified over 400. [16] A more recent review of 58 studies concluded that there is little objective and validated information regarding systems to determine staffing requirements, and that current evidence provides insufficient accuracy for resource allocation or for decision making.[15] The field is dominated by descriptive reports of locally developed studies with limited or weak evidence for validity even for widely used and commercially supported approaches. [14, 15]

Systems based on assessments of dependency/acuity are the most widely used [17] and this is confirmed by our recent RN4CAST survey. [7] The SNCT falls into this category. Patients are allocated to one of five acuity dependency categories. These categories indicate requirements for nursing care based on an estimate of the time taken to care for patients in each category. [5] It is distinctive because its care 'multipliers', used to estimate staffing requirements from patient dependency/acuity categories, have been derived from observations on approximately 1000 NHS wards and was most recently applied to 40,000 patient care episodes, making it a strong candidate for the NHS. [5, 18] However, in the face of rapidly changing care needs, it is not clear how long even such relatively extensive validation can be relied upon. Furthermore, even if average nurse staffing requirements are accurately estimated, the costs and consequences of basing daily staffing on an average using the tool are unknown. Alternative approaches to setting required staffing levels, including flexible approaches, have not been explored.

Because some form of systematic planning process is present in most Trusts, trials are no longer feasible. However, our modelling approach will provide robust estimates of the costs and consequences (in terms of adverse outcomes) of different approaches to using tools and workforce deployment policies based upon the results. We focus on the SNCT because it is currently the only tool endorsed by NICE and appears to be the most widely used tool in England. However, the lessons learned from this study would have implications for the use of any similar tool and flexible staffing policies based upon assessments made using them. Furthermore, our design would be adaptable should any of the participating Trusts choose to adopt an alternative tool following (e.g. future NICE endorsement) prior to study commencement.

This research aims to fill a number of significant gaps relating to safe and effective nurse staffing in hospital wards. It aims to determine the feasibility and likely costs and consequences of a number of approaches to dealing with fluctuations in workload, as assessed by the only tool that is currently endorsed by the National Institute for Health and Care Excellence (NICE). This study has the potential to tackle several key knowledge gaps in this field and the results will support local managers and decision makers to achieve safe nurse staffing consistently on NHS hospital wards.

We will consider options for flexible staffing policies using different baseline staffing and model the feasibility and costs / consequences of each staffing strategy including:

- Opportunities to redeploy staff from 'over-staffed' wards to 'under-staffed' wards
- Costs of bank / agency staff to be employed to fill critical staffing deficits

For peer review only - http://bmjopen.bmj.com/site/about/guidelines.xhtml

- The relative efficiencies of different staffing groups to meet patient need
- Adverse outcomes associated with under-staffing

Aims and objectives

In this study on acute medical surgical wards in 4 hospitals we aim to assess a range of options for using the Safer Nursing Care Tool and model the costs and consequences of various ward staffing 'policies' based on dependency/acuity assessments derived from the tool. We will assess:

- The required nursing establishment (daily staff equivalent), as measured by the tool using the recommended approach (based on average acuity/dependency of every patient on the ward over a minimum of 20 days).
- Daily variation in acuity / dependency of the patients as measured by the tool.
- The number of observations required to determine a reliable baseline.
- How current staffing on the wards matches staffing requirements measured by the tool.
- Whether a mismatch between staffing requirements measured by the tool and available staff is associated with nurse perceptions of staffing adequacy.
- The influence of non-patient factors such as admission/discharge rate.
- Develop computer based mathematical simulation models to explore scenarios with different approaches to using the tool and flexible staffing policies
- model the costs/consequences of each staffing policy including costs of bank/agency staff to be employed to fill critical staffing deficits, opportunities to deploy staff from overstaffed wards to understaffed wards and potential adverse outcomes associated with understaffing.

The study will provide evidence for the usefulness and accuracy of the SNCT, which is widely used in the NHS. The results of the study will give guidance on the feasibility and relative costs and consequences of a variety of staffing policies aimed at addressing fluctuations in demand, including flexible staffing.

Research Plan / Methods

In this study we will use the SNCT tool to assess acuity / dependency for all patients in each ward daily, over a period of 1 year. In a sub-sample we will undertake multiple daily observations periodically in order to assess variation throughout the day. For each shift we will also ask the nurse in charge to complete a brief report of perceived staffing adequacy based on a single item from our RN4CAST survey [19], reports of significant delayed or missed care [20], estimated staffing requirement (professional judgement) and reasons for any mismatch between available and required staffing. These nurse-reported assessments of staffing adequacy provide an external criteria for assessing SNCT accuracy and have been validated by relationships with patient care outcomes. [12, 21, 22]

From these data we address a number of questions. SNCT scores are designed to identify the required nursing establishment (employed workforce in WTE). From this, the daily staffing requirement can be inferred in nursing hours per day (NHPD). We will compare the establishment and daily NHPD as predicted using the SNCT scores with the actual establishment and staffing deployed on the ward. In order to assess whether the tool accurately predicts required staffing we will assess associations between deviations from planned staffing and measures of staffing adequacy. Using mathematical

For peer review only - http://bmjopen.bmj.com/site/about/guidelines.xhtml

models (e.g. [9, 23]) we will attempt to identify if there is an optimal approach to planning ward staffing using the tool and whether this varies across settings.

Using a range of criteria, we will determine the proportion of days that wards are critically under / over staffed if staffing / establishment were based on a range of policies for using the SNCT results. The policies to be considered will include:

- setting staffing to meet the mean patient acuity/dependency determined from 20 days observation (the SNCT standard approach);
- staffing to meet the maximum commonly observed acuity/dependency observed during baseline observation (maximum staffing approach) and
- a flexible staffing approach with ward establishments set to meet the minimum commonly observed dependency from baseline (and deficits filled by temporary staffing)
- other staffing policies, as determined by an expert / PPI reference group

We will assess the extent to which adding allowances for factors not incorporated into the tool (e.g. variability in admissions / discharge rates) changes daily staffing requirements.

Critical understaffing will be defined as 25% or 8 nursing hours per shift below the required level or a patient to nurse ratio exceeding 8:1 (whichever is reached first), as described in NICE safe staffing guidance. Needleman's study showed an increase in the risk of death for patients exposed to shifts when available nurse staffing was 8 hours or more below target. [3, 10, 24]

Using evidence on potential adverse outcomes associated with understaffing derived from robust observational studies, e.g. [10-12], we will create dynamic models of the costs and consequences of the staffing policies for meeting the fluctuations in demand considering:

- establishment costs
- availability and costs of bank / agency staff to be employed to fill critical staffing deficits
- opportunities to redeploy staff from overstaffed wards to understaffed wards
- relative efficiency of permanent vs temporary staff
- adverse outcomes associated with residual staffing variation

Setting

This is an observational study in medical / surgical wards in 1 university hospital, 2 district general hospitals and one specialist (oncology) hospital based in London, South East and South West England. The Trusts serve diverse populations including rural areas, deprived inner city populations and specialist national referrals. Across the four Trusts there are approximately 75 adult medical / surgical wards (excluding ICU and highly specialised areas) with over 1700 beds. Currently, staffing is reviewed periodically (up to three times per year). Two of the four trusts currently use the SNCT combined with professional judgement to review nursing establishments, as recommended by NICE. The remaining Trusts use other approaches but both plan to move to using the SNCT prior to study commencement. This range of settings is typical of those in which the SNCT is currently deployed and will allow us to explore the extent to which results might vary across settings or be generally applicable (as per the intention of the tool).

For peer review only - http://bmjopen.bmj.com/site/about/guidelines.xhtml

Sample / data sources

Eligibility criteria for inclusion in the study are:

- Ward provides inpatient care for 7 days per week.
- Adult somatic health population / medical or surgical
- Appropriate for SNCT according to the SNCT resource pack [4]

Exclusion criteria

- Wards that are assessed as providing highly specialised services (such as maternity) with atypical staffing requirements (as determined by local chief investigator, with documented reason)
- Day case / weekday wards
- ICU

For each hospital and ward we will develop a profile detailing current ward establishments (and any subsequent changes during the study), specialities, approaches to setting ward establishment and review of staffing adequacy including methods to identify and rectify staffing shortfalls on a given shift. In addition, we will determine basic ward layouts and the proportion of beds in bays / single rooms, factors that may influence staffing requirements. [25, 26] These data will be gathered from Trust reports and interview with staff responsible for setting and monitoring nursing establishments.

We will gather anonymised data on patient flow (admission, discharge and length of stay) and case mix (age, admission method, main specialty, HRG) from the each hospitals' patient administration system.

Staffing requirements / dependency acuity

Daily SNCT assessments on patients will be undertaken by nurses in charge of the shift. Using the supporting material developed for the SNCT, all nurses making assessments will be trained in the tool's use. [4] Published data indicates high reliability between raters [5] confirmed by our pilot testing. An overall agreement of 92% was reported at the level of the individual patient rating, and all disagreements being between categorisations of lower dependency patients, where differentials between staffing requirements (according to the tool's multipliers) are relatively small. Consequent impact on calculated ward staffing levels is generally negligible. Data will be collected over a one year period in order to ensure that seasonal variation is fully accounted for. Three of the four trusts in the study will have installed systems for inputting data into local databases via ward-based computers. In the remaining Trust we will deploy a bespoke spreadsheet for gathering data. While this generates a substantial requirement for data collection we are aware that this practice is routine in many Trusts and our own piloting indicates that it is feasible, taking only a few minutes to complete. Furthermore, although we might be able to undertake the study with a sample from across the year, the volume of data required would still be large and we judge it more feasible to establish the practice as a normal part of ward routine than to undertake frequent intermittent assessments. Co-applicants in all participating Trusts are confident that the systems can be implemented. In order to support the implementation of data collection and maximize response rates we will organize a programme of training and induction into data collection starting 2 months prior to the main study data collection period We will include a run-in period for data collection to assess feasibility and monitor compliance. and offer monthly feedback on compliance to investigators in Trusts and to individual wards throughout the study

For peer review only - http://bmjopen.bmj.com/site/about/guidelines.xhtml

with additional tailored training and support offered to wards whose compliance falls substantially below average compliance. Wards where overall compliance (response rates) falls below 50% will be targeted for immediate support.

The recommended approach to assessing the SNCT is to gather data at 15.00. [4] This will be undertaken on all participating wards every day for 1 year – 356 ward level assessments per ward. Based on our piloting we estimate that the assessments will take approximately 6 minutes per ward per day. In order to assess additional variation throughout the day we will undertake additional data collection. For one week (7 days) during the study period, ratings will also be undertaken after morning and evening shift handovers (approx. 8pm and 8/10 pm depending on shift patterns) on a stratified sample of wards.

Data will be collected at the level of the patient (anonymised) so any future changes in the recommended SNCT multipliers can be accommodated. Currently the SNCT multipliers generate an establishment (whole time equivalent nurses to be employed). Daily staffing requirements (Nursing Hours per day - NHPD) will be calculated from the SNCT establishment, which includes an 'uplift' of 22% for study and sick leave and a 20% WTE allowance for senior staff administrative duties by removing these 'uplifts' and dividing all staff hours across the year.

$$NHPD = \frac{(Establishment\ WTE - .2)}{122 * 365}\% * weekly\ working\ hours\ (37.5) * weeks\ worked\ (45)$$

This gives an indication of the nursing hours required on a given day, given the patient acuity level according to the SNCT tool. As the SNCT gives no indication of how staff are to be deployed across shifts we will make initial shift level estimates of staffing requirements by allocating hours to shifts based on planned deployment patterns for each ward. For example, if a ward runs two 12-hour shifts and typically has four staff at night and six during the day we will allocate calculated staffing requirements in proportion (40% night, 60% day). As the SNCT does not indicate the required staff skill mix we will take this same approach, using current ward rosters, to estimate ratios of qualified to unqualified staff per shift.

Deployed staffing

Data on deployed nurse staffing will be derived from Trusts e-rostering systems, which record nursing hours / grades and times, actually worked per ward per day. Depending on the trust, bank and agency staff employed are recorded on separate systems. These will also be interrogated.

Outcomes

In order to assess the validity of SNCT staffing predictions, we will measure the perception of staffing adequacy using a 'micro survey' for the nurse in charge on each shift to assess professional judgement. Professional judgement remains a leading alternative approach to determining nurse staffing requirements [5, 17] and is seen as an essential adjunct to measurement systems, as recognised by NICE. [3] The nurse in charge will report staffing adequacy, based on a single item from our RN4CAST survey [19] ("on this shift, do you have enough nurses to provide quality patient care"); reports of significant delayed or missed care [20] ("on this shift was necessary nursing care left undone because

For peer review only - http://bmjopen.bmj.com/site/about/guidelines.xhtml

staff lacked time to complete it"); and estimated staffing requirement (estimated number of RNs and HCSW required). These nurse-reported assessments of staffing adequacy provide an external criteria for assessing SNCT accuracy and have been validated by relationships with patient care outcomes. [21, 27, 28] To assess within day variation we will also assess these measures three times per day over one week (see above).

Analysis

For the purposes of descriptive analysis, we will form groupings of wards based on a-priori criteria (e.g. named specialty) and / or empirically derived criteria (e.g. high turnover, high acuity). Identification of relevant grouping will be informed by consultation with a reference group of clinical experts recruited from individuals with responsibility for setting staffing establishments in participating Trusts and additional experts in staffing methodologies.

We will undertake descriptive analyses to identify central tendency (mean, median) and variation (SD, range and interquartile range) in SNCT acuity/dependency ratings. We will explore variation over time by looking at trends throughout the year although depending on any observed pattern it may not be possible to distinguish secular variation from recurring seasonal variation. We will also explore variation by day of week. From the sub-sample of three times daily observations we will also calculate the magnitude of variation within a day by calculating intracluster correlation coefficients between observations taken within a single ward on one day. We will describe actual staffing patterns in a similar manner

We will identify indicative daily staffing requirements from the SNCT, using the methods outlined above. The indicative staffing requirement will be based on the first 20 days of data. We will consider the appropriateness of the 20 day sample window of the SCNT and its impact on establishment. Areas of investigation will include the timing of the sampling and length of the sampling period, for example, a comparative analysis between establishment based on 20 and 60 day measures and a comparison of the estimate obtained from months with high and low average scores.

In order to give an indication of the validity of SNCT predicted staffing requirements (professional judgement), we will calculate the deviation between predicted staffing requirements from the SNCT (per shift and per day) and the actual staffing deployed. Using multi-level modelling approaches appropriate to the type of data we will assess the relationship between deviation from predicted staffing requirement and nurse reported measures of perceived staffing adequacy (including staffing levels and skill mix). We will derive three outcome measures: 1) binary measure of staffing being perceived as adequate 2) deviation between nurse reported requirement and available staffing (all RN+HCSW) and 3 deviation between nurse reported skill mix requirement and actual skill mix (RN/RN+HCSW). Beginning with a simple model of the association between deviation from staffing required and the measure of staffing adequacy we will add (multi-level) effects for ward type (ward clustered in ward type) and hospital (ward clustered in hospital). We will test models with both random slopes and random intercepts. Reduction in the Bayesian information criterion (BIC) and Akaike information criterion (AIK) will be used as the criterion for model selection. We will also assess whether inclusion of daily patient turnover (admissions, discharges) and ward layout improves model fit and whether there is interaction between

For peer review only - http://bmjopen.bmj.com/site/about/guidelines.xhtml

these terms and staffing deviation. All statistical modelling will be undertaken using the analysis software STATA.

If the SNCT provides an accurate prediction of staffing requirements, there should be a significant inverse association between deviation from the required staffing and reported staffing adequacy (professional judgement). If the overall results indicate that the SNCT gives a good estimate of required staffing levels and skill mix then the current approach to using the tool is validated. If the degree of relationship varies according to the other factors included in the model (indicated by including random slopes in the multi-level model and significant interaction between staffing adequacy and turnover / ward types / hospital) this will indicate that the accuracy of the SNCT prediction may vary according to these factors, because the relationship between deviation and staffing adequacy differs, and thus that additional information is required to estimate the staffing required. In particular, we will explore whether there is specific evidence indicating that the specialist cancer hospital may differ from other offering more 'general' services and whether traditional ways of categorising wards (e.g medical, surgical, older people) for benchmarking staffing requirements are, in fact, useful discriminators. We will also assess specifically whether perceived adequacy of skill mix varies according to ward type and assess whether results give any indication of the necessary skill mix on particular wards.

Operational Research Modelling

Model parameterisation & conceptualisation

The SNCT and ward data collected will be used as the empirical basis for an analysis of staffing policies by simulation. Stochastic simulation models consist of a number of continuous and discrete random variables used to mimic the variability seen in a real ward and hospital. A stochastic simulation is the appropriate methodology as wards are subject to variation in patient admission rates, patient length of treatment, patient acuity and are subject to capacity constraints. In particular, we will make use of a discrete-event simulation framework to model each ward within the hospital.

We will construct a model of a hospital with a finite number of beds and wards. Parameters in the model will be derived from observations of wards within each of the 4 hospitals. We will select and fit appropriate probability distributions using maximum likelihood estimates of parameters. The model will run to simulate data for a period of one year for each hospital and for each comparative analysis.

As admissions are conceptualised as a random variable, there will be instances where the model generates a number of patients whom cannot be immediately admitted to the ward. In these instances we will assume the patient is admitted as an outlier to an alternative ward. Although we expect the acuity of patients to vary during their stay in hospital, it is not feasible to track how the SNCT estimate of acuity/dependency varies in an individual patient during data collection. The model will therefore treat acuity as an independent random variable from patients, but will still incorporate transient effects, such as acuity by time of day.

Comparative analysis

We will model and compare a range of staffing strategies

- Standard staffing – Staffing levels based on a 20 day SNCT average
- Maximum staffing – Staffing based on the maximum requirement seen in 20 days of SNCT data

For peer review only - http://bmjopen.bmj.com/site/about/guidelines.xhtml

- Minimum / flexible staffing (base staffing set to meet minimum need observed in 20 days of SNCT data)
- Any alternative scenario suggested by the clinical expert / PPI reference group

Assuming a normal distribution model applies, base staffing levels for maximum and flexible staffing levels will be set using mean + (or minus) 1.65 SD in order to have staffing that is sufficient 95% of the time (or a staffing level set based on the lowest that is commonly observed). We will investigate the feasibility of further comparative scenarios using measures of sample error and variation, such as the standard deviation, and methodology from statistical process control.

For flexible and standard models, we will assess the feasibility of flexible staffing by calculating a hospital level estimate of staffing required and comparing this to the number of nurses deployed according to the model. For flexible staffing policies and the standard staffing model we will determine the feasibility of filling staffing shortfalls from redeployment within the hospital firstly and bank and agency staff secondly. We will work with our expert reference group to determine triggers and priority rules for the point at which more resource is needed and why one ward might get the resource over another if there are insufficient resources. Any residual difference would be an estimate of requirements for bank / agency nurses. We will use hospital level estimates for proportion of bank and agency shifts required vs those filled to model the likelihood of filling a shift from bank / agency. We will assess the implications of lower efficiency for temporary staff on wards by using varying assumptions about the relative efficiency of temporary staff (e.g. 1 temporary staff WTE = .8 permanent staff WTE)

Primary model outcomes

- No. (proportion) of days that are below the required nurse staffing level (NHPD)
- No. (proportion) of shifts that ward is below 8 hours or more of required nurse staffing
- No. (proportion) of shifts that ward is below 8 hours or more of required registered nurse staffing
- No. (proportion) of Day shifts where Registered nurse staffing is below 8 patients per nurse
- Number / proportion of shifts associated with reports of inadequate staffing
- Mortality at 30 days due to insufficient staffing
- Other adverse outcomes

In order to model shifts where professional judgement identifies staffing levels to be inadequate we will use coefficients from our regression models for the association between staffing shortfalls and the likelihood of reports of inadequate staffing. In order to estimate effects on outcomes we will scrutinise evidence for robust studies that estimate adverse effects in the face of nurse staffing below that required. Currently the only study we are aware of that provides estimates in this form is a US study by Needleman and colleagues. [10] This gives a hazard ratio of 1.02 for the effect of exposure to a shift with 8 hours or less below target staffing on 30 day mortality. Using hospital level mortality rates as a baseline we will use this to estimate the effects of understaffing in each ward. Because overall risk of mortality will not be uniformly spread across wards, we will also aggregate these estimates at a hospital level. Although wards included in the study will not cover all patients they are likely to include the majority of those at higher risk of mortality. Excluded areas will be have relatively few patients and / or be at relatively low risk of mortality (e.g. day surgery, paediatrics, maternity). While we exclude ICU, the majority of patients with an ICU stay will also experience a ward stay.

For peer review only - http://bmjopen.bmj.com/site/about/guidelines.xhtml

As we are collecting actual staffing levels over a period of one year we will be in a position to compare the modelled results based on SNCT establishment with actual understaffing. This will allow us to evaluate if improvement is achieved using the SNCT relative to the success of the staffing policies currently deployed in the Trust.

Bias handling in model estimates

As the model will begin with no patients in it, we will use standard methodology for handling initialisation bias to run the model to a representative starting state. We will also investigate the feasibility of using representative initial conditions, such as the average ward occupancy, instead or in addition to a run in period, as this will greatly reduce the runtime and manageability of the models. We will use standard methodology (multiple replications or non-overlapping/spaced batch means) to ensure that sufficient data is generated by the model to provide point estimates that are accurate assessments of model performance. We will investigate the feasibility of more advanced batched means methods such as overlapping batch means in reducing model runtime.

Parameter Uncertainty

We will investigate the impact of parameter uncertainty at both the individual ward level and the aggregate hospital level results level using a combination of one-way and multiple-way sensitivity analyses.

Economic modelling

Nurse staff costs will be estimated for each shift, for each ward, based on the number of staff (at each band) and the length of shift (or time working on each shift for those nurses working across shifts) to derive total whole-time equivalents. We propose reporting an overall breakdown of registered vs unregistered nurses. Each of these broad groups will be costed based on each ward's existing grade/ skill mix of staff, within these broad categories. These definitions are aligned with nationally representative unit costs [29], which will be used as the basis of costing the nursing staff on all included wards (see Table). The Unit Costs will be applied to agency and bank staff. However additional charges will be included to account for additional costs associated with employing agency staff. If the published unit costs are considered inappropriate for any of the staff categories included in the study, new estimates will be calculated by the study team using consistent methodology. Ward costs will also be calculated using Trust-level WTE costs to determine the robustness of the costing approach to local variations in estimating staff costs.

Category of nursing staff	Salary	On-costs†	Overheads			Total
			Staff ‡	Non-staff*	Capital #	
Mean FTE basic salary for Agenda for Change band 7	38,345	9,598	9,257	20,121	2,752	80,073
Mean FTE basic salary for Agenda for Change band 6	31,943	7,818	7,677	16,687	2,752	66,877
Mean FTE basic salary for Agenda for Change band 5	25,847	6,123	6,173	13,417	2,752	54,312
Mean FTE basic salary for Agenda for Change band 4	21,120	4,493	4,945	10,749	2,173	43,480
Mean FTE basic salary for Agenda for Change band 3	18,433	3,922	4,316	9,382	1,896	37,949

For peer review only - http://bmjopen.bmj.com/site/about/guidelines.xhtml

Identifying nurse-staffing requirements using the Safer Nursing Care Tool.

Mean FTE basic salary for Agenda for Change band 2	16,282	3,464	3,812	8,287	1,675	33,520

Notes
† employer NI contribution plus 14% salary for employer's contribution to superannuation
‡ Management, administration and estates staff at 19.31% of direct salary costs
* Costs for office travel/ transport and telephone, education and training, supplies and services, plus utilities, at 41.97% of direct salary costs
# based on new-build and land requirements of NHS hospital facilities, adjusted to reflect shared use. Treatment space not included. Annuitised over 60 years at discount rate of 3.5%

We will assume that projected staffing requirements (whole time equivalent across all nurse staff on the ward) will keep the same skill mix (in terms of registered and unregistered nurses and seniority of registered nurses). We may consider alternative assumptions, such as meeting all staffing shortfalls with registered nurses (since studies of safety of care suggest patient outcomes are most closely associated with levels of registered nurse staffing), as sensitivity analyses.

For the economic modelling, we will consider four main scenarios:

- Assume that staffing deficits will be met by employing bank/ agency staff, both registered and unregistered nurses. A sensitivity analysis will be conducted adjusting this scenario so that all staffing deficits will be met by registered bank/ agency nurses;
- Assume that staffing deficits will initially be met by redeployment from over-staffed wards (both registered and unregistered nurses) with bank/ agency staff used only when no further re-deployment is possible. A sensitivity analysis will be conducted adjusting this scenario so that all staffing deficits will be met by registered nurses.
- Assume that staffing deficits are not met
- Assume staffing deficits are partially met, assuming limited availability of bank and agency staff

The preceding analyses assume that bank/agency staff will be equally able to work on the ward as established staff. This may be the case for staff attached to a given ward for a period of time, or who are regularly employed on a given ward, but may not apply for staff deployed to a ward for a relatively short period of time (including staff who are temporarily redeployed within a single Trust). Sensitivity analyses will be conducted to consider the potential variation in relative efficiency of permanent versus temporary staff (by adjusting the multipliers when employing agency, bank or re-deployed staff). Because costs of flexible staffing policies can be shared across wards (the "cost" of a staff member moving from one ward to another could be attributed to either ward, depending on local accounting practices) and occur within a single organisation, we will aggregate staffing cost estimates at the hospital level.

Where feasible, we will model the cost of adverse consequences of deviations from predicted staffing requirements, although this will be dependent on identifying robust quantitative measures of association between staffing levels and outcomes. We have already identified the study by Needleman and colleagues [10] as potential basis for modelling mortality consequences of inadequate nurse staffing levels. Where such quantitative estimates exist, and where these can be combined with appropriate measures of baseline risk, an estimate of the impact of staffing levels on patient outcomes can be estimated. We will search for similar estimates relating to any adverse consequences (for example, falls) of nurse staffing levels falling below a defined required level. In order to cost these outcomes we will identify/ develop unit cost estimates that capture the additional costs (in terms of patient care) of the adverse outcomes.

For peer review only - http://bmjopen.bmj.com/site/about/guidelines.xhtml

Identifying nurse-staffing requirements using the Safer Nursing Care Tool.

We will consider and compare costs and consequences of each staffing strategy, including the current (actual) staffing, which will be taken as representing the strategy described in the trust profile as enacted in the real world.

Data management

SNCT acuity data will be electronically recorded initially at sites within a Microsoft Excel spreadsheet or within local e-rostering databases. These data will be anonymised, but will provide detail of time of day, shift, the site and ward. A sample of workforce, SNCT, ward admissions and discharge data will be extracted from each sites patient administration system (PAS) and e-rostering systems. It is envisaged that these data will be in a similar format across the research sites, although some cleaning of the data may be appropriate. An initial sample will be extracted from each site to explore differences. All data will be collected in a database (see below). Data will be checked for invalid and anomalous values (e.g. out of range values) and cleaned. Should overall response rates fall below 80%, we will assess data for evidence that missing data is related to workload. Specifically we will determine whether shifts with missed ratings are more likely to occur during periods of higher acuity / dependency (as assessed by previous / subsequent ratings) and develop strategies for dealing with missing data accordingly. Rules for dealing with missing data will be established in consultation with the SSC, according to data types and observed distributions. With careful data collection, we expect that missing data may be minimized, and the missing data mechanism may be carefully reconstructed to some extent. As any model fit to incomplete data extrapolates the missing data under some set of untestable assumptions, a sensitivity analysis to assumptions about the missing data mechanism is will be undertaken. We will review imputation methods, e.g. last observation carried forward and multiple imputation, parametric modelling under missing at random assumptions, and random effects modelling to deal with possible missing not at random mechanisms. If a missing not at random mechanism is plausible, we will explore the application of cutting-edge approaches in a likelihood-based framework.

Data storage and transfer

All raw SNCT and patient administration will be stored on secure encrypted servers at the University of Southampton with access restricted to the project team. The data will transferred to the project team by the University of Southampton's encrypted file transfer facility. All data will be anonymised at source. The volume of SNCT data collected is estimated to be large 365 days X 75 wards X 30 SNCT ratings per day, but its structure will not be complex. The same is true for all ward admission and discharge data, although this will require some processing to structure the data in a format suitable for analysis. The data will be imported to a relational database management system such as SQL Server Express 2014 installed locally on the SRF's encrypted University of Southampton computer system. All import and manipulation of data will be handled through T-SQL and stored procedures that will be backed up and stored on University servers. These procedures will be developed early in the project based on an initial sample of primary and routinely collected ward data.

Dissemination and projected outputs

A range of dissemination approaches will be used to target different audiences for the research. We will produce a final research report for the NIHR journals library detailing all the work undertaken and

For peer review only - http://bmjopen.bmj.com/site/about/guidelines.xhtml

including supporting technical appendices, an abstract and an executive summary focused on results/findings and suitable for use separately from the report as a briefing for NHS managers.

We will also prepare a set of 10 PowerPoint slides presenting the main findings from the research, designed for use by the research team or others in disseminating the research findings to the NHS. The slides will be made available alongside the report on the HS&DR programme website and, where possible, as additional material linked to any other publications.

We will submit abstracts for oral presentation for at least one national conference and one international conference focussing on nursing workforce / patient safety. We will disseminate summaries of findings and implications via journals such as the HSJ, Nursing Standard and Nursing Times; networks such as the Health Services Research Network and NHS employers and via networks of key stakeholders. Our PPI and service representatives on the project steering group will guide us in developing a dissemination strategy for these audiences as findings emerge. We will work closely with the University media team and ensure that members of the project team are given full support and training in dealing with media enquiries.

We will prepare at least three academic papers and publish these open access, in high impact journals. The focus of these will be:

1. Variation in SNCT acuity dependency by time of day and day of week
2. Variation in staffing / staffing adequacy between wards / specialities and over time
3. The costs / consequences of different staffing policies.

We aim to publish as early as possible and within the project life span with publications contributing to (rahter than deriving from) the final report. In particular, we aim to publish paper 1. above shortly after data collection is complete. We will also run one workshop for NHS nurse / workforce leaders to disseminate findings and to further consider the implications of the research.

We will use our established social media networks which include organisational (e.g. @Wessexclahrc) and personal professional (e.g. @workforcesoton) twitter accounts with substantial following to promote all project outputs.

Outputs

The study will provide data that can identify the relative feasibility and the costs / consequences of a variety of different staffing policies. This study will provide more direct evidence than is currently available to inform decisions about staffing levels and flexible staffing policies in an NHS context, and so can contribute significantly to future revisions of such guidance.

The research has the potential to have a number of impacts. The findings have wide utility throughout the acute hospital sector of the NHS. Nurse staffing is a vital issue as nursing staff account for a substantial proportion of the wage bill and the significance of nurse staffing in maintaining patient safety and quality experience has been established in many studies. However, the NICE safe staffing guidance noted a lack of directly relevant evidence to guide safe staffing. To our knowledge this will be the first study in the world conducted using the SNCT other than those by its developers and the only study to have modelled the costs and consequences of using it to guide flexible staffing policies. Our

For peer review only - http://bmjopen.bmj.com/site/about/guidelines.xhtml

study will also provide an independent assessment of the validity of this widely used tool and guidance on how its use might be adapted to deliver optimal staffing solutions.

Our Steering group / reference panel will include senior stakeholders including senior nurses from the large acute NHS Trusts and a representative from the Shelford group's chief nurses' subgroup (who have supported the SNCT). Through the established networks of these members we will share our findings with those leading national and local implementation of NICE safe staffing guidance and action area 5 of NHS England's compassion in practice strategy for nursing "ensuring we have the right staff, with the right skills in the right place".

We anticipate developing these lines of research by means of a future NIHR programme grant and through our extensive networks of international collaboration in this area, linking to other planned research that will allow us to estimate directly consequences for patients by using local clinical data. Immediate project outputs will include the workshop and other dissemination material identified under our dissemination strategy, which will form the core of a toolkit for NHS managers.

Plan of investigation and timetable

Month	Pre project (Year -1)	Year 1	Year 2	Year 3
1		Project start up meet.<br>Project Steering Group meet<br>Literature review / background for papers (to month 6)	Project Steering Group meet<br>OR modelling	Project Steering Group meet<br>Draft modelling / economics papers
2		Initiate R&D approval	OR modelling continues	Review and finalise drafts / submit papers
3		Specification for local data extraction	OR modelling continues	Prepare final report to NIHR
4		Develop local context profiles	OR modelling continues	Prepare final report to NIHR / revise papers
5		Pilot data extraction (PAS, staffing)<br>Build database (2 months)<br>Pilot data collection / run in	OR modelling continues	Prepare final report to NIHR / revise papers
6		Project Steering Group meet<br>Preliminary modelling<br>Pilot data collection / run in	Data collection ends<br>OR modelling continues	Stakeholder event<br>Final report NIHR
7	Initiate ethics application	Data collection begins / data extraction (12 Mo)	Project Steering Group meet<br>Final data extract<br>OR modelling parameterisation / testing<br>Economic modelling begins	
8		Data collection continues	Modelling /analysis continues	
9		Data collection continues	Descriptive analysis	
10		Data collection continues	Modelling /analysis continues	
11	Initiate appointments (if required)	Annual report prep<br>Data collection continues	Modelling /analysis continues	
12		NIHR annual report<br>Data collection continues<br>Data extract	Modelling /analysis continues<br>Draft modelling / economics papers	

For peer review only - http://bmjopen.bmj.com/site/about/guidelines.xhtml

Identifying nurse-staffing requirements using the Safer Nursing Care Tool.

Project management

The project will be overseen and managed by PG with day-to-day project management provided by a senior RF with admin support. JJ or TM will deputise in the case of unforeseen periods of absence. The work of the project will be organised into 6 work streams with senior leads. Project management (PG), data acquisition (ARS), data management (TM), Stats modelling (AM) economic modelling (JJ) and OR modelling (TM). Leads will hold monthly research management group meetings to review and coordinate progress on each work stream. A specific liaison group including the lead investigator in each trust, will work to establish and oversee mechanisms of data acquisition and transfer. Project progress will be monitored monthly against key milestones. Because of the complexities of the modelling and associated analyses, we have allowed significant time for analysis and for write up following the building of the database. This timescale also gives some degree of contingency should we experience delays in accessing data or building the database.

Strategic oversight and advice will be provided by a study steering committee (SSC), with a wider reference panel also recruited to provide specific advice including a PPI perspective. The SSC will meet twice per year during the project.

Approval by ethics committees

We have registered with IRAS (project ID is 190548). Our self-assessment using the tool provided by NRES indicates that this study will not require formal ethical approval by that body because no data is collected directly from patients and reports of patient acuity / dependency ratings are entirely anonymous. We see no substantial ethical issues arising from the research. While results of the SNCT staffing tool may indicate staffing shortages and staff reports may also indicate concerns Trusts already have established mechanisms for dealing with these issues. However, in order to get external assurance and to remove any potential barriers to publication we will seek scrutiny from the University Ethics committee.

Patient and Public Involvement

Safe staffing in hospitals is a major area of public concern following the Francis Inquiry and other reports. This proposal has been developed with this concern in mind but it addresses specific questions that arise from the commissioning brief which in turn follow from gaps in the evidence identified by (among others) the National Institute of Health and Care Excellence (NICE) in their guidance of safe staffing in hospital wards. The developing proposal was discussed with Claire Ballinger (Patient and Public Involvement [PPI] lead for the NIHR CLAHRC Wessex) and Anya de Iongh (PPI champion for the 'fundamental care in hospital' theme of the CLAHRC). Following their guidance we sought no further direct patient / public involvement in prioritising or shaping the questions as these arise from the brief and need for technical assessment of the tool, we have focussed on considering how the public could be involved in the proposed research, its governance and dissemination.

Based on this advice, we have sought a lay member of our Steering group with specific interest and expertise. To this end, we have worked with Stephen Habgood, a lay member of the NICE safe staffing advisory committee for the development of guidelines in mental health, who has agreed to participate in the project Steering group. Stephen also has experience of staffing methodologies used in other sectors

For peer review only - http://bmjopen.bmj.com/site/about/guidelines.xhtml

from his past work as a prison governor and is currently chair of a charity concerned with young people's suicide. These combined experiences means that he is actively interested in the specific topics and can fully participate in the Steering group based on prior expertise. We will recruit a second lay member of the Steering group via networks established through the NIHR CLAHRC and the Wessex Involvement in Service Research and Delivery (WISeRD) group – which includes both patients and members of the public. We will also recruit additional members to our reference panel with a particular focus on giving insights into issues that arise from models to be tested and aiding the selection of models that we consider. Training requirements will be assessed and support provided as required, drawing on expertise and facilities provided by the local RDS and NIHR CLAHRC Wessex. We will ensure that these lay members are enable to be full and active members of the Steering group, giving insight and expertise across all aspects of the study. We anticipate in particular that they will orient the team to wider (industry, public, service user) perspectives on safe staffing and advise on public facing dissemination activities.

Expertise

The team brings strengths in health services research, workforce, NHS workforce management operational research and health economics from the University of Southampton, NIHR CLAHRC (Wessex) and partner NHS Trusts. This team provides the expertise to deliver this project, which draws on a unique set of linked data. The PI (PG) has extensive experience of managing large research teams undertaking complex secondary data analysis (including multi-level modelling) in the field of nurse staffing. JEB is an expert on nurse staffing and large scale workforce surveys. She will provide advice and expertise in relation to relevant factors that will shape the modelling and will design and oversee the survey elements of the study. PG and JEB were leads in the "RN4CAST" study, one of the largest nursing workforce studies ever undertaken. JJ is an experienced senior health economist with considerable experience in undertaking economic modelling for NICE technology appraisals. He will oversee the work of research fellows / associates undertaking the economic aspects of the study. This core team recently undertook evidence reviews for the NICE safe staffing guidance. AM is a statistician and health economist with specific expertise in time-varying clustering of multivariate longitudinal data and current expertise on another HSDR funded nurse staffing study. These will be joined by TM, an operational researcher whose research interests include applied healthcare modelling within the NHS, the role modelling plays in decision making in organisations and statistical issues in simulation, ARS, who is currently working with the CLAHRC to link data across trusts in Wessex and NS, RC, YJ, AD & NP who are senior nursing leads with responsibility for implementing staffing policies, including the use of the SNCT. These latter will provide access to staffing data, expertise on its interpretation and be involved in shaping the scenarios for models. TM will supervise the OR modelling and ARS coordinate routine data returns across trusts.

For peer review only - http://bmjopen.bmj.com/site/about/guidelines.xhtml

Identifying nurse-staffing requirements using the Safer Nursing Care Tool.

References

1. The Mid Staffordshire NHS Foundation Trust Inquiry and Chaired by Robert Francis QC, *Independent Inquiry into care provided by Mid Staffordshire NHS Foundation Trust January 2005 – March 2009*. 2010, London: The Stationary Office.
2. Keogh, B., *Review into the quality of care and treatment provided by 14 hospital trusts in England: overview report*. 2013, NHS.
3. National Institute for Health and Care Excellence (NICE), *Safe Staffing for Nursing in Adult Inpatient wards in Acute Hospitals (Guidance)*. 2014, National Institute for Health and Care Excellence London.
4. *Safer Nursing Care Tool Implementation Resource Pack*. 2014, The Shelford Group.
5. Smith, S., et al., *Developing, testing and applying instruments for measuring rising dependency‐acuity's impact on ward staffing and quality.* International Journal of Health Care Quality Assurance, 2009. **22**(1): p. 30-39.
6. Griffiths, P., et al., *The association between patient safety outcomes and nurse/healthcare assistant skill mix and staffing levels and factors that may influence staffing requirements (NICE evidence review)*. 2014, University of Southampton Centre for innovation and Leadership in Health Sciences.
7. RN4CAST survey, *unpublished data.*
8. Gabbay, U. and M. Bukchin, *Does daily nurse staffing match ward workload variability? Three hospitals' experiences.* Int J Health Care Qual Assur, 2009. **22**(6): p. 625-41.
9. Allen, M. and S. Thornton, *Providing one-to-one care in labour. Analysis of 'Birthrate Plus' labour ward staffing in real and simulated labour ward environments.* BJOG, 2013. **120**(1): p. 100-7.
10. Needleman, J., et al., *Nurse staffing and inpatient hospital mortality.* N Engl J Med, 2011. **364**(11): p. 1037-45.
11. Kane, R.L., et al., *The Association of Registered Nurse Staffing Levels and Patient Outcomes: Systematic Review and Meta-Analysis.* Medical Care, 2007. **45**(12): p. 1195-1204 10.1097/MLR.0b013e3181468ca3.
12. Aiken, L.H., et al., *Nurse staffing and education and hospital mortality in nine European countries: a retrospective observational study.* Lancet, 2014. **383**(9931): p. 1824-30.
13. National Quality Board, *How to ensure the right people, with the right skills, are in the right place. A guide to nursing midwifery and care staffing capacity and capability'*. 2013: http://www.england.nhs.uk/wp-content/uploads/2013/11/nqb-how-to-guid.pdf.
14. Edwardson, S.R. and P.B. Giovannetti, *Nursing Workload Measurement Systems.* Annual Review of Nursing Research, 1994. **12**(1): p. 95-123.
15. Fasoli, D.R. and K.S. Haddock, *Results of an integrative review of patient classification systems.* Annual Review of Nursing Research, 2010. **28**: p. 295-316.
16. DHSS Operational Research Service, *Nurse Manpower Planning: Approaches and Techniques*. 1982, DHSS: London:.
17. Harper, P.R., N.H. Powell, and J.E. Williams, *Modelling the size and skill-mix of hospital nursing teams.* Journal of the Operational Research Society, 2009. **61**(5): p. 768-779.
18. National Institute for Health and Clinical Excellence, *Safer Nursing Care Tool (SNCT): statement of endorsement*. 2014.
19. Sermeus, W., et al., *Nurse forecasting in Europe (RN4CAST): Rationale, design and methodology.* BMC Nurs, 2011. **10**(6): p. 6.
20. Ball, J.E., et al., *'Care left undone' during nursing shifts: associations with workload and perceived quality of care.* BMJ Qual Saf, 2014. **23**(2): p. 116-25.
21. Smeds Alenius, L., et al., *Staffing and resource adequacy strongly related to RNs' assessment of patient safety: a national study of RNs working in acute-care hospitals in Sweden.* BMJ Quality & Safety, 2014. **23**(3): p. 242-249.
22. Aiken, L.H., et al., *Patient safety, satisfaction, and quality of hospital care: cross sectional surveys of nurses and patients in 12 countries in Europe and the United States.* BMJ, 2012. **344**(7851): p. e1717.
23. Kortbeek, N., et al., *Flexible nurse staffing based on hourly bed census predictions.* Memorandum 1996, Department of Applied Mathematics, University of Twente, Enschede, The Netherlands, 2012., 2012. **http://eprints.eemcs.utwente.nl/22408/01/memo1996.pdf**.
24. Fagerstrom, L. and A. Rauhala, *Benchmarking in nursing care by the RAFAELA patient classification system - a possibility for nurse managers.* J Nurs Manag, 2007. **15**(7): p. 683-92.
25. Hurst, K., *UK ward design: Patient dependency, nursing workload, staffing and quality--An observational study.* International Journal of Nursing Studies, 2008. **45**(3): p. 370-381.
26. Maben, J., et al., *Evaluating a major innovation in hospital design: workforce implications and impact on patient and staff experiences of all single room hospital accommodation.* Health Serv Deliv Res, 2015. **3**(3): p. 254.
27. Gunnarsdóttir, S., et al., *Front-line management, staffing and nurse–doctor relationships as predictors of nurse and patient outcomes. A survey of Icelandic hospital nurses.* International Journal of Nursing Studies, 2009. **46**(7): p. 920-927.
28. Schubert, M., et al., *Associations between rationing of nursing care and inpatient mortality in Swiss hospitals.* International Journal for Quality in Health Care, 2012. **24**(3): p. 230-238.
29. Curtis, L., *Unit Costs of Health and Social Care 2014*. 2012, Canterbury: PSSRU, University of Kent.

For peer review only - http://bmjopen.bmj.com/site/about/guidelines.xhtml

The RECORD statement – checklist of items, extended from the STROBE statement, that should be reported in observational studies using routinely collected health data.

	Item No.	STROBE items	Location in manuscript where items are reported	RECORD items	Location in manuscript where items are reported
**Title and abstract**					
	1	(a) Indicate the study's design with a commonly used term in the title or the abstract (b) Provide in the abstract an informative and balanced summary of what was done and what was found	a) Pg 1 Title "observational"<br>b) Pg 1 Abstract	RECORD 1.1: The type of data used should be specified in the title or abstract. When possible, the name of the databases used should be included.<br><br>RECORD 1.2: If applicable, the geographic region and timeframe within which the study took place should be reported in the title or abstract.<br><br>RECORD 1.3: If linkage between databases was conducted for the study, this should be clearly stated in the title or abstract.	1.1 "SNCT ratings" and "staffing adequacy questions" pg 1 Abstract<br>1.2 Not enough words. This is reported in the Methods pg 8 line 158-160<br>1.3 Pg 1 Abstract Design
**Introduction**					
Background rationale	2	Explain the scientific background and rationale for the investigation being reported	2. Pg 5-7		

For peer review only

Objectives	3	State specific objectives, including any prespecified hypotheses	Pg 7 Line 146 - 156		
**Methods**					
Study Design	4	Present key elements of study design early in the paper	Pg 7 line 146 "This observational study". Pg 8 line 158 "We used routinely-collected data and nurse reports…"		
Setting	5	Describe the setting, locations, and relevant dates, including periods of recruitment, exposure, follow-up, and data collection	See Methods Pg 158-160 pg 8 and line 167-172<br><br>Recruitment, exposure and follow-up are not applicable here.		
Participants	6	*(a) Cohort study* - Give the eligibility criteria, and the sources and methods of selection of participants. Describe methods of follow-up<br>*Case-control study* - Give the eligibility criteria, and the sources and methods of case ascertainment and control selection. Give the rationale for the choice of cases and controls<br>*Cross-sectional study* - Give the eligibility criteria, and the sources and methods of selection of participants	N/A	RECORD 6.1: The methods of study population selection (such as codes or algorithms used to identify subjects) should be listed in detail. If this is not possible, an explanation should be provided.<br><br>RECORD 6.2: Any validation studies of the codes or algorithms used to	6.1 pg 9 line 175-180<br><br>6.2 N/A

		(b) Cohort study - For matched studies, give matching criteria and number of exposed and unexposed<br>*Case-control study* - For matched studies, give matching criteria and the number of controls per case		select the population should be referenced. If validation was conducted for this study and not published elsewhere, detailed methods and results should be provided.<br><br>RECORD 6.3: If the study involved linkage of databases, consider use of a flow diagram or other graphical display to demonstrate the data linkage process, including the number of individuals with linked data at each stage.	6.3 Decided against given figure limits.
Variables	7	Clearly define all outcomes, exposures, predictors, potential confounders, and effect modifiers. Give diagnostic criteria, if applicable.	Outcomes - Pg 11 line 221-230 and Table 1.<br>Predictors – pg 12 line 263-267.<br>Control variables – pg 12 line 262 -267.	RECORD 7.1: A complete list of codes and algorithms used to classify exposures, outcomes, confounders, and effect modifiers should be provided. If these cannot be reported, an explanation should be provided.	Different data field names in different Trusts. Have explained how all variables derived – see Study Variables pg 9 line 191-
Data sources/ measurement	8	For each variable of interest, give sources of data and details of methods of assessment (measurement).	see Study Variables pg 9 line 191-		

For peer review only

		Describe comparability of assessment methods if there is more than one group			
Bias	9	Describe any efforts to address potential sources of bias	Sensitivity analysis conducted – line 245-246 pg 12		
Study size	10	Explain how the study size was arrived at	(Data was collected over a one year period in order to ensure that seasonal variation was fully accounted for.)		
Quantitative variables	11	Explain how quantitative variables were handled in the analyses. If applicable, describe which groupings were chosen, and why	See "Study variables" pg 9 line 191-<br><br>No groupings.		
Statistical methods	12	(a) Describe all statistical methods, including those used to control for confounding<br>(b) Describe any methods used to examine subgroups and interactions<br>(c) Explain how missing data were addressed<br>(d) *Cohort study* - If applicable, explain how loss to follow-up was addressed<br>*Case-control study* - If applicable, explain how matching of cases and controls was addressed<br>*Cross-sectional study* - If applicable, describe analytical methods taking account of sampling strategy<br>(e) Describe any sensitivity analyses	a) pg 12 line 259 – 263.<br><br>b) Subgroups – accounted for in nested multilevel structure.<br>Interactions - pg 18 line 273 -274<br><br>c) pg 10 line 198 for missing SNCT assessments.<br><br>d) N/A<br>e) Line 245-246, pg 12.		

Data access and cleaning methods		..		RECORD 12.1: Authors should describe the extent to which the investigators had access to the database population used to create the study population.<br><br>RECORD 12.2: Authors should provide information on the data cleaning methods used in the study.	12.1 This question is aimed at participant-based research and related ethical issues which do not apply here.<br>12.2 See Data cleaning and Analysis pg 11 line 233-.
Linkage		..		RECORD 12.3: State whether the study included person-level, institutional-level, or other data linkage across two or more databases. The methods of linkage and methods of linkage quality evaluation should be provided.	12.3 pg 14 line 299-301
**Results**					
Participants	13	(a) Report the numbers of individuals at each stage of the study (*e.g.*, numbers potentially eligible, examined for eligibility, confirmed eligible, included in the study, completing follow-up, and analysed)<br>(b) Give reasons for non-participation at each stage.	13. We did not have participants as such. Have explained which wards are eligible, see line 175-180 pg 9.<br>13b & c) NA	RECORD 13.1: Describe in detail the selection of the persons included in the study (*i.e.,* study population selection) including filtering based on data quality, data availability and	13.1 NA

For peer review only

		(c) Consider use of a flow diagram		linkage. The selection of included persons can be described in the text and/or by means of the study flow diagram.	
Descriptive data	14	(a) Give characteristics of study participants (*e.g.*, demographic, clinical, social) and information on exposures and potential confounders<br>(b) Indicate the number of participants with missing data for each variable of interest<br>(c) *Cohort study* - summarise follow-up time (*e.g.*, average and total amount)	14a. Table 2<br>14b. line 298-301 pg 14 describes missing data and sample size. We did not have "participants" as such.<br>14c. NA		
Outcome data	15	*Cohort study* - Report numbers of outcome events or summary measures over time<br>*Case-control study* - Report numbers in each exposure category, or summary measures of exposure<br>*Cross-sectional study* - Report numbers of outcome events or summary measures	15. line 331-334 pg 16.		
Main results	16	(a) Give unadjusted estimates and, if applicable, confounder-adjusted estimates and their precision (e.g., 95% confidence interval). Make clear which confounders were adjusted for and why they were included<br>(b) Report category boundaries when continuous variables were categorized	16a. Table 4 pg 17 odds ratios and 95% confidence intervals.<br>b. NA<br>c. NA		

For peer review only

		(c) If relevant, consider translating estimates of relative risk into absolute risk for a meaningful time period			
Other analyses	17	Report other analyses done—e.g., analyses of subgroups and interactions, and sensitivity analyses	Pg 18 line 381-384		
**Discussion**					
Key results	18	Summarise key results with reference to study objectives	See conclusions pg 24.		
Limitations	19	Discuss limitations of the study, taking into account sources of potential bias or imprecision. Discuss both direction and magnitude of any potential bias	Pg 23 Limitations section.	RECORD 19.1: Discuss the implications of using data that were not created or collected to answer the specific research question(s). Include discussion of misclassification bias, unmeasured confounding, missing data, and changing eligibility over time, as they pertain to the study being reported.	N/A
Interpretation	20	Give a cautious overall interpretation of results considering objectives, limitations, multiplicity of analyses, results from similar studies, and other relevant evidence	See conclusions pg 24.		
Generalisability	21	Discuss the generalisability (external validity) of the study results	Pg 24 line 508-509.		
**Other Information**					

For peer review only

Funding	22	Give the source of funding and the role of the funders for the present study and, if applicable, for the original study on which the present article is based	See funding statement pg 25		
Accessibility of protocol, raw data, and programming code		..		RECORD 22.1: Authors should provide information on how to access any supplemental information such as the study protocol, raw data, or programming code.	22.1 Protocol supplied as supplementary material as requested by journal. Link to data provided in Data sharing statement pg 26.

*Reference: Benchimol EI, Smeeth L, Guttmann A, Harron K, Moher D, Petersen I, Sørensen HT, von Elm E, Langan SM, the RECORD Working Committee. The REporting of studies Conducted using Observational Routinely-collected health Data (RECORD) Statement. *PLoS Medicine* 2015; in press.

*Checklist is protected under Creative Commons Attribution (CC BY) license.

For peer review only

BMJ Open

Performance of the Safer Nursing Care Tool to measure nurse staffing requirements in acute hospitals: a multi-centre observational study

Journal:	*BMJ Open*
Manuscript ID	bmjopen-2019-035828.R1
Article Type:	Original research
Date Submitted by the Author:	06-Mar-2020
Complete List of Authors:	Griffiths, Peter; University of Southampton, School of Health Sciences; University of Southampton, National Institute for Health Research Collaboration for Leadership in Applied Health Research and Care Wessex<br>Saville, Christina; University of Southampton, School of Health Sciences<br>Ball, Jane; University of Southampton, School of Health Sciences<br>Culliford, David; University of Southampton, School of Health Sciences; University of Southampton, National Institute for Health Research Collaboration for Leadership in Applied Health Research and Care Wessex<br>Pattison, Natalie; Royal Marsden NHS Foundation Trust, Clinical Services; University of Hertfordshire, School of Health and Social Work<br>Monks, Thomas; University of Southampton, School of Health Sciences; University of Exeter, University of Exeter Medical School
**Primary Subject Heading**:	Nursing
Secondary Subject Heading:	Health services research
Keywords:	Quality in health care < HEALTH SERVICES ADMINISTRATION & MANAGEMENT, Human resource management < HEALTH SERVICES ADMINISTRATION & MANAGEMENT, HEALTH SERVICES ADMINISTRATION & MANAGEMENT

SCHOLARONE™
Manuscripts

I, the Submitting Author has the right to grant and does grant on behalf of all authors of the Work (as defined in the below author licence), an exclusive licence and/or a non-exclusive licence for contributions from authors who are: i) UK Crown employees; ii) where BMJ has agreed a CC-BY licence shall apply, and/or iii) in accordance with the terms applicable for US Federal Government officers or employees acting as part of their official duties; on a worldwide, perpetual, irrevocable, royalty-free basis to BMJ Publishing Group Ltd ("BMJ") its licensees and where the relevant Journal is co-owned by BMJ to the co-owners of the Journal, to publish the Work in this journal and any other BMJ products and to exploit all rights, as set out in our licence.

The Submitting Author accepts and understands that any supply made under these terms is made by BMJ to the Submitting Author unless you are acting as an employee on behalf of your employer or a postgraduate student of an affiliated institution which is paying any applicable article publishing charge ("APC") for Open Access articles. Where the Submitting Author wishes to make the Work available on an Open Access basis (and intends to pay the relevant APC), the terms of reuse of such Open Access shall be governed by a Creative Commons licence – details of these licences and which Creative Commons licence will apply to this Work are set out in our licence referred to above.

Other than as permitted in any relevant BMJ Author's Self Archiving Policies, I confirm this Work has not been accepted for publication elsewhere, is not being considered for publication elsewhere and does not duplicate material already published. I confirm all authors consent to publication of this Work and authorise the granting of this licence.

For peer review only

Performance of the Safer Nursing Care Tool to measure nurse staffing requirements in acute hospitals: a multi-centre observational study

[Short title: Performance of the Safer Nursing Care Tool]

Peter Griffiths[1,2,] , Christina Saville[1]*, Jane E Ball[1], David Culliford [1,2], Natalie Pattison[3,4], Thomas Monks[1,5]

[1] School of Health Sciences, University of Southampton, Southampton, Hampshire, UK
[2]National Institute for Health Research Collaboration for Leadership in Applied Health Research and Care Wessex, University of Southampton, Southampton, Hampshire, UK
[3]Clinical Services, The Royal Marsden NHS Foundation Trust, London, UK
[4] School of Health and Social Work, University of Hertfordshire, Hatfield, Hertfordshire UK
[5] University of Exeter Medical School, University of Exeter, Exeter, Devon, UK

*Corresponding author: C.E.Saville@soton.ac.uk 02380 595282

For peer review only

Performance of the Safer Nursing Care Tool to measure nurse staffing requirements in acute hospitals: a multi-centre observational study

Abstract

Objectives

The best way to determine nurse staffing requirements on hospital wards is unclear. This study explores the precision of estimates of nurse staffing requirements made using the Safer Nursing Care Tool (SNCT) patient classification system for different sample sizes, and investigates whether recommended staff levels correspond with professional judgements of adequate staffing.

Design

Observational study linking datasets of staffing requirements (estimated using a tool) to professional judgements of adequate staffing. Multi-level logistic regression modelling.

Setting

81 medical/surgical units in 4 acute care hospitals

Participants

22,364 unit days where staffing levels and SNCT ratings were linked to nurse reports of staffing adequacy

Primary outcome measures

SNCT-estimated staffing requirements and nurses' assessments of staffing adequacy.

Results

The recommended minimum sample of 20 days allowed the required number to employ (the establishment) to be estimated with a mean precision (defined as half

For peer review only

the width of the confidence interval as a percentage of the mean) of 4.1%. For most units, much larger samples were required to estimate establishments within +/- 1 whole-time-equivalent staff member. When staffing was lower than that required according to the SNCT, for each hour per patient day of registered nurse staffing below the required staffing level, the odds of nurses reporting that there were enough staff to provide quality care were reduced by 11%. Correspondingly, the odds of nurses reporting that necessary nursing care was left undone were increased by 14%. No threshold indicating an optimal staffing level was observed. Surgical specialty, patient turnover and more single rooms were associated with lower odds of staffing adequacy.

Conclusions

The SNCT can provide reliable estimates of the number of nurses to employ on a unit, but larger samples than the recommended minimum are usually required. The SNCT provides a measure of nursing workload that correlates with professional judgements, but the recommended staffing levels may not be optimal. Some important sources of systematic variations in staffing requirements for some units are not accounted for. SNCT measurements are a potentially useful adjunct to professional judgement, but cannot replace it.

Trial registration

ISRCTN12307968.

[words 287]

Article summary

Strengths and Limitations of this study

- In contrast to most studies of nurse staffing tools, this was a large-scale study conducted in 81 units in 4 hospitals over a year.

For peer review only

For peer review only - http://bmjopen.bmj.com/site/about/guidelines.xhtml

- This is the first study to provide an independent evaluation of this tool, which is used in most hospitals in England.
- The observational study measured associations between staffing shortfalls measured using the SNCT and subjective professional assessments of staffing adequacy.
- The study did not explore the impact on objective care outcomes.

For peer review only

For peer review only - http://bmjopen.bmj.com/site/about/guidelines.xhtml

Introduction

In acute care hospitals, the ability to determine the 'right' number of nursing staff to employ and to deploy on any given shift is an imperative, as nurse staffing levels influence both efficiency and quality of care delivery. On the one hand, professional nurses and nursing support staff form the largest group of staff and the largest variable costs faced by hospitals. Nursing budgets are thus frequently targeted in the drive for cost savings.[1] On the other hand, inadequate nurse staffing is linked to deficits in the quality and safety of care.[2] However, despite the existence of many tools to determine staffing requirements and an extensive literature, evidence about the ability of any tool to reliably and accurately estimate staffing requirements is extremely limited.[3, 4] In this paper, we consider the "Safer Nursing Care Tool" (SNCT), which is used in the majority of acute hospitals in the United Kingdom's National Health Service(NHS) [5] and endorsed by the National Institute for Health and Care Excellence (NICE),[6] the body that produces evidence based guidelines for the NHS. We explore the reliability and precision of the estimates of required staffing establishments (that is the number of nurses to employ for a hospital unit) made using the tool and the extent to which estimated staffing requirements correspond with professional judgement of sufficient staffing. Despite the tool's widespread use and the importance of these considerations, these factors have not been previously studied.

Associations between higher registered nurse staffing levels in hospitals and improved care quality have been demonstrated in many studies. [2, 7-9] Outcomes include lower risks of in-hospital mortality [10], shorter lengths of stay [11] and fewer omissions of necessary care.[12] Such findings have underpinned policies to make minimum nurse staffing levels mandatory in some jurisdictions, for example in

For peer review only - http://bmjopen.bmj.com/site/about/guidelines.xhtml

California in the USA, some Australian states and more recently in Germany.[13] Yet studies showing associations between nurse staffing levels and outcomes rarely provide a clear indication of how many staff are needed for different patients, despite evidence that needs can vary considerably, and few studies have explored tipping points in relationships, which could be one indication of an optimal staffing level.[2, 14, 15]

Consequently, tools and systems to guide decisions about the number of nursing staff to employ, or to deploy on any given shift are still widely used either in conjunction with or as an alternative to mandatory minimums. At the heart of most tools is some form of patient level assessment, which is translated into a measure of required nursing time.[3, 4] Many such tools exist, although they are largely unsupported by robust evidence. Studies used to support the validity of tools to determine staffing requirements simply tend to show that staff demand estimated using a given tool correlates with some other measure of demand. In the absence of a gold standard, and without addressing whether the staffing according to the predicted level is sufficient to deliver the required care, such evidence is significantly limited. Different tools, while providing results that are highly correlated, can and do give dramatically different estimates of the staffing required by the same group of patients.[4] For example applying a new system to estimate the staffing required for low acuity wards resulted in an estimate that was double that derived from the existing system.[16]

Furthermore, although a key driver for choosing to use a tool is the assertion that variable patient need cannot be efficiently met by fixed staffing levels,[17] little consideration has been given to the impact of variation on the resulting estimates of average staffing requirements. Inter-rater reliability and agreement is often reported,

For peer review only - http://bmjopen.bmj.com/site/about/guidelines.xhtml

the precision with which the unit staffing requirement as a whole is estimated, either on a given day or over time, is not. [4]

The SNCT [18] is reported to be used in 80% of National Health Service acute hospitals in England.[5] The tool was originally designed to determine the required number of staff to employ (the establishment) for each unit to ensure that there are sufficient staff to fill daily rosters to meet average need, but it is increasingly used to monitor and determine daily demand for staff. It is not however used for billing purposes since in England billing is based on activity and does not explicitly account for nursing staff. The SNCT is an example of a patient classification system.[4] At least once per day, patients occupying beds on the ward are classified into one of five groups, based on their acuity and dependency on nursing care, with each group having an associated weighting (described as a 'multiplier') indicating the number of nursing staff required.[18] At the time of writing, the most recently published multipliers for general adult inpatient units were based on observations of 40,000 patient care episodes.[6] The multipliers represent the average of staff time to provide all direct patient care and ancillary work for patients in each group with allowances made for annual leave, study time and sickness absence when determining the number of nurses to employ. [18]

The SNCT has been shown to correlate strongly with an alternative classification system and high inter-rater agreement is reported. [19, 20] However, while the tool's handbook recommends a sample of at least 20 days to establish a reliable baseline for setting establishments, we could find no evidence of the precision of the resulting estimates. In our review of literature, we found no direct evidence that using this or any other tool improved the quality of care. [4] Therefore we used professional

For peer review only - http://bmjopen.bmj.com/site/about/guidelines.xhtml

judgement as the "gold standard", as we found no evidence that any tool provides a more accurate measure of the staffing required.

This observational study aims to provide evidence about the reliability and validity of the SNCT by addressing the precision of the estimated establishment and the extent to which staffing shortfalls relative to the level implied by the tool are associated with nurses' judgements that staffing levels are sufficient to deliver all necessary care with acceptable quality. Because factors such as patient turnover, specialty and layout are not directly considered in patient classifications yet may influence staffing requirements [21-23], we also examine the extent to which staffing levels determined using the SNCT are sufficient to accommodate variation in demand associated with these factors by determining whether there is an independent association between these factors and judgements of staffing adequacy when considering the effect of shortfalls from the SNCT recommended staffing level.

Methods and materials

This paper draws on research and data as described in detail previously in the NIHR Journals Library Health Services and Delivery Journal.[24] We used routinely collected data and nurse reports over one year (2017) from 81 acute medical/surgical units (2178 beds) in four NHS hospital trusts (hereafter referred to as hospitals for brevity) in England. For each unit and for each day we identified staffing measurements: the staffing level deployed (from the electronic roster), the staffing level required (based on patient classifications using the SNCT), and nurses' professional judgement of the completeness of care and adequacy of staffing to deliver quality (through a micro survey embedded in the daily assessments). SNCT and staffing adequacy assessments were provided by the nurse in charge of the shift, hereafter referred to as 'shift leader' for brevity.

For peer review only - http://bmjopen.bmj.com/site/about/guidelines.xhtml

Setting & inclusion

The study sites were one university teaching hospital, two general hospitals and a specialist cancer hospital (two sites) based in London, South East and South West England. The hospitals serve diverse populations including rural areas, deprived inner city populations and specialist national referrals. All hospitals undertook reviews of nurse staffing establishments at least twice a year. Two had been using the SNCT as part of this process for some time while two adopted it shortly before the study commenced.

We included general medical and surgical units that provided 24 hour inpatient care. Services out of scope of the SNCT (e.g. paediatrics, intensive care, maternity, neonatal and palliative care), and any others with highly atypical staffing requirements (e.g. bone marrow transplant and isolation units), as determined by a local co-investigator, were excluded. Our unit sample represented 74% of all beds across the four hospitals.

Data sources and measures

Over the course of one year at least twice per day, shift leaders recorded the number of patients in each SNCT category and made judgements about staffing adequacy (see below) in electronic systems. Local leads trained potential shift leaders on participating units in the use of the SNCT and completion of the staffing adequacy questions. Supporting information and brief guidance was provided on laminated sheets kept near the unit computers where data were entered. Other data for the study were routinely collected for administrative purposes (roster, patient admissions or discharges). Each hospital supplied a profile for each unit with main speciality and layout including the number of beds / single rooms.

For peer review only

For peer review only - http://bmjopen.bmj.com/site/about/guidelines.xhtml

Study variables

We used the most up-to-date SNCT multipliers available at the start of the study.[18] We took the reported counts of patients in each category and calculated the weighted average multiplier per unit and day. We multiplied this by the patient count derived from the patient administration system, in case any patients were omitted from shift leader reports. This figure provides an estimate of the required unit establishment (number of staff to employ). We used morning assessments (substituting later assessments from the same day if missing) and patient count at 7am for our main analysis. The SNCT calculation gives the number of staff to employ (staffing establishment), including an uplift for staff leave and an allowance for sickness, so we converted this into the implied daily hours of staff time available. For this, we used a 37.5 hour working week for 1 whole time equivalent and removed the 22% 'uplift', which is added to the SNCT establishment to account for holidays, study and sick leave. We assumed that this uplift used in the tool was enough for the staff employed to be able to cover all long-term absences.

For each unit we used the average observed skill mix on that unit as a proxy for the planned skill mix of registered nurses and nursing assistants. The SNCT does not directly account for patients identified as requiring 1 to 1 supervision, often referred to as 'specialing' [25] even though the implied staffing requirement is very high. Therefore, where we wanted to identify the staffing requirements for any particular day, we identified the number of such patients from records and added the required hours to our estimated staffing requirement. However, because such enhanced care would form part of the care observed to determine the SNCT multipliers and thus be included in the average, we made no additional allowance when estimating establishments to be employed.

For peer review only - http://bmjopen.bmj.com/site/about/guidelines.xhtml

224 From the electronic roster we identified hours worked by registered nurses and nursing assistants each day (from 7am to 7am) and divided these by the number of patient days (patient hours / 24) to calculate hours per patient day (HPPD) for each unit for each day. We calculated a measure of staff shortfall, by subtracting the required hours (according to the SNCT plus specialing requirements) from the hours actually deployed on that day. If more staff than the estimated requirement were deployed, the shortfall was negative. We also calculated daily patient turnover per staff member (the numbers of patients entering and leaving units divided by the total staff hours).

233 The outcomes measured were a number of variables reflecting the adequacy of nurse staffing, as reported by the nurse in charge of the shift ('shift leader'). The shift leader responded to three brief items every time they provided SNCT ratings, directly inputting responses into the same system as used for SNCT (see Table 1). We chose to use three items for pragmatic reasons, since we judged that more would have been too much of an administrative burden and might have resulted in poorer data quality. Two items, based on the widely used RN4CAST / International Hospital Outcomes surveys of nurse staffing and quality, asked whether there were enough staff for quality and whether any necessary care was left undone. [12, 26] We also asked about staff missing breaks, as nurses may miss breaks to complete care activities, creating additional staff time that avoids adverse effects of staffing shortfalls.[27] These questions constituted the micro-survey.

For peer review only - http://bmjopen.bmj.com/site/about/guidelines.xhtml

Table 1 Staffing adequacy questions

**Questions**
Were there enough nursing staff to provide quality care on the last shift?
Was necessary nursing care left undone (missed) on the last shift because there were too few nursing staff?
Were staff breaks missed on the last shift because there were too few nursing staff?

Data cleaning and Analysis

Data cleaning, processing and statistical analyses were carried out in R statistical software V3.5.0.[28] We identified and removed extreme values of staffing shortfall, where values lay outside the mean plus or minus three standard deviations (approximately 1.5% of cases). This removed atypical periods if the unit was not functioning as normal, e.g. over the Christmas period, or where there is an extreme error in the recorded SNCT ratings. Where there were major changes such as unit moves, changes to the patient population or bed numbers, data for that unit were split and treated as separate units. We found some evidence of consistent reverse coding of data inputs (0/1 for yes/no) for some staffing adequacy questions in several units of one hospital. This appeared to result from erroneous staff training. Because it was discovered partway through the study we developed logical rules to identify units where this occurred and recode data, considering the implications of this through sensitivity analyses where we excluded the hospital entirely.

To understand the accuracy of the estimated establishment we first considered the minimum recommended sample size of the SNCT data collection, which is 20 days twice a year. We used 1000 bootstrap samples of 20 days' data to estimate a mean establishment with a 95% confidence interval for the establishment on each unit. We repeated this with bootstrap samples of increasing numbers of days to assess the accuracy of larger samples. For each unit, we calculated both the precision of the estimate as half the width of the confidence interval expressed as a % of the mean, and the absolute value of the confidence interval in whole time equivalent (WTE)

For peer review only

staff members. We determined the number of units where the confidence interval of the establishment was 2 WTE or less (that is no more than +/-1 WTE difference from the mean) or 1 WTE or less (mean +/- .5).

We modelled the relationship between staffing deficits (in HPPD) and nurse-reported measures of staffing adequacy. We fitted multilevel logistic regression models for binary outcomes using the glmer (generalised linear mixed effects regression) function from the lme4 package [29] in R. Staffing was nested in unit which was nested in hospital. All models included control for day of the week, proportion of single rooms, turnover and unit specialty (surgical vs medical or mixed). We considered the association of staffing adequacy outcomes with deviation of both registered nurse staffing and nursing assistant staffing from their estimated requirements. We also fitted models using the deviation in total hours and skill mix (registered nurse proportion).

After modelling the linear and main effects, we introduced quadratic terms for the staffing level variables to assess non-linear relationships and we investigated whether staffing variables interacted with other variables. We compared the fit of models using the Akaike and Bayesian information criteria (AIC, BIC), preferring models with lower values, indicating better fit / more parsimonious models.[30]

Patient and public involvement

When developing our research proposal, we discussed appropriate patient/public involvement with Claire Ballinger (Patient and Public Involvement lead for the NIHR CLAHRC Wessex) and Anya de Iongh (Patient and Public Involvement champion for the 'fundamental care in hospital' theme of the CLAHRC). Following their guidance we sought no further direct patient / public involvement in prioritising or shaping the

For peer review only - http://bmjopen.bmj.com/site/about/guidelines.xhtml

questions as these arose from the brief and need for technical assessment of the tool, instead we focussed on considering how the public could be involved in the proposed research, its governance and dissemination.

Based on this advice, we sought a lay member of our steering group with specific interest and expertise. To this end, we worked with Stephen Habgood, a lay member of the NICE safe staffing advisory committee for the development of guidelines in mental health, who agreed to participate in the project steering group. Stephen also has experience of staffing methodologies used in other sectors from his past work as a prison governor and is currently chair of a mental health charity. Additionally, and guided by the advice from our patient and public involvement experts, we also considered ward-based staff nurses as the potential end users of our research in a way that is analogous to a patient receiving a treatment that might be recommended by an expert. Based on this we have used multiple channels to connect with ward-based staff including extensive use of social media and discussions with individual staff nurses at consultation events.

Ethical approval and registration

The study was approved by the Health Research Authority through the integrated research application system (IRAS) approval number 190548. Ethical approval was granted by the University of Southampton Ethics committee (reference 18809). The study was prospectively registered (ISRCTN 12307968).[31] This study did not require NHS Research Ethics Committee approval because no data were collected directly from patients, and all patient data were pseudo-anonymised at source with no sensitive patient data transferred.

For peer review only - http://bmjopen.bmj.com/site/about/guidelines.xhtml

Results

We had useable SNCT ratings on 96% of occasions and responses to staffing adequacy questions on 85% or more of possible occasions. After data cleaning and linkage, we had 22,271, 22,294 and 22,364 unit days where we could assess the association between staffing shortfalls and reports of staff breaks missed, nursing care left undone and enough staff for quality respectively.

Average unit staffing levels and skill mix varied considerably between hospitals and between units within hospitals (Table 2). At a hospital level, average estimated staffing requirements of units corresponded closely with the observed staffing levels in 3 of 4 hospitals although all were somewhat understaffed relative to the estimated requirement (8% or less). Larger differences between actual staffing and SNCT estimates occurred on smaller, generally specialist, units with more single rooms (where apparent overstaffing occurred), and some larger medical units (where extremes of apparent understaffing occurred). In hospital C, a specialist hospital with many small units, average unit staffing was 50% higher than the SNCT estimated requirement.

Table 2 Mean and range of unit's average daily staffing levels, skill mix, SNCT estimated staffing requirements.

Hospital	Total hours per patient day			Skill mix (% registered nurses)			Estimated staffing requirement		
	Mean	[Min	Max]	Mean	[Min	Max]	Mean	[Min	Max]
A	7	[5.4	10.4]	51%	[42%	70%]	7.4	5.9	10.2
B	6.8	[5.0	8.9]	56%	[39%	79%]	7.3	6.0	9.4
C	10.5	[7.5	14.2]	75%	[70%	78%]	7	6.3	7.4
D	6.5	[5.2	8.4]	49%	[40%	63%]	7	6.5	7.6
All	7.3	[5.0	8.4]	56%	[39%	79%]	7.2	5.9	10.2

Across all units, using the recommended minimum of 20 days' data, the average precision was 4.1% but varied by unit (range 0.6% - 13.5%). In absolute terms, the average width of the 95% confidence intervals for the establishment was 2.9 whole

For peer review only - http://bmjopen.bmj.com/site/about/guidelines.xhtml

time equivalent staff (that is approximately mean +/- 1.5 WTE). The confidence interval width was ≤2 WTE in 27/86 units and ≤1 WTE in only 3/86.

As the number of days sampled increases from 20 there was a marked increase in precision (Figure 1), with most units (56/86) yielding a confidence interval width of ≤2 WTE from a sample of 40 days. The benefits of increased sample sizes diminishes with larger samples however, and even with samples of 180 days only 53/77 units gave a confidence interval width that was ≤1 WTE wide (Table 3).

Fig 1 Mean precision and confidence interval width of staffing establishment estimates with different sample sizes

Table 3 Average widths of 95% confidence intervals for the mean using different sample sizes to estimate establishment

Sample size taken for the estimate	Average confidence interval width (WTE)	Average precision	Number units with confidence interval width 1 WTE or less	Number units with confidence interval width 2 WTE or less	Number of units[1]
**20**	2.9	4.2%	3	27	86
**40**	2.1	3.0%	7	56	86
**60**	1.7	2.5%	10	64	86
**80**	1.5	2.1%	20	72	86
**100**	1.3	1.9%	31	74	82
**120**	1.2	1.8%	39	74	81
**140**	1.1	1.6%	44	74	81
**160**	1.0	1.5%	50	75	80
**180**	1.0	1.4%	53	73	77

1. Because units with establishment and / or specialty changes were treated as separate units for analysis, the total exceed the number of units participating in the study. As the available data for some units was less than the sample required for the estimate, the number of units for larger samples is reduced.

Across all units, a mean of 78% of shifts were assessed by the nurse in charge as having enough staff to deliver quality care (unit mean range 24% to 96%). Necessary nursing care was reported left undone because of too few staff on 5% of shifts (range 0-25%) and breaks were reported missed on 5% of shifts (range 0-29%).

Shortfalls in staffing levels relative to the requirement for that day, estimated using the SNCT, were associated with nurses' perceptions of staffing adequacy (Table 4).

For peer review only

For peer review only - http://bmjopen.bmj.com/site/about/guidelines.xhtml

In the multivariable models, for each registered nurse hour shortfall, the adjusted odds of the shift leader reporting that there were enough staff for quality were 11% lower, the odds of reporting nursing care left undone were increased by 14%, and the odds of staff missing breaks were increased by 12%. Findings are similar for shortfalls of nursing assistants.

Factors other than shortfalls relative to the SNCT estimated requirement were also associated with perceptions of staffing adequacy. Nurses on surgical units were less likely to perceive adequate staffing compared to nurses on other (medical or mixed) units with lower odds of reporting enough staff for quality and higher odds or reporting care left undone or missed breaks. For example, the odds of nurses reporting that there were enough staff for quality were 46% lower on surgical units. Although relationships were not significant and confidence intervals were wide, the odds of reporting enough staff for quality were substantially lower on units with a higher proportion of single rooms. Similarly, odds of reporting care left undone and missed breaks were substantially increased on units with a higher proportion of single rooms and units with higher turnover, although again confidence intervals were wide and relationships were not statistically significant. Nurses were more likely to report that there were enough staff for quality and less likely to report missed care and missed breaks on Saturday compared to Monday, although there was no consistent pattern that suggested weekends differed from weekdays overall.

For peer review only

Table 4 Association between staffing shortfall and nurse perceptions of staffing adequacy: univariable and multivariable models

	Enough staff for quality				Nursing care left undone				Staff breaks missed			
Variable	odds ratio*	adj odds ratio	95% confidence interval	p-value	odds ratio*	adj odds ratio	95% confidence interval	p-value	odds ratio*	adj odds ratio	95% confidence interval	p-value
Registered nurse shortfall (HPPD)	0.94	0.89	[0.87, 0.92]	0.000	1.09	1.14	[1.08, 1.20]	0.000	1.08	1.12	[1.06, 1.18]	0.000
Nursing assistant shortfall (HPPD)	0.90	0.86	[0.83, 0.89]	0.000	1.08	1.14	[1.07, 1.20]	0.000	1.06	1.11	[1.05, 1.17]	0.000
Turnover (per nursing hour)	0.35	0.91	[0.30, 2.75]	0.863	7.16	3.36	[0.60, 18.72]	0.167	3.85	4.94	[0.95, 25.78]	0.058
Unit type medical or mixed (ref)	1.00	1.00			1.00	1.00			1.00	1.00		

Surgical	0.57	0.54	[0.31, 0.92]	0.023	2.00	2.13	[1.19, 3.82]	0.011	2.13	2.15	[1.23, 3.78]	0.008
Proportion single rooms	0.89	0.54	[0.18, 1.66]	0.283	1.58	3.01	[0.93, 9.71]	0.065	1.05	2.06	[0.65, 6.56]	0.221
Day of week- Monday (ref)	1.00	1.00			1.00	1.00			1.00	1.00		
Tuesday	1.11	1.12	[0.99, 1.26]	0.079	0.86	0.86	[0.69, 1.06]	0.160	0.94	0.71	[0.58, 0.88]	0.001
Wednesday	1.28	1.28	[1.13, 1.45]	0.000	0.95	0.96	[0.78, 1.18]	0.684	1.01	0.61	[0.49, 0.76]	0.000
Thursday	1.09	1.08	[0.96, 1.23]	0.200	0.90	0.91	[0.73, 1.13]	0.383	0.96	0.81	[0.66, 1.00]	0.045
Friday	1.03	1.03	[0.91, 1.17]	0.610	0.93	0.93	[0.74, 1.15]	0.488	1.03	0.79	[0.64, 0.97]	0.028
Saturday	1.28	1.29	[1.14, 1.47]	0.000	0.74	0.75	[0.60, 0.95]	0.016	0.86	0.50	[0.40, 0.64]	0.000
Sunday	1.02	1.02	[0.90, 1.15]	0.811	1.08	1.11	[0.90, 1.37]	0.310	0.84	0.82	[0.66, 1.01]	0.058
Variance partition coefficient for units**		0.22				0.22				0.23		
Variance partition coefficient for hospitals**		0.12				0.17				0.11		
Akaike information criterion		20697				8377				8094		
Bayesian information criterion		20809				8490				8206		

Ref – reference category for categorical variables. *Odds ratio derived from entering this variable only into the multilevel model. Calculated as between-group residual variance divided by total variance using the latent variable approach [32]

For peer review only

For peer review only - http://bmjopen.bmj.com/site/about/guidelines.xhtml

We tested for non-linear relationships with registered nurse and nursing assistant shortfalls, as would be expected if the SNCT provides a threshold for adequate staffing. We estimated models using effects that were significant in the main effects models, adding a variable for staffing shortfall squared for each staff group. For nursing care left undone, the non-linear term for registered nurse staffing was significant but there was no clear indication that the model was preferable (AIC Δ-2, BIC Δ +14) and the overall relationship was not changed substantially. No indication of a threshold where benefit / harm starts could be observed (see Fig 2). For other outcomes, non-linear terms were not significant and associated with increased AIC & BIC (Supplementary Table 1).

FIG 2 Change in odds of reporting care left undone with change in staffing shortfalls estimated from model with non-linear staffing effects

We estimated models that included all statistically significant variables and interactions between staffing shortfall and these variables. There were no significant interaction effects between registered nurse shortfall and nursing assistant shortfall and both AIC and BIC increased for these models (Supplementary Table 2), so the simpler models were preferred.

Models using overall care hour shortfall (registered nurse and assistants) per patient day gave similar coefficients, with each care hour per patient day of shortfall associated with a 12% reduction in the odds of reporting enough staff for quality and no significant associations with skill mix (Supplementary Table 3). Because of the coding errors noted from units in one of the hospitals, we repeated the main models omitting data from this hospital. Results were largely unchanged with no effect on substantive conclusions (see Supplementary Table 4 for an example).

For peer review only - http://bmjopen.bmj.com/site/about/guidelines.xhtml

Discussion

This study reports the first independent assessment of the SNCT, which is widely used to determine staffing levels in English hospitals. Using the recommended minimum 20 day sample, estimates for the number of nurses that should be employed on a ward had an average precision of 4.2%, but wide confidence intervals for the absolute staff numbers needed. A sample of 40 days gave an estimate within +/- 1 staff members for the majority of wards but much larger samples (100 days or more) are required to estimate the staff required with a confidence interval width no more than 1 staff member wide in the majority of wards. When staffing shortfalls were high, relative to the required level estimated using the SNCT for that day, staff were less likely to report that they had enough staff for quality and more likely to report that necessary nursing care was omitted and staff breaks missed. These relationships appeared to be linear, with no threshold when staffing reached the SNCT recommended level. Other factors, not included in the patient classifications used by the SNCT, including unit speciality and day of the week are also associated with whether a given staffing level is deemed to be sufficient by nurses working on the unit.

The original purpose of the SNCT was to ensure that units employed sufficient nurses to be able to provide the care hours required by patients. Existing reports attest to the inter-rater reliability of the tool [19, 20] but the recommendation that this staffing establishment is estimated using at least 20 days of SNCT data recognises that daily demand is variable and estimates based on small samples may be imprecise. The average level of precision achieved from 20 days of observations in our study appears, superficially, to be acceptable. However, this masks considerable

For peer review only - http://bmjopen.bmj.com/site/about/guidelines.xhtml

430 variation in precision between units and large absolute differences in terms of
431 numbers of staff. Using a conventional (if technically slightly inaccurate)
432 interpretation of the confidence interval, this means that for many units, estimates
433 could vary from the true staffing requirement by more than 2 whole staff members.
434 The absolute importance of such differences may vary by unit, but the potential
435 significance of such inaccuracy is great.

436 Small increases in the number of days used to estimate establishments yield
437 substantial improvements in precision, although there are rapidly diminishing returns
438 from samples of more than 40 days. As more and more hospitals are gathering
439 SNCT ratings on a daily basis it may be that these data could be drawn on to review
440 establishments, with the resources currently used to provide periodic review invested
441 instead into quality control for the unit reports. Moving averages could be substituted
442 for intermittent review, with statistical process control methods used to determine
443 when changes in demand sufficient to revise the establishment have occurred [33].

444 For some units, variation is such that the estimated establishment may always be
445 imprecisely estimated and our results also highlight that unmeasured influences on
446 demand arising from factors such as unit layout and speciality could have a
447 substantial influence on the staffing requirements. In these cases, the use of
448 professional judgement, already emphasised within the guidance for the SNCT, is
449 paramount. In the face of an apparently objective measure, it is easy to prioritise the
450 measured quantity despite the substantial uncertainty associated with it. [34] It is clear
451 from both these results and the wider literature that professional judgement remains
452 an essential element in determining the required level of nurse staffing. [3, 4]

For peer review only - http://bmjopen.bmj.com/site/about/guidelines.xhtml

Although our results about other influences on workload were imprecise, both turnover and single rooms have been identified in other research as factors that increase nursing workload,[21-23, 35, 36] because of the specific work associated with admissions and discharge, increases in indirect care requirements and, for single rooms, the need for increased surveillance of potentially vulnerable patients. The increased workload associated with turnover is acknowledged in the SNCT, where revised multipliers are provided specific to acute admissions units, to reflect the high patient turnover.[18] Our findings could arise because variation in turnover within and between general units is not being sufficiently accommodated within the average demand across all patients. Our finding that nurses on surgical units were less likely to perceive adequate staffing compared to nurses on other (medical or mixed) units could occur if surgical units had a higher workload for a given level of acuity / dependency, which may also result from indirect care associated with surgery, such as arranging transports and providing escorts.[37] This is a novel finding, and where recommendations or mandates for minimum staffing levels exist, medical units are not generally differentiated from surgical units.[38]

While there must be a balance between parsimony and accuracy in any tool these findings could arise because the current SNCT staffing recommendations fit some units better than others. This lack of fit might be remedied by tailoring the tool and creating versions specific to particular circumstances. Further revisions to the SNCT recommended staffing levels, represented by different “multipliers” for different unit types beyond the current admissions unit specific multipliers, require further investigation. The wide confidence intervals associated with single rooms and turnover do not directly lend themselves to a formal revision of the multipliers, but the

For peer review only - http://bmjopen.bmj.com/site/about/guidelines.xhtml

importance of exercising professional judgments about other factors affecting workload is clear.

Although the SNCT multipliers were originally derived using professional expert estimates of time required, subsequent developments have used empirical observations to revise the multipliers.[20] Our study is the first to show that staffing shortfalls, relative to requirement estimated using the tool are associated with professional judgements that staffing is insufficient to maintain quality care and other indicators that staffing may be inadequate. However, if the SNCT were indicating a level of staffing that was generally judged sufficient to meet all care needs with quality, the relationship between shortfall and staffing adequacy would be expected to diminish as staffing levels increase above the recommended level. Instead the relationships we observed were essentially linear, with no evidence of a threshold above which additional staffing had little effect on the likelihood that nurses would report that there were enough staff. A recent study using the RAFAELA system, widely used in Northern Europe, gave a similar finding. Staffing above the level defined as 'optimal' by the system was associated with decreases in mortality[39, 40]. A recent study found that staffing below establishment as determined using the SNCT was associated with an increased risk of death in hospital [11, 23] but for registered nurses staffing the relationship was linear, with no threshold. So while our findings are consistent with the SNCT providing a measure of demand, there is no evidence to support the assertion that the recommended staffing levels are optimal in any meaningful sense.

The effects of registered nurse and nurse assistant shortfalls on perceptions of staffing adequacy were similar but independent. Although such a finding might be

For peer review only - http://bmjopen.bmj.com/site/about/guidelines.xhtml

interpreted as indicating that there is substitution between registered nurses and assistants, the contribution of the two groups to quality and safety is not equivalent they are not interchangeable. A large body of research points to the specific importance of maintaining a rich registered nurse skill mix for patient safety. [41] More recent studies have shown the important contribution of both registered nurses and assistants in maintaining both patient safety and the quality of interpersonal care. [11, 42, 43] Simple substitutions are not feasible because the contributions of each group are distinct. Effective deployment of assistants is contingent on having sufficient RNs to supervise and support them. [11, 42]

The SNCT, while widely used in England is by no means the only staffing tool available. Given the vast numbers of reports and different tools it is hard to say definitively that there are no data that would allow comparison of the precision of the SNCT with other tools for estimating staffing establishments, but our reviews found no recent studies giving similar data about other tools. [3, 4]

Limitations

Training was provided to unit nurses in using the SNCT but the extensive nature of the study is such that the reliability of the ratings we used is likely to be less than that achieved by expert raters in a dedicated review of establishments. However, the wide variation in precision of estimates of unit establishments is unlikely to be explained by this factor alone. Furthermore, the circumstances of our study resemble routine use of the system when, as is now becoming common, assessments are completed daily by shift leaders. We did find evidence of systematic coding errors for the assessment of staffing adequacy on some units, although our substantive conclusions were unaffected. However, this may indicate that less systematic errors

For peer review only - http://bmjopen.bmj.com/site/about/guidelines.xhtml

were also occurring. The effect of such errors would be to attenuate our ability to estimate relationships and so we may be underestimating the relationship between staffing shortfalls and perceptions of staffing adequacy. We investigated correlations and causality cannot be assumed. Our study did not explore the relationship between staffing and objective care outcomes; instead our measures of staffing adequacy relied on subjective reports by nurses. However, these subjective assessments have been shown to be associated with important patient outcomes [44-46]. We did not ask staff to report consequences of overstaffing. In our study the judgement of staffing adequacy and ratings of the SNCT were performed by the same people, so it is possible there is some degree of bias. However, the nature of the SNCT reports – numbers of patients in each category – is sufficiently distinct from the staffing adequacy questions that our findings are unlikely to be a simple product of common method bias. Our sample was large but arose from only four hospitals so we cannot be sure that these results would generalise across all hospitals.

Conclusions

In this study we have asked (and answered) a number of questions about the SNCT. These questions: the precision with which establishments are estimated, the extent to which the averages provided from a patient classification can accommodate variation from factors that are unrelated to individual patients and whether the identified staffing level is in any sense “optimal”, all need to be asked of other tools. Our recent reviews suggest such questions are rarely asked of other tools and are even more rarely answered.

For peer review only

For peer review only - http://bmjopen.bmj.com/site/about/guidelines.xhtml

The SNCT can provide a reliable estimate of a unit staffing establishment but larger samples than the currently recommended minimum are required for most units to provide estimates that are within 1 whole time equivalent staff member of the mean. For some units, such precision is hard to obtain and there may be systematic variations in staffing requirements associated with some unit types that are not accounted for by the SNCT. While we recommend further exploration of the factors affecting the reliability and validity of the SNCT estimates, and suggest that moving averages instead of periodic reassessments could be used to identify when changes in establishments are needed, our findings also firmly underpin the conclusion that measurement is an adjunct to professional judgements, not a replacement for it. The SNCT does appear to provide a measure of nursing workload but the recommended staffing levels derived from it are not necessarily optimal.

[words 5254]

For peer review only

For peer review only - http://bmjopen.bmj.com/site/about/guidelines.xhtml

Acknowledgements

The authors of this paper wish to acknowledge the contributions of Rosemary Chable, Andrew Dimech, Jeremy Jones, Yvonne Jeffrey, Antonello Maruotti, Alejandra Recio Saucedo, and Nicola Sinden who contributed to the acquisition of funding and the design of the project; and Clare Aspden, Tracey Cassar and Shirley Hunter who contributed to data collection. We also wish to acknowledge all the nurses who answered staffing adequacy questions and completed SNCT ratings.

Author statement

Peter Griffiths (Professor, Health Services Research) Principal Investigator, original conceptualisation of the study and study design, secured funding, oversaw acquisition of the data, data analysis, interpretation of results and drafted article.

Christina Saville (Research Fellow, Operational Research) undertook descriptive and regression analyses and contributed to critical revision of the article.

Jane Ball (Professor, Health Services Research) contributed to study design, acquisition of funding, interpretation of results and critical revision of the article.

David Culliford (Senior Medical Statistician) provided statistical advice, contributed to interpretation of results and critical revision of the article.

Natalie Pattison (Clinical Professor, Nursing) contributed to study design, acquisition of funding, acquisition of the data, interpretation of results and critical revision of the article.

Thomas Monks (Principal Research Fellow, Operational Research and Data Science) contributed to study design, acquisition of funding, oversaw acquisition of the data, data analysis, interpretation of results and critical revision of the article.

Funding statement

This report presents independent research funded by the United Kingdom's National Institute for Health Research (NIHR) Health Services and Delivery Research Programme number 14/194/21. JEB, NP, PG and TM were award holders. The funders had no role in study design, data collection and analysis, decision to publish, or preparation of the manuscript. The views and opinions expressed by authors in this publication are those of the authors and do not necessarily reflect those of the

For peer review only - http://bmjopen.bmj.com/site/about/guidelines.xhtml

NHS, the NIHR, NETSCC, the PHR programme or the Department of Health and Social Care.

Competing interests

Peter Griffiths is a member of the National Health Service Improvement (NHSI) safe staffing faculty steering group. The safe staffing faculty programme is intended to ensure that knowledge of the Safer Nursing Care Tool (SNCT), its development and its operational application is consistently applied across the NHS.

Data sharing statement

All data supporting this article are openly available from the University of Southampton repository at https://doi.org/10.5258/SOTON/D1134.

For peer review only

For peer review only - http://bmjopen.bmj.com/site/about/guidelines.xhtml

References

1. Kavanagh KT, Cimiotti JP, Abusalem S, et al. Moving healthcare quality forward with nursing-sensitive value-based purchasing. *J Nurs Scholarsh* 2012; 44: 385-395.

2. Griffiths P, Ball J, Drennan J, et al. Nurse staffing and patient outcomes: Strengths and limitations of the evidence to inform policy and practice. A review and discussion paper based on evidence reviewed for the National Institute for Health and Care Excellence Safe Staffing guideline development. *Int J Nurs Stud* 2016; 63: 213-225.

3. Saville CE, Griffiths P, Ball JE, et al. How many nurses do we need? A review and discussion of operational research techniques applied to nurse staffing. *Int J Nurs Stud* 2019; 97: 7-13.

4. Griffiths P, Saville C, Ball J, et al. Nursing workload, Nurse Staffing Methodologies & Tools: a systematic scoping review & discussion *Int J Nurs Stud* 2020; 103

5. Ball J, Barker H, Griffiths P, et al. *Implementation, Impact and Costs of Policies for Safe Staffing In Acute Trusts: Report to Funders:*. 2019. Southampton: University of Southampton.

6. National Institute for Health and Clinical Excellence. *Safer Nursing Care Tool (SNCT): statement of endorsement*. 2014.

7. Griffiths P, Recio-Saucedo A, Dall'Ora C, et al. The association between nurse staffing and omissions in nursing care: A systematic review. *J Adv Nurs* 2018; 74: 1474-1487.

8. Kane RL, Shamliyan TA, Mueller C, et al. The Association of Registered Nurse Staffing Levels and Patient Outcomes: Systematic Review and Meta-Analysis. *Med Care* 2007; 45: 1195-1204.

9. Shekelle PG. Nurse–Patient Ratios as a Patient Safety Strategy: A Systematic Review. *Ann Intern Med* 2013; 158: 404-409.

10. Aiken LH, Sloane DM, Bruyneel L, et al. Nurse staffing and education and hospital mortality in nine European countries: a retrospective observational study. *Lancet* 2014; 383: 1824-1830.

11. Griffiths P, Ball J, Bloor K, et al. Nurse staffing levels, missed vital signs and mortality in hospitals: retrospective longitudinal observational study. *Health Services and Delivery Research Journal* 2018; 6: 38.

12. Ball JE, Murrells T, Rafferty AM, et al. 'Care left undone' during nursing shifts: associations with workload and perceived quality of care. *BMJ Qual Saf* 2014; 23: 116-125.

13. Zander-Jentsch B WF, Rzayeva N, et al. Germany. In: Rafferty AM BR, Zander-Jentsch B, et al. (ed) *Strengthening health systems through nursing: Evidence from 14 European countries [Internet]*. Copenhagen (Denmark): European Observatory on Health Systems and Policies.

14. Leary A, Cook R, Jones S, et al. Mining routinely collected acute data to reveal non-linear relationships between nurse staffing levels and outcomes. *BMJ Open* 2016; 6: e011177.

15. Pitkaaaho T, Partanen P, Miettinen M, et al. Non-linear relationships between nurse staffing and patients’ length of stay in acute care units: Bayesian dependence modelling. *J Adv Nurs* 2015; 71: 458–473.

16. Hoi SY, Ismail N, Ong LC, et al. Determining nurse staffing needs: the workload intensity measurement system. *J Nurs Manag* 2010; 18: 44-53.

For peer review only - http://bmjopen.bmj.com/site/about/guidelines.xhtml

17. Buchan J. A certain ratio? The policy implications of minimum staffing ratios in nursing. *J Health Serv Res Policy* 2005; 10: 239-244.

18. The Shelford group. *Safer Nursing Care Tool Implementation Resource Pack*. 2014. The Shelford Group.

19. Hurst K, Smith A, Casey A, et al. Calculating staffing requirements. *Nurs Manag (Harrow)* 2008; 15: 26-34.

20. Smith S, Casey A, Hurst K, et al. Developing, testing and applying instruments for measuring rising dependency-acuity's impact on ward staffing and quality. *Int J Health Care Qual Assur* 2009; 22: 30-39.

21. Hurst K. UK ward design: Patient dependency, nursing workload, staffing and quality--An observational study. *Int J Nurs Stud* 2008; 45: 370-381.

22. Maben J, Griffiths P, Penfold C, et al. One size fits all? Mixed methods evaluation of the impact of 100% single-room accommodation on staff and patient experience, safety and costs. *BMJ Qual Saf* 2016; 25: 241-256.

23. Griffiths P, Maruotti A, Recio Saucedo A, et al. Nurse staffing, nursing assistants and hospital mortality: retrospective longitudinal cohort study. *BMJ Qual Saf* 2019; 28: 609-617.

24. Griffiths P, Saville C, Ball JE, et al. The Safer Nursing Care Tool as a guide to nurse staffing requirements on hospital wards: observational and modelling study. *Health Services and Delivery Research* 2020; 8: 1-161.

25. Wood VJ, Vindrola-Padros C, Swart N, et al. One to one specialling and sitters in acute care hospitals: A scoping review. *Int J Nurs Stud* 2018; 84: 61-77.

26. Sermeus W, Aiken LH, Van den Heede K, et al. Nurse forecasting in Europe (RN4CAST): Rationale, design and methodology. *BMC Nurs* 2011; 10: 6.

27. Patterson J. The effects of nurse to patient ratios. *Nurs Times* 2011; 107: 22-25.

28. R Core Team. *R: A Language and Environment for Statistical Computing*. v3.5.0 ed. Vienna, Austria: R Foundation for Statistical Computing, 2019.

29. Bates D, Mächler M, Bolker B, et al. Fitting Linear Mixed-Effects Models Usinglme4. *Journal of Statistical Software* 2015; 67: 1-48.

30. Burnham KP and Anderson DR. Multimodel Inference. *Sociological Methods & Research* 2016; 33: 261-304.

31. Griffiths P. Using the Safer Nursing Care Tool to identify nurse staffing requirements in hospitals. *ISRTCN* 2016; https://doi.org/10.1186/ISRCTN12307968

32. Goldstein H, Browne W and Rasbash J. Partitioning Variation in Multilevel Models. *Understanding Statistics* 2002; 1: 223-231.

33. Wong H, Gan F and Chang T. Designs of moving average control chart. *Journal of Statistical Computation and Simulation* 2004; 74: 47-62.

34. Downie R and Macnaughton J. In Defence of Professional Judgement. *Adv Psychiatr Treat* 2013; 15: 322-327.

35. Needleman J, Buerhaus P, Pankratz VS, et al. Nurse staffing and inpatient hospital mortality. *N Engl J Med* 2011; 364: 1037-1045.

36. Myny D, Van Hecke A, De Bacquer D, et al. Determining a set of measurable and relevant factors affecting nursing workload in the acute care hospital setting: a cross-sectional study. *Int J Nurs Stud* 2012; 49: 427-436.

37. Myny D, Van Goubergen D, Gobert M, et al. Non-direct patient care factors influencing nursing workload: a review of the literature. *J Adv Nurs* 2011; 67: 2109-2129.

For peer review only - http://bmjopen.bmj.com/site/about/guidelines.xhtml

38. Royal College of Nursing. *Policy Briefing: Mandatory Nurse Staffing levels*. 2012. London: RCN.

39. Fagerstrom L, Kinnunen M and Saarela J. Nursing workload, patient safety incidents and mortality: an observational study from Finland. *BMJ Open* 2018; 8: e016367.

40. Junttila JK, Koivu A, Fagerström L, et al. Hospital mortality and optimality of nursing workload: A study on the predictive validity of the RAFAELA Nursing Intensity and Staffing system. *Int J Nurs Stud* 2016; 60: 46-53.

41. Aiken LH, Sloane D, Griffiths P, et al. Nursing skill mix in European hospitals: cross-sectional study of the association with mortality, patient ratings, and quality of care. *BMJ Qual Saf* 2017; 26: 559-568.

42. Bridges J, Griffiths P, Oliver E, et al. Hospital nurse staffing and staff-patient interactions: an observational study. *BMJ Qual Saf* 2019; 28: 706-713.

43. Shang J, Needleman J, Liu J, et al. Nurse Staffing and Healthcare-Associated Infection, Unit-Level Analysis. *J Nurs Adm* 2019; 49: 260-265.

44. Ball JE, Bruyneel L, Aiken LH, et al. Post-operative mortality, missed care and nurse staffing in nine countries: A cross-sectional study. *Int J Nurs Stud* 2018; 78: 10-15.

45. Recio-Saucedo A, Dall'Ora C, Maruotti A, et al. What impact does nursing care left undone have on patient outcomes? Review of the literature. *J Clin Nurs* 2018; 27: 2248-2259.

46. Bruyneel L, Li B, Ausserhofer D, et al. Organization of Hospital Nursing, Provision of Nursing Care, and Patient Experiences With Care in Europe. *Med Care Res Rev* 2015; 72: 643-664.

For peer review only

For peer review only - http://bmjopen.bmj.com/site/about/guidelines.xhtml

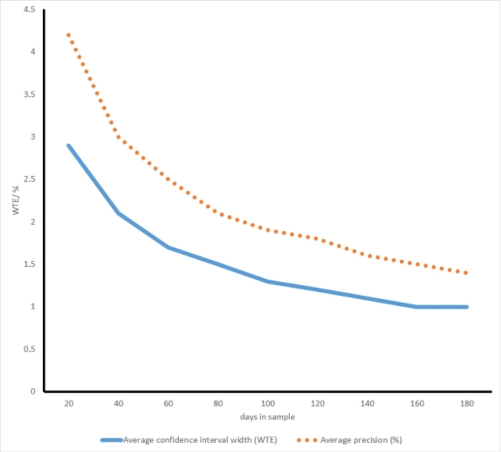

Mean precision and confidence interval width of staffing establishment estimates with different sample sizes

146x82mm (300 x 300 DPI)

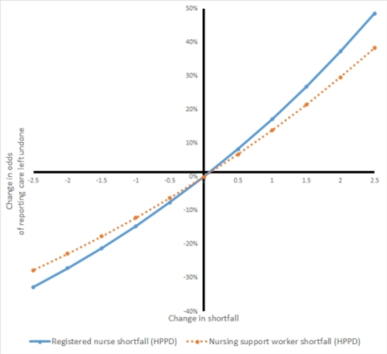

Change in odds of reporting care left undone with change in staffing shortfalls estimated from model with non-linear staffing effects

304x171mm (144 x 144 DPI)

Supplementary tables

Table 1 Association between staffing shortfall and nurse perceptions of staffing adequacy: test for non-linear staffing effects in multivariable models.

	ENOUGH STAFF FOR QUALITY			NURSING CARE LEFT UNDONE			STAFF BREAKS MISSED		
**VARIABLE**	Odds ratio	95% confidence interval	p-value	Odds ratio	95% confidence interval	p-value	Odds ratio	95% confidence interval	p-value
**REGISTERED NURSE SHORTFALL (HPPD)**	0.89	[0.86, 0.92]	**<0.001**	1.15	[1.09, 1.21]	**<0.001**	1.13	[1.08, 1.19]	**<0.001**
**NURSING SUPPORT WORKER SHORTFALL (HPPD)**	0.85	[0.83, 0.88]	**<0.001**	1.15	[1.08, 1.22]	**<0.001**	1.12	[1.06, 1.19]	**<0.001**
**REGISTERED NURSE SHORTFALL SQUARED**	1.00	[0.99, 1.00]	0.469	1.01	[1.00, 1.02]	**0.021**	1.00	[0.99, 1.02]	0.533
**NURSING SUPPORT WORKER SHORTFALL SQUARED**	1.01	[1.00, 1.02]	0.102	0.99	[0.97, 1.01]	0.194	0.99	[0.97, 1.01]	0.165
**DAY OF WEEK- MONDAY (REFERENCE)**									
**TUESDAY**							0.71	[0.58, 0.88]	**0.002**
**WEDNESDAY**							0.61	[0.49, 0.75]	**<0.001**
**THURSDAY**							0.82	[0.66, 1.00]	0.053
**FRIDAY**							0.80	[0.65, 0.99]	**0.042**
**SATURDAY**							0.49	[0.39, 0.62]	**<0.001**
**SUNDAY**							0.80	[0.65, 0.98]	**0.031**
**WARD TYPE- OTHER (REFERENCE)**									
**SURGICAL**	0.57	[0.33, 0.96]	**0.036**	1.98	[1.10, 3.54]	**0.022**	2.07	[1.18, 3.63]	**0.011**
**PROPORTION SINGLE ROOMS**									
**VARIANCE PARTITION COEFFICIENT FOR UNITS**	0.22			0.21			0.23		
**VARIANCE PARTITION COEFFICIENT FOR HOSPITALS**	0.15			0.20			0.13		
**AKAIKE INFORMATION CRITERION**	20715			8378			8097		
**BAYESIAN INFORMATION CRITERION**	20779			8442			8209		

For peer review only

For peer review only - http://bmjopen.bmj.com/site/about/guidelines.xhtml

Table 2 Association between staffing shortfall and nurse perceptions of staffing adequacy: test for interactions between significant variables in multivariable models

	Enough staff for quality			Nursing care left undone			Staff breaks Missed		
**Variable**	**Odds ratio**	**95% confidence interval**	**p-value**	**Odds ratio**	**95% confidence interval**	**p-value**	**Odds ratio**	**95% confidence interval**	**p-value**
**Registered nurse shortfall (HPPD)**	0.92	[0.89, 0.95]	**<0.001**	1.14	[1.07, 1.21]	**<0.001**	1.13	[1.02, 1.25]	**0.019**
**Nursing support worker shortfall (HPPD)**	0.87	[0.84, 0.90]	**<0.001**	1.14	[1.06, 1.22]	**<0.001**	1.13	[1.00, 1.28]	**0.049**
**Registered nurse shortfall squared (HPPD)**				1.01	[1.00, 1.02]	0.044			
**Day of week- Monday (reference)**									
**Tuesday**							0.71	[0.57, 0.90]	**0.005**
**Wednesday**							0.60	[0.47, 0.76]	**<0.001**
**Thursday**							0.84	[0.67, 1.05]	0.135
**Friday**							0.87	[0.69, 1.09]	0.225
**Saturday**							0.50	[0.38, 0.65]	**<0.001**
**Sunday**							0.81	[0.64, 1.01]	0.064
**Unit type- Other (reference)**									
**Surgical**	0.63	[0.37, 1.08]	0.096	2.03	[1.11, 3.69]	**0.021**	2.04	[1.15, 3.63]	**0.015**
**Proportion single rooms**									
**Turnover (per nursing hour)**									
**Registered nurse shortfall : Nursing support worker shortfall**	0.99	[0.98, 1.01]	0.315	1.00	[0.98, 1.02]	0.972	1.01	[0.99, 1.03]	0.202
**Registered nurse shortfall : Tuesday**							0.98	[0.86, 1.11]	0.708
**Registered nurse shortfall : Wednesday**							0.99	[0.86, 1.13]	0.840
**Registered nurse shortfall : Thursday**							0.96	[0.84, 1.09]	0.540
**Registered nurse shortfall : Friday**							0.94	[0.82, 1.07]	0.332
**Registered nurse shortfall : Saturday**							1.05	[0.89, 1.24]	0.558
**Registered nurse shortfall : Sunday**							1.04	[0.91, 1.20]	0.566
**Registered nurse shortfall : Surgical unit**	0.88	[0.82, 0.95]	**0.001**	1.00	[0.89, 1.13]	0.952	1.00	[0.89, 1.12]	0.961
**Registered nurse shortfall: Proportion single rooms**									

For peer review only

	Enough staff for quality			Nursing care left undone			Staff breaks Missed		
**Variable**	**Odds ratio**	**95% confidence interval**	**p-value**	**Odds ratio**	**95% confidence interval**	**p-value**	**Odds ratio**	**95% confidence interval**	**p-value**
**Nursing support worker shortfall: Tuesday**							1.01	[0.85, 1.20]	0.905
**Nursing support worker shortfall: Wed**							1.04	[0.87, 1.24]	0.694
**Nursing support worker shortfall: Thursday**							0.95	[0.81, 1.11]	0.536
**Nursing support worker shortfall: Friday**							0.87	[0.74, 1.02]	0.097
**Nursing support worker shortfall: Saturday**							0.92	[0.76, 1.11]	0.390
**Nursing support worker shortfall: Sunday**							0.95	[0.80, 1.12]	0.522
**Nursing support worker shortfall: Surgical unit**	0.95	[0.88, 1.04]	0.263	0.98	[0.86, 1.11]	0.766	1.07	[0.94, 1.21]	0.325
**Nursing support worker shortfall: Proportion single rooms**									
Variance partition coefficient for units	0.22			0.21			0.23		
Variance partition coefficient for hospitals	0.14			0.20			0.13		
**Akaike information criterion**	20708			8384			8116		
**Bayesian information criterion**	20780			8464			8341		

For peer review only

For peer review only - http://bmjopen.bmj.com/site/about/guidelines.xhtml

Table 3: Association between overall (registered nurse and assistant) shortfall and nurse perceptions of staffing adequacy

	Enough staff for quality			Nursing care left undone			Staff breaks missed		
**Variable**	Odds ratio	95% confidence interval	p-value	Odds ratio	95% confidence interval	p-value	Odds ratio	95% confidence interval	p-value
**Overall shortfall (HPPD)**	0.88	[0.86, 0.90]	**<0.001**	1.14	[1.09, 1.19]	**<0.001**	1.11	[1.07, 1.16]	**<0.001**
**Skill mix (registered nurse HPPD/ total HPPD)**	0.81	[0.62, 1.06]	0.117	0.74	[0.47, 1.17]	0.195	0.88	[0.57, 1.38]	0.586
**Patient turnover (per care hour)**	0.91	[0.30, 2.77]	0.869	3.51	[0.63, 19.65]	0.153	4.99	[0.96, 26.02]	0.057
**Unit type- other (reference)**									
**Surgical**	0.54	[0.31, 0.93]	**0.025**	2.14	[1.20, 3.83]	**0.010**	2.16	[1.23, 3.80]	**0.007**
**Proportion single rooms**	0.53	[0.17, 1.64]	0.271	3.01	[0.93, 9.69]	0.065	2.06	[0.65, 6.57]	0.222
**Day of week- Monday (reference)**									
**Tuesday**	1.12	[0.99, 1.26]	0.078	0.86	[0.69, 1.06]	0.159	0.71	[0.58, 0.88]	**0.001**
**Wednesday**	1.28	[1.13, 1.45]	**<0.001**	0.96	[0.77, 1.18]	0.676	0.61	[0.49, 0.76]	**<0.001**
**Thursday**	1.09	[0.96, 1.23]	0.195	0.91	[0.73, 1.13]	0.379	0.81	[0.66, 1.00]	**0.045**
**Friday**	1.03	[0.91, 1.17]	0.604	0.92	[0.74, 1.15]	0.481	0.79	[0.64, 0.97]	**0.028**
**Saturday**	1.30	[1.14, 1.48]	**<0.001**	0.75	[0.60, 0.95]	0.016	0.50	[0.40, 0.64]	**<0.001**
**Sunday**	1.02	[0.90, 1.15]	0.795	1.11	[0.90, 1.37]	0.316	0.82	[0.66, 1.01]	0.058
Variance partition coefficient for units	0.22			0.22			0.23		
Variance partition coefficient for hospitals	0.12			0.17			0.11		
**Akaike information criterion**	20700			8376			8094		
**Bayesian information criterion**	20812			8488			8206		

For peer review only

For peer review only - http://bmjopen.bmj.com/site/about/guidelines.xhtml

Performance of the Safer Nursing Care Tool

Table 4: Association between staffing shortfall and nurse perceptions of "enough staff for quality": comparison of coefficients from models with full sample vs model excluding the Trust where recoding occurred.

	Model with full sample			MODEL EXCLUDING 'HOSPITAL D'		
**Variable**	ADJ ODDS RATIO	95% CONFIDENCE INTERVAL	P-VALUE	ADJ ODDS RATIO	95% CONFIDENCE INTERVAL	P-VALUE
**Registered nurse shortfall (HPPD)**	0.89	[0.87, 0.92]	**<0.001**	0.89	[0.86, 0.91]	**<0.001**
**Nursing support worker shortfall (HPPD)**	0.86	[0.83, 0.89]	**<0.001**	0.84	[0.81, 0.87]	**<0.001**
**Turnover (per care hour)**	0.91	[0.30, 2.75]	0.863	0.83	[0.26, 2.63]	0.747
**Ward type – other (reference)**						
**Surgical**	0.54	[0.31, 0.92]	**0.023**	0.45	[0.27, 0.76]	**0.003**
**Proportion single rooms**	0.54	[0.18, 1.66]	0.283	0.61	[0.24, 1.57]	0.304
**Day of week- Monday (reference)**						
**Tuesday**	1.12	[0.99, 1.26]	0.079	1.20	[1.05, 1.37]	**0.008**
**Wednesday**	1.28	[1.13, 1.45]	**<0.001**	1.31	[1.15, 1.50]	**<0.001**
**Thursday**	1.08	[0.96, 1.23]	0.2000	1.13	[0.99, 1.29]	**0.071**
**Friday**	1.03	[0.91, 1.17]	0.610	1.08	[0.94, 1.24]	0.278
**Saturday**	1.29	[1.14, 1.47]	**<0.001**	1.40	[1.21, 1.61]	**<0.001**
**Sunday**	1.02	[0.90, 1.15]	0.811	1.03	[0.90, 1.17]	0.697

For peer review only - http://bmjopen.bmj.com/site/about/guidelines.xhtml

The RECORD statement – checklist of items, extended from the STROBE statement, that should be reported in observational studies using routinely collected health data.

	Item No.	STROBE items	Location in manuscript where items are reported	RECORD items	Location in manuscript where items are reported
**Title and abstract**					
	1	(a) Indicate the study's design with a commonly used term in the title or the abstract (b) Provide in the abstract an informative and balanced summary of what was done and what was found	a) Pg 1 Title "observational"<br>b) Pg 1 Abstract	RECORD 1.1: The type of data used should be specified in the title or abstract. When possible, the name of the databases used should be included.<br><br>RECORD 1.2: If applicable, the geographic region and timeframe within which the study took place should be reported in the title or abstract.<br><br>RECORD 1.3: If linkage between databases was conducted for the study, this should be clearly stated in the title or abstract.	1.1 "SNCT ratings" and "staffing adequacy questions" pg 1 Abstract<br>1.2 Not enough words. This is reported in the Methods pg 8 line 158-160<br>1.3 Pg 1 Abstract Design
**Introduction**					
Background rationale	2	Explain the scientific background and rationale for the investigation being reported	2. Pg 5-7		

For peer review only

Objectives	3	State specific objectives, including any prespecified hypotheses	Pg 7 Line 146 - 156		
**Methods**					
Study Design	4	Present key elements of study design early in the paper	Pg 7 line 146 "This observational study". Pg 8 line 158 "We used routinely-collected data and nurse reports…"		
Setting	5	Describe the setting, locations, and relevant dates, including periods of recruitment, exposure, follow-up, and data collection	See Methods Pg 158-160 pg 8 and line 167-172<br><br>Recruitment, exposure and follow-up are not applicable here.		
Participants	6	*(a) Cohort study* - Give the eligibility criteria, and the sources and methods of selection of participants. Describe methods of follow-up<br>*Case-control study* - Give the eligibility criteria, and the sources and methods of case ascertainment and control selection. Give the rationale for the choice of cases and controls<br>*Cross-sectional study* - Give the eligibility criteria, and the sources and methods of selection of participants	N/A	RECORD 6.1: The methods of study population selection (such as codes or algorithms used to identify subjects) should be listed in detail. If this is not possible, an explanation should be provided.<br><br>RECORD 6.2: Any validation studies of the codes or algorithms used to	6.1 pg 9 line 175-180<br><br>6.2 N/A

For peer review only - http://bmjopen.bmj.com/site/about/guidelines.xhtml

		(b) Cohort study - For matched studies, give matching criteria and number of exposed and unexposed<br>*Case-control study* - For matched studies, give matching criteria and the number of controls per case		select the population should be referenced. If validation was conducted for this study and not published elsewhere, detailed methods and results should be provided.<br><br>RECORD 6.3: If the study involved linkage of databases, consider use of a flow diagram or other graphical display to demonstrate the data linkage process, including the number of individuals with linked data at each stage.	6.3 Decided against given figure limits.
Variables	7	Clearly define all outcomes, exposures, predictors, potential confounders, and effect modifiers. Give diagnostic criteria, if applicable.	Outcomes - Pg 11 line 221-230 and Table 1.<br>Predictors – pg 12 line 263-267.<br>Control variables – pg 12 line 262 -267.	RECORD 7.1: A complete list of codes and algorithms used to classify exposures, outcomes, confounders, and effect modifiers should be provided. If these cannot be reported, an explanation should be provided.	Different data field names in different Trusts. Have explained how all variables derived – see Study Variables pg 9 line 191-
Data sources/ measurement	8	For each variable of interest, give sources of data and details of methods of assessment (measurement).	see Study Variables pg 9 line 191-		

For peer review only

		Describe comparability of assessment methods if there is more than one group			
Bias	9	Describe any efforts to address potential sources of bias	Sensitivity analysis conducted – line 245-246 pg 12		
Study size	10	Explain how the study size was arrived at	(Data was collected over a one year period in order to ensure that seasonal variation was fully accounted for.)		
Quantitative variables	11	Explain how quantitative variables were handled in the analyses. If applicable, describe which groupings were chosen, and why	See "Study variables" pg 9 line 191-<br><br>No groupings.		
Statistical methods	12	(a) Describe all statistical methods, including those used to control for confounding<br>(b) Describe any methods used to examine subgroups and interactions<br>(c) Explain how missing data were addressed<br>(d) *Cohort study* - If applicable, explain how loss to follow-up was addressed<br>*Case-control study* - If applicable, explain how matching of cases and controls was addressed<br>*Cross-sectional study* - If applicable, describe analytical methods taking account of sampling strategy<br>(e) Describe any sensitivity analyses	a) pg 12 line 259 – 263.<br><br>b) Subgroups – accounted for in nested multilevel structure. Interactions - pg 18 line 273 -274<br><br>c) pg 10 line 198 for missing SNCT assessments.<br><br>d) N/A<br>e) Line 245-246, pg 12.		

For peer review only

Data access and cleaning methods		..		RECORD 12.1: Authors should describe the extent to which the investigators had access to the database population used to create the study population.<br><br>RECORD 12.2: Authors should provide information on the data cleaning methods used in the study.	12.1 This question is aimed at participant-based research and related ethical issues which do not apply here.<br>12.2 See Data cleaning and Analysis pg 11 line 233-.
Linkage		..		RECORD 12.3: State whether the study included person-level, institutional-level, or other data linkage across two or more databases. The methods of linkage and methods of linkage quality evaluation should be provided.	12.3 pg 14 line 299-301
**Results**					
Participants	13	(a) Report the numbers of individuals at each stage of the study (*e.g.*, numbers potentially eligible, examined for eligibility, confirmed eligible, included in the study, completing follow-up, and analysed)<br>(b) Give reasons for non-participation at each stage.	13. We did not have participants as such. Have explained which wards are eligible, see line 175-180 pg 9.<br>13b & c) NA	RECORD 13.1: Describe in detail the selection of the persons included in the study (*i.e.,* study population selection) including filtering based on data quality, data availability and	13.1 NA

For peer review only

		(c) Consider use of a flow diagram		linkage. The selection of included persons can be described in the text and/or by means of the study flow diagram.	
Descriptive data	14	(a) Give characteristics of study participants (*e.g.*, demographic, clinical, social) and information on exposures and potential confounders<br>(b) Indicate the number of participants with missing data for each variable of interest<br>(c) *Cohort study* - summarise follow-up time (*e.g.*, average and total amount)	14a. Table 2<br>14b. line 298-301 pg 14 describes missing data and sample size. We did not have "participants" as such.<br>14c. NA		
Outcome data	15	*Cohort study* - Report numbers of outcome events or summary measures over time<br>*Case-control study* - Report numbers in each exposure category, or summary measures of exposure<br>*Cross-sectional study* - Report numbers of outcome events or summary measures	15. line 331-334 pg 16.		
Main results	16	(a) Give unadjusted estimates and, if applicable, confounder-adjusted estimates and their precision (e.g., 95% confidence interval). Make clear which confounders were adjusted for and why they were included<br>(b) Report category boundaries when continuous variables were categorized	16a. Table 4 pg 17 odds ratios and 95% confidence intervals.<br>b. NA<br>c. NA		

For peer review only

		(c) If relevant, consider translating estimates of relative risk into absolute risk for a meaningful time period			
Other analyses	17	Report other analyses done—e.g., analyses of subgroups and interactions, and sensitivity analyses	Pg 18 line 381-384		
**Discussion**					
Key results	18	Summarise key results with reference to study objectives	See conclusions pg 24.		
Limitations	19	Discuss limitations of the study, taking into account sources of potential bias or imprecision. Discuss both direction and magnitude of any potential bias	Pg 23 Limitations section.	RECORD 19.1: Discuss the implications of using data that were not created or collected to answer the specific research question(s). Include discussion of misclassification bias, unmeasured confounding, missing data, and changing eligibility over time, as they pertain to the study being reported.	N/A
Interpretation	20	Give a cautious overall interpretation of results considering objectives, limitations, multiplicity of analyses, results from similar studies, and other relevant evidence	See conclusions pg 24.		
Generalisability	21	Discuss the generalisability (external validity) of the study results	Pg 24 line 508-509.		
**Other Information**					

For peer review only

Funding	22	Give the source of funding and the role of the funders for the present study and, if applicable, for the original study on which the present article is based	See funding statement pg 25		
Accessibility of protocol, raw data, and programming code		..		RECORD 22.1: Authors should provide information on how to access any supplemental information such as the study protocol, raw data, or programming code.	22.1 Protocol supplied as supplementary material as requested by journal. Link to data provided in Data sharing statement pg 26.

*Reference: Benchimol EI, Smeeth L, Guttmann A, Harron K, Moher D, Petersen I, Sørensen HT, von Elm E, Langan SM, the RECORD Working Committee. The REporting of studies Conducted using Observational Routinely-collected health Data (RECORD) Statement. *PLoS Medicine* 2015; in press.

*Checklist is protected under Creative Commons Attribution (CC BY) license.

For peer review only
